# Histone lactylation promotes multidrug resistance in hepatocellular carcinoma by forming a positive feedback loop with PTEN

**DOI:** 10.1038/s41419-025-07359-9

**Published:** 2025-01-31

**Authors:** Yuan Zeng, Haoran Jiang, Zhoufeng Chen, Jun Xu, Xiangting Zhang, Weimin Cai, Xianjie Zeng, Peipei Ma, Rong lin, Huilin Yu, Yuanhang He, Huiya Ying, Ruoru Zhou, Xiao Wu, Fujun Yu

**Affiliations:** 1 Department of Gastroenterology, the First Affiliated Hospital of Wenzhou Medical University, Wenzhou, Zhejiang China; 2 Department of Radiation Oncology, the First Affiliated Hospital of Wenzhou Medical University, Wenzhou, Zhejiang China; 3 Department of Urology, the First Affiliated Hospital of Wenzhou Medical University, Wenzhou, Zhejiang China; 4 School of Pharmaceutical Science, Fujian Medical University, Fujian, China; 5 The First Clinical Medical College, Wenzhou Medical University, Wenzhou, Zhejiang China

**Keywords:** Cancer metabolism, Gastrointestinal cancer

## Abstract

FOLFOX (5-fluorouracil, oxaliplatin, folinic acid) is a standard treatment for hepatocellular carcinoma, but its efficacy is often limited by drug resistance, the underlying mechanisms of which remain unclear. In this study, oxaliplatin (OXA)- and 5-fluorouracil (5-Fu)-resistant hepatocellular carcinoma cell lines were established, and enhanced glycolytic activity was identified in resistant cells. Inhibiting glycolysis effectively suppressed the malignant behavior of both OXA- and 5-Fu-resistant cells. Mechanistically, active glycolysis induced elevated levels of lactylation, predominantly histone lactylation, with H3K14la playing a key role in regulating gene expression. The ubiquitin E3 ligase NEDD4 was identified as a downstream target of H3K14la. Furthermore, NEDD4, regulated by histone lactylation, interacted with PTEN to mediate its ubiquitination and subsequent degradation. The downregulation of PTEN formed a positive feedback loop, further driving the malignant progression of OXA- and 5-Fu-resistant cells. This study elucidates a shared mechanism underlying OXA and 5-Fu resistance in hepatocellular carcinoma and highlights a promising therapeutic target for overcoming clinical chemotherapy resistance.

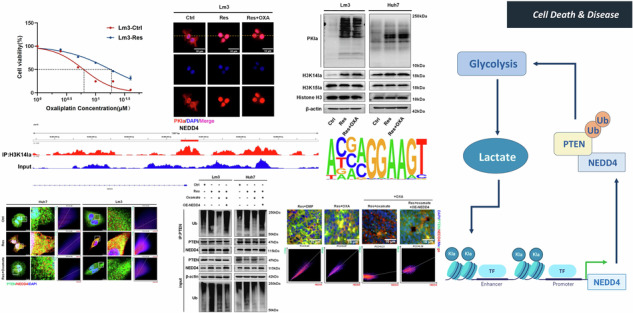

## Introduction

Liver cancer is one of the leading causes of cancer-related death worldwide, and hepatocellular carcinoma (HCC) is the most prevalent type of primary liver cancer and the third leading cause of cancer-related mortality according to recent statistics [1, 2]. Hepatic arterial infusion chemotherapy (HAIC) with oxaliplatin (OXA), fluorouracil (5-Fu), and leucovorin (FOLFOX) has been widely used in HCC patients [3, 4]. However, its effectiveness is limited, largely due to resistance [5]. Therefore, identifying the molecular mechanisms of OXA and 5-Fu resistance is a prerequisite for improving the prognosis of patients with HCC [6].

The Warburg effect was originally described as an increase in lactate production in cancer, and it has been confirmed to be associated with various malignant behaviors in HCC [7, 8]. In recent years, lactate-derived histone lysine lactylation has been proven to be a novel epigenetic modification and demonstrated to directly increase gene transcription within chromatin [9]. Studies have shown that histone or nonhistone lactylation plays a role in melanoma [10], colon cancer [11] and pancreatic ductal adenocarcinoma [12] by regulating m6A modification, cellular immunity and oncogene expression. Although the regulation of lactylation has been observed in HCC, the precise molecular mechanism underlying its role in OXA- and 5-Fu-resistance remains unclear.

Neuronal precursor cell-expressed developmentally downregulated 4 (NEDD4) is a significant member of the HECT domain E3 ligase family in eukaryotes [13], and plays a key role in various cellular processes through ubiquitination-mediated degradation of various substrates [14]. To date, numerous studies have reported the involvement of NEDD4 in the regulation of tumor proliferation [15], metastasis [16], and stemness [17]. However, whether NEDD4 is also involved in the development of OXA- and 5-Fu-resistant HCC remains unclear.

In this study, we found that histone lactylation regulated the expression of NEDD4, enhanced the degradation of the oncogene PTEN, promoted glycolysis and OXA/5-Fu resistance, and participated in other malignant behaviors of HCC.

## Materials and methods

### Cell lines and culture conditions

Human HCC-LM3 (Lm3) and Huh7 cells were obtained from the Cell Bank of the Chinese Academy of Sciences (Shanghai, China) and cultured in DMEM (Gibco, #11995040, USA) supplemented with 10% fetal bovine serum (FBS, Gibco, #16140089, USA) and 1% penicillin/streptomycin (NCM Biotech, #C40050, China). The cells were incubated at 37 °C in a CO_2_ incubator with 5% CO_2_. All cells were STR authenticated and free from mycoplasma contamination before use.

### Generation of OXA- and 5-Fu-resistant cells

Both OXA and 5-Fu were purchased from Macklin, China (61825-94-3, 51-21-8). OXA-resistant cells were generated as described previously [18, 19], Briefly, Lm3 and Huh7 cells were grown to 60–70% confluence, harvested with trypsin, and seeded in T25 cell culture flasks. After 24 h, the medium was replaced with DMEM containing 10% FBS and 1 μM OXA. After 48 h, the medium was changed and drug treatment was terminated. After 24 h of recovery and when the surviving cell population reached 80% confluence, cells were passaged and exposed to 1 μM OXA for another 48 h. The above steps were repeated for 6 cycles with 2, 4, 6, 8, or 10 μM OXA for Lm3 cells and 8 cycles with 2, 4, 6, 8, 10, 20, or 25 μM OXA for Huh7cells. After the cells developed stable resistance to OXA, they were designated Lm3-Res and Huh7-Res cells.

To generate 5-Fu-resistant HCC cells [20, 21], Lm3 and Huh7 cells were cultured to 60–70% confluence, digested with trypsin, and inoculated into T25 cell culture flasks. After 24 h, the medium was replaced with DMEM containing 10% FBS and 5 μM 5-Fu. After one week of culture, the drug was discontinued. Once the surviving cells resumed exponential growth after being relieved of 5-Fu pressure, they were passaged upon reaching 80% confluence, and then treated with 10 μM 5-Fu for one week. Lm3 cells were treated with 10, 20, 40, or 60 μM 5-Fu for five cycles, and Huh7 cells were treated with 10, 20, 40, 60, 80, or 100 μM 5-Fu for seven cycles. When the cells developed stable resistance to 5-Fu, they were designated Lm3-Res and Huh7-Res cells.

The resistant cells were frozen in liquid nitrogen, recovered after three passages, and their biological characteristics were analyzed in subsequent experiments. One week before the experiment, OXA- and 5-Fu-resistant cells were cultured in media without OXA or 5-Fu. All experiments were performed with exponentially growing cells, and each experiment was conducted with three biological replicates of the same resistant cell line.

### Establishment of the in vivo nude mouse model

Twenty 4-week-old male and twenty 4-week-old female BALB/c nude mice (obtained from the Animal Experimental Center of the First Affiliated Hospital of Wenzhou Medical University) were housed in a pathogen-free environment and randomly assigned to one of four groups.

A mouse tumor model was established via subcutaneous injection. After randomization, 1 × 10^6^ OXA-resistant Lm3 OE-NEDD4 or OE-NC cells were injected subcutaneously. Seven days later, dimethylformamide, OXA (5 mg/kg) and oxamate (750 mg/kg) were intraperitoneally injected weekly. The tumor diameter was recorded every 7 days using a Vernier calliper, and the tumor volume was calculated using the following formula: volume (mm^3^) = 0.5 × width^2^ × length. After 35 days, the mice were euthanized by CO_2_ asphyxiation. The transplanted tumors were then excised and weighed. After euthanasia, gross images were taken. Some tissues were paraffin embedded and sliced into sections for immunofluorescence detection, some were used for protein extraction for western blot analysis, and the remaining tissues were stored at −80 °C for future use.

The animal experiments were authorized by the Institutional Animal Care and Use Committee of the Animal Experiment Center at the First Affiliated Hospital of Wenzhou Medical University (WYYY-AEC-2022-029).

### Quantitative real-time polymerase chain reaction (qRT‒PCR)

Total RNA was extracted from the cells using TRIzol reagent (Takara Biotech Co., Ltd.). The concentration of the extracted RNA was measured with a NanoDrop spectrophotometer (Thermo Fisher Scientific). Next, the RNA was reverse transcribed into complementary DNA (cDNA) using the PrimeScript RT Kit (Takara Biotech Co., Ltd.), following the manufacturer’s instructions. The cDNA was then used to perform RT-qPCR with the CellAmp™ Direct TB Green® qRT‒PCR Kit (Takara Biotech Co., Ltd.). Gene expression levels were analyzed using the ΔΔCT method and normalized to the *β-actin* mRNA levels in the cells. The primers used are listed in Supplementary Table 1.

### Western blots

Total protein from tumor tissues and cells was extracted using cold RIPA lysis buffer (Solarbio, China) supplemented with a phosphatase inhibitor cocktail (GlpBio, USA) and PMSF (Beyotime, China). The protein concentration was measured with a BCA assay (Beyotime, China), and 20 μg of total protein was used for immunoblotting. The protein lysates were separated by 12.5% SDS‒PAGE and transferred to a PVDF membrane with a pore size of 0.22 µM (Millipore, Massachusetts, USA). The membrane was blocked for 20 min with fast blocking buffer (Beyotime, Shanghai, China). Proportionally diluted antibodies were then incubated overnight. The membrane was washed three times with Tris buffer containing Tween solution (TBST: 50 mmol/L Tris-HCl, pH 8.0, 150 mmol/L NaCl, and 0.1% Tween-20), incubated with HRP-IgG antibody at room temperature for 2 h, and washed again with TBST. The ECL signal was visualized using an Omni-ECL™ Enhanced Photochemiluminescence Kit (Epizyme Biomedical Technology, #SQ101, China). Protein intensity was analyzed using ImageJ software. Protein expression levels were normalized first to β-actin as the internal reference, and then to the control group to calculate the relative expression levels. The antibodies used in this study are listed in Supplementary Table 2.

### Cell viability assay

Lm3, Huh7, Lm3-Res, and Huh7-Res cells were seeded into 96-well plates at a density of 5000 cells per well and incubated overnight. All CCK8 experiments were performed in triplicate and repeated three times. To measure the IC50 of 5-Fu, concentrations of 0, 10, 20, 40, 80, or 150 μM 5-Fu were applied to the cells for 24 h. Similarly, to determine the IC50 of OXA, OXA at concentrations of 0, 5, 10, 20, 40, or 80 μM were applied to the cells for 24 h, and 10 μL of Cell Counting Kit 8 solution (CCK8, MedChemExpress, USA) was added to each well for 2 h. The absorbance of each well was quantified at 450 nm using a microplate reader (Bio-Tek, USA).

### Immunofluorescence assay

For immunofluorescence, the cells were fixed with 4% paraformaldehyde and permeabilized with 0.5% Triton X-100. The cells and dewaxed tumor tissue sections were incubated with primary antibodies overnight in a humidified chamber at 4 °C. Subsequently, the cells and tissue sections were incubated with fluorescein-labeled secondary antibodies according to the manufacturer’s instructions, followed by incubation with DAPI (Biosharp, #BL520B, China), and the coverslip were sealed with nail polish. Fluorescence images were obtained using a fluorescence microscope (Leica, DM6 B, 40× objective), a confocal microscope (Leica, STELLARIS 5, 100x objective), and filters appropriate for the wavelengths used. The filter cube set included DAPI (EX 350/50, DC 400, EM 460/50), L5 (EX 480/40, DC 505, EM 527/30), and N21 (EX 538/46, DC 580, EM LP 590) (Leica, Germany). Using random field selection and sample blinding, ten images were captured for each sample. Representative fields of view were then selected for presentation.

Immunofluorescence colocalization was performed using the “Coloc2” module in ImageJ. Pearson’s correlation coefficient (PCC) was calculated; the closer the PCC is to 1, the stronger the colocalization. For cell fluorescence colocalization, individual cell ROIs were selected for Pearson’s analysis, while for tissue fluorescence, the entire field of view was used for analysis [22]. Approximately 500 cells were selected for quantification in each sample and the average value was taken. A total of three biological replicates were quantified. The antibodies used in this study are listed in Supplementary Table 2.

### Measurement of ROS levels

Intracellular reactive oxygen species (ROS) levels were measured using an ROS detection kit (Beyotime, Shanghai, China) according to the manufacturer’s instructions. The cells were digested with trypsin, resuspended, incubated with 10 mM 2,7-dichlorofluorescein diacetate (DCFH-DA) at 37 °C for 10 min, washed three times with serum-free DMEM, and finally analyzed using a flow cytometer (Beckman Coulter). The results were analyzed using FlowJo V10 software. The gating strategy initially involved gating cells by the forward scatter area and side scatter area to remove debris, followed by gating cells by the forwards scatter area and the forwards scatter height to exclude doublets.

### Transwell assay

Transwell migration assays were performed by seeding 2 × 10^4^ cells with 200 μL of serum-free DMEM into the upper chamber of a Transwell system (Corning, USA), and the lower chamber contained 600 μL of DMEM supplemented with 10% FBS. After 48 h of culture, all cells on the upper surface of the membrane were removed, and the cells on the lower surface were fixed and stained with crystal violet (Beyotime, Shanghai, China). Briefly, the bottoms of the Transwell were fixed by immersion in 4% paraformaldehyde for 20 min, followed by immersion in crystal violet dye for 10 min, and then washing with PBS solution on a shaker for 15 min × 3. After natural air drying, images were taken using an inverted phase contrast microscope (Olympus, Tokyo, Japan). Ten fields of view were randomly selected for each sample for quantitative analysis, and the average value was taken. A total of three biological replicates were performed for quantitative analysis. Representative images of the lower surface were quantified using ImageJ.

### Statistical analyses

The results were imported into GraphPad Prism 8.0.2 software for analysis. Data comparisons between two groups were performed using the Student’s *t* test, and comparisons of multiple groups were analyzed using the one-way analysis of variance (ANOVA) followed by Tukey’s post hoc test. *P* < 0.05 was considered statistically significant. (*p < 0.05, **p < 0.01; ***p < 0.001, ns: not significance.) More methods have been provided in the Supplementary Methods.

## Results

### Increased histone lactylation in OXA-resistant HCC cells

**Fig. 1:**
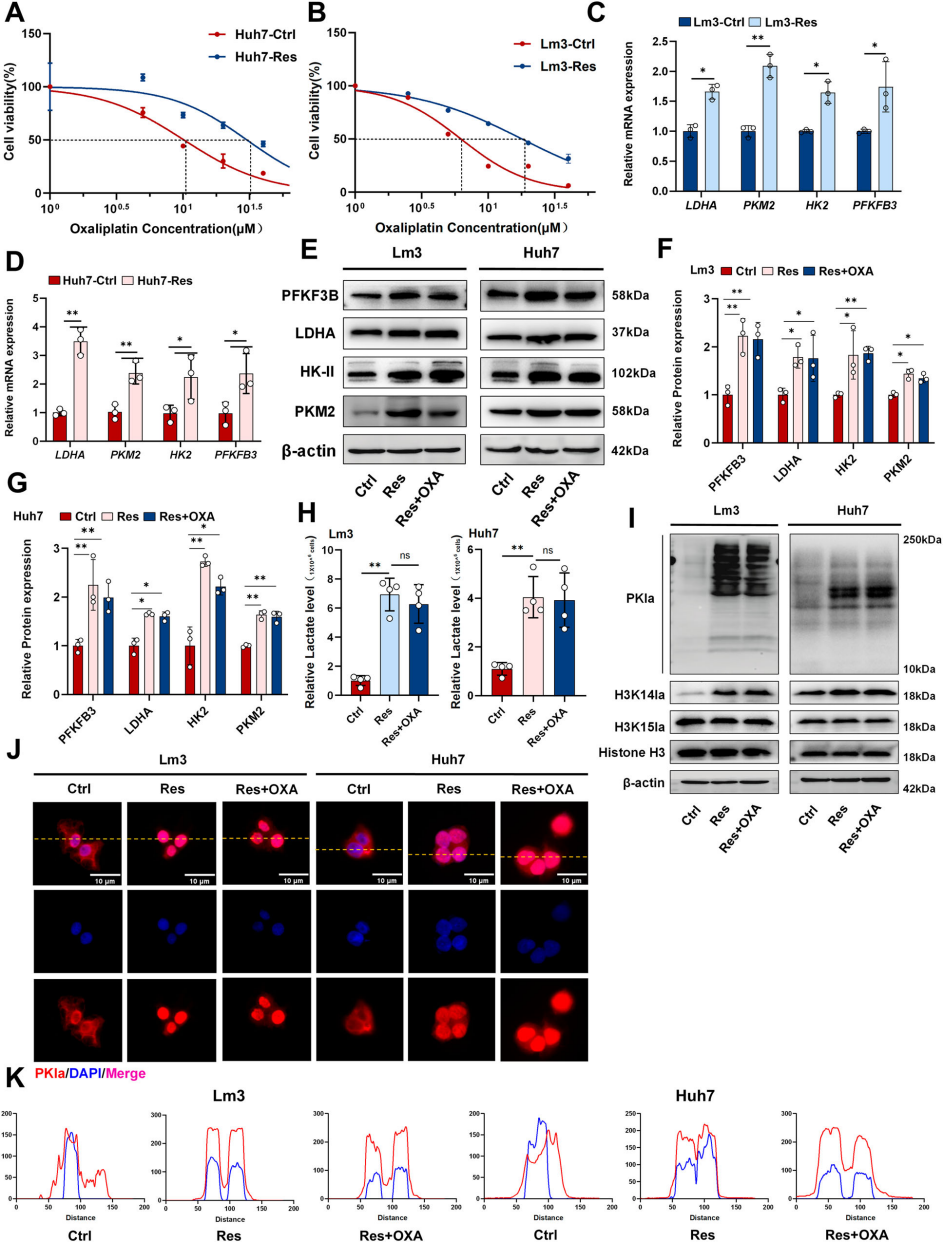
OXA-resistant HCC cells exhibit active histone lactylation. **A** IC50 values of OXA-sensitive (Ctrl) and OXA-resistant strains (Res) in Lm3 cells. (The dashed line represents the drug concentration at which 50% of cells survive.) **B** IC50 values of OXA-sensitive (Ctrl) and OXA-resistant strains (Res) in Huh7. (The dashed line represents the drug concentration at which 50% of cells survive.) **C**, **D** Glycolysis-related enzyme mRNA levels detected via qRT‒PCR in Lm3 and Huh7 cells (n = 3). **E**–**G** Glycolysis-related enzyme protein levels in Lm3 and Huh7 cells were detected via western blotting (n = 3). **H** Intracellular lactate production occurs in OXA-sensitive (Ctrl) and OXA-resistant (Res) cells in Lm3 and Huh7, respectively (n = 3). **I** Western blot analysis of PKla and histone lactylation protein levels in OXA-resistant cells. **J**, **K** Immunofluorescence confirmed the predominant intracellular localization of lactylation. Scale bar = 10 µm. *P < 0.05, **P < 0.01, ***P < 0.001, ****P < 0.0001.

To elucidate the underlying mechanism of OXA resistance, we established OXA-resistant cell lines. The results of the CCK8 assay indicated that the IC50 of the Lm3 OXA-resistant cell line changed from 6.24 μM in the sensitive strain (Lm3-Ctrl) to 17.94 μM in the resistant strain (Lm3-Res). Similarly, in Huh7 cells, the IC50 increased from 10.16 μM (Huh7-Ctrl) to 30.19 μM (Huh7-Res) (Fig. 1A, B). Numerous studies have demonstrated a close correlation between active aerobic glycolysis, lactylation and drug resistance in tumor cells [23, 24]. The results of the qRT‒PCR analysis showed that the mRNA levels of enzymes related to glycolysis were significantly greater in OXA-resistant cells (Res) than in OXA-sensitive cells (Ctrl) (Fig. 1C, D). Similarly, western blot analysis revealed significant elevation in the protein levels of PKM2, LDHA, HK2, and PFKFB3 (Fig. 1E–G). Measurement of the lactate levels in both sensitive strains and OXA-resistant cells treated with OXA at similar concentrations (Lm3: 6 μM, Huh7: 14 μM) showed that OXA treatment increased intracellular lactate levels by 100% in sensitive cells (Supplementary Fig. S1.A). In contrast, the lactate levels in OXA-resistant cells were 400% to 500% higher than those in sensitive cells and showed no significant changes after OXA treatment (Fig. 1H), which is consistent with the findings of a previous study [19]. These findings demonstrate that glycolysis was active in OXA-resistant HCC cells. Western blot analysis confirmed that the level of pan-lactylation (PKla) was higher in OXA-resistant cells than that in OXA-sensitive cells (Fig. 1I). Immunofluorescence revealed that lactylation modification primarily occurred in the nuclei of OXA-resistant cells, suggesting that lactylation modification of histones may be a primary modification within OXA-resistant cells (Fig. 1J, K). To further clarify the histone lactylation sites, we detected the changes of H3K15a and H3K14a proteins respectively. Notably, the H3K14a protein level was significantly increased in OXA-resistant cells, whereas the H3K15a protein level did not significantly change, suggesting that H3K14a may play an essential role in OXA resistance (Fig. 1I). Previous studies have shown that ROS levels and antioxidant enzyme activities are generally increased in drug-resistant cancer cells [25]. Additionally, some studies have confirmed that complex regulatory mechanisms may exist between histone lactylation and ROS under different conditions [26–28]. To further clarify whether the increase in histone lactylation in drug-resistant cells was mediated by ROS, we used flow cytometry to detect sensitive cells, sensitive cells treated with OXA alone, and OXA-resistant cells. The results showed that ROS were significantly increased in cells treated with OXA alone, but not obvious in OXA-resistant cell lines (Supplementary Fig. S1.B, C). This may be due to a more robust intracellular redox system during the drug resistance process [25]. Additionally, when the ROS scavenger N-acetyl-L-cysteine (NAC) was used, we observed no significant change in H3K14la histone lactylation in drug-resistant cells (Supplementary Fig. S1.D, E). These findings suggest that ROS accumulation during the drug resistance process does not promote H3K14la expression, and the specific mechanism of increased histone lactylation in drug-resistant cells requires further investigation. These data indicate that a large portion of OXA-resistant cells are characterized by active glycolysis and lactylation modifications, which are likely the underlying mechanism of OXA resistance.

### Inhibition of histone lactylation suppressed the progression of OXA-resistant cells

**Fig. 2:**
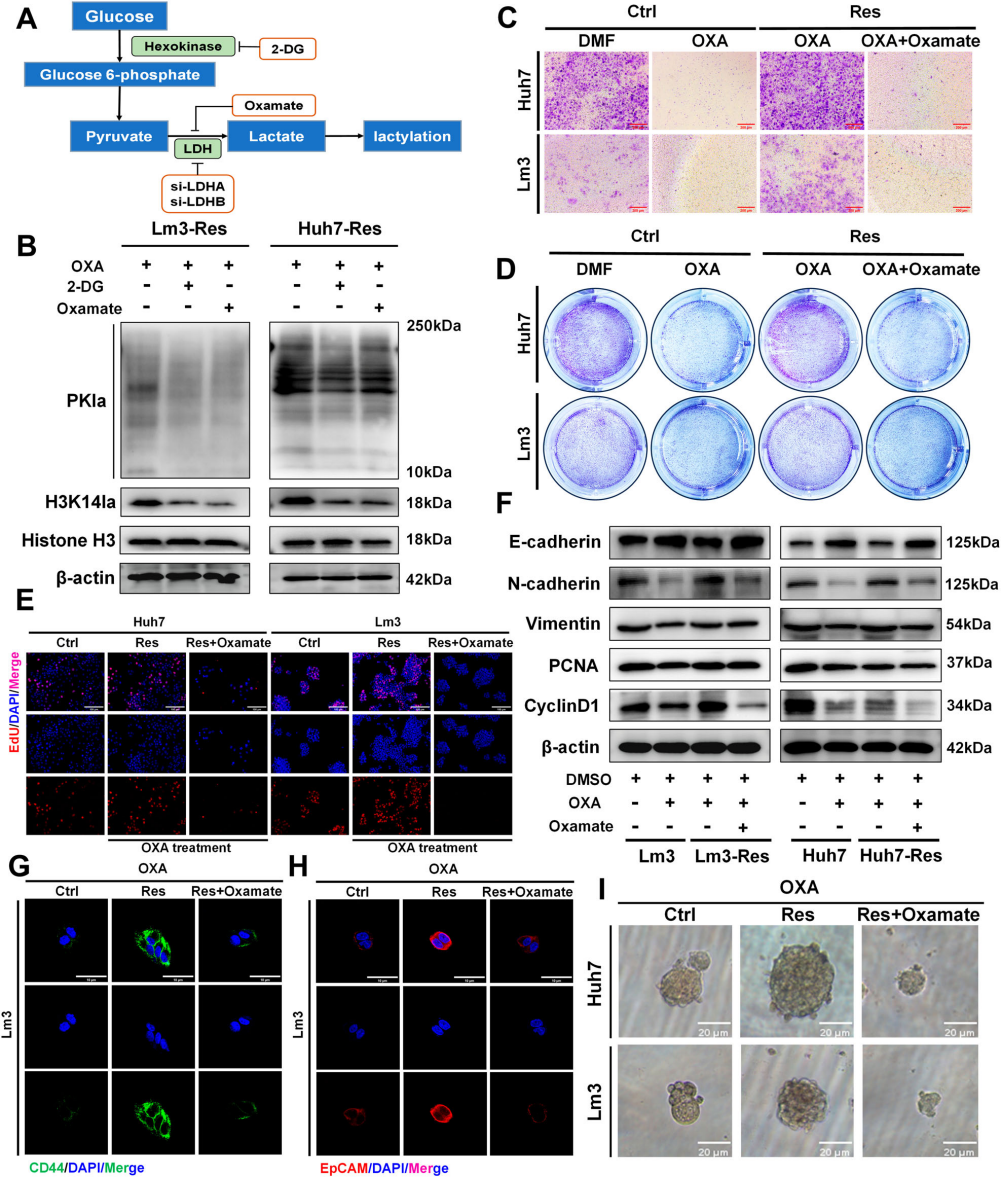
Targeting histone lactylation inhibits the malignant behavior of OXA-resistant cells. **A** Different inhibitors of the lactylation process. **B** Western blot analysis of PKla and histone lactylation in oxamate and 2-DG treated OXA-resistant cells (Lm3-Res and Huh7-Res). **C** Transwell assays identified OXA-sensitive (Ctrl) and OXA-resistant (Res) cell migration, Scale bar = 200 µm. **D** Colony formation experiments confirmed the proliferation of OXA-sensitive (Ctrl) and OXA -resistant (Res) cells. **E** Resistant cells were treated with OXA and the proliferation of sensitive and resistant cells was assayed with EdU, scale bar = 100 µm. **F** Western blot analysis revealed changes in the levels of EMT-related proteins and proliferation-related proteins in both sensitive and resistant cells. **G**, **H** Both sensitive and resistant cells were treated with OXA, and immunofluorescence was used to detect changes in the protein levels of the stemness-related proteins CD44 and EpCAM in Lm3 cells, scale bar = 10 µm. **I** Both sensitive and resistant cells were treated with OXA and a spheroid formation assay was performed to dectect tumor cell stemness, scale bar = 20 µm. Lm3- and Huh7-sensitive and -resistant cells were treated with 6 μM and 14 μM OXA, respectively. *P < 0.05, **P < 0.01, ***P < 0.001, ****P < 0.0001.

To determine whether histone lactylation is involved in the molecular mechanisms of regulating OXA resistance, we employed various methods to reduce the level of intracellular histone lactylation (Fig. 2A). The production of intracellular lactate was significantly reduced in a dose-dependent manner after treatment with the nonmetabolizable glucose analogs 2-deoxy-d-glucose (2-DG), and oxamate, as well as si-LDHA and si-LDHB (Supplementary Fig. S2.A, B). Additionally, both the intracellular and histone lactylation levels were significantly reduced (Fig. 2B and Supplementary Fig. S2.C). To further elucidate the malignant behavior of OXA-resistant cells, we treated both OXA-sensitive and OXA-resistant cells with a low concentration (IC75) of OXA. All Lm3 and Huh7 cells were treated with 6 μM and 14 μM OXA, respectively. Transwell experiments revealed that migration of the resistant strain was not significantly inhibited by OXA compared with that of the sensitive strain. However, the number of migrating cells was significantly reduced after the addition of the histone lactylation inhibitor (Fig. 2C and Supplementary Fig. S2.D). The colony formation assay showed that OXA-resistant strains formed fewer colonies after inhibition of histone lactylation (Fig. 2D and Supplementary Fig. S2.E). Similar results were also observed in the EdU experiment (Fig. 2E and Supplementary Fig. S2.F). The western blot showed that compared with those of the sensitive strain, OXA had no effect on the expression levels of the EMT-related proteins E-cadherin, N-cadherin and vimentin in the resistant strain. However, the addition of oxamate significantly enhanced the effects of OXA on OXA-resistant strains and markedly reduced the expression levels of the abovementioned proteins. Similarly, comparable results were observed for the expression levels of the proliferation-related proteins PCNA and CyclinD1 (Fig. 2F and Supplementary Fig. S2.G). Finally, we conducted further investigations to explore whether histone lactylation is involved in the regulation of cell stemness. Immunofluorescence experiments demonstrated that the expression of CD44 and EpCAM proteins was effectively suppressed by oxamate in OXA-resistant strains (Fig. 2G–H and Supplementary Fig. S3.A). Additionally, a sphere-formation assay demonstrated that inhibiting histone lactylation significantly reduced the cell spheroid size (Fig. 2I). In summary, histone lactylation may play a crucial role in regulating the proliferation, migration, stemness, and other malignant behaviors of OXA-resistant cells. Inhibiting histone lactylation may effectively increase the sensitivity to OXA chemotherapy, thus inhibiting tumor progression.

### Histone lactylation regulates ubiquitination in OXA-resistant strains

**Fig. 3:**
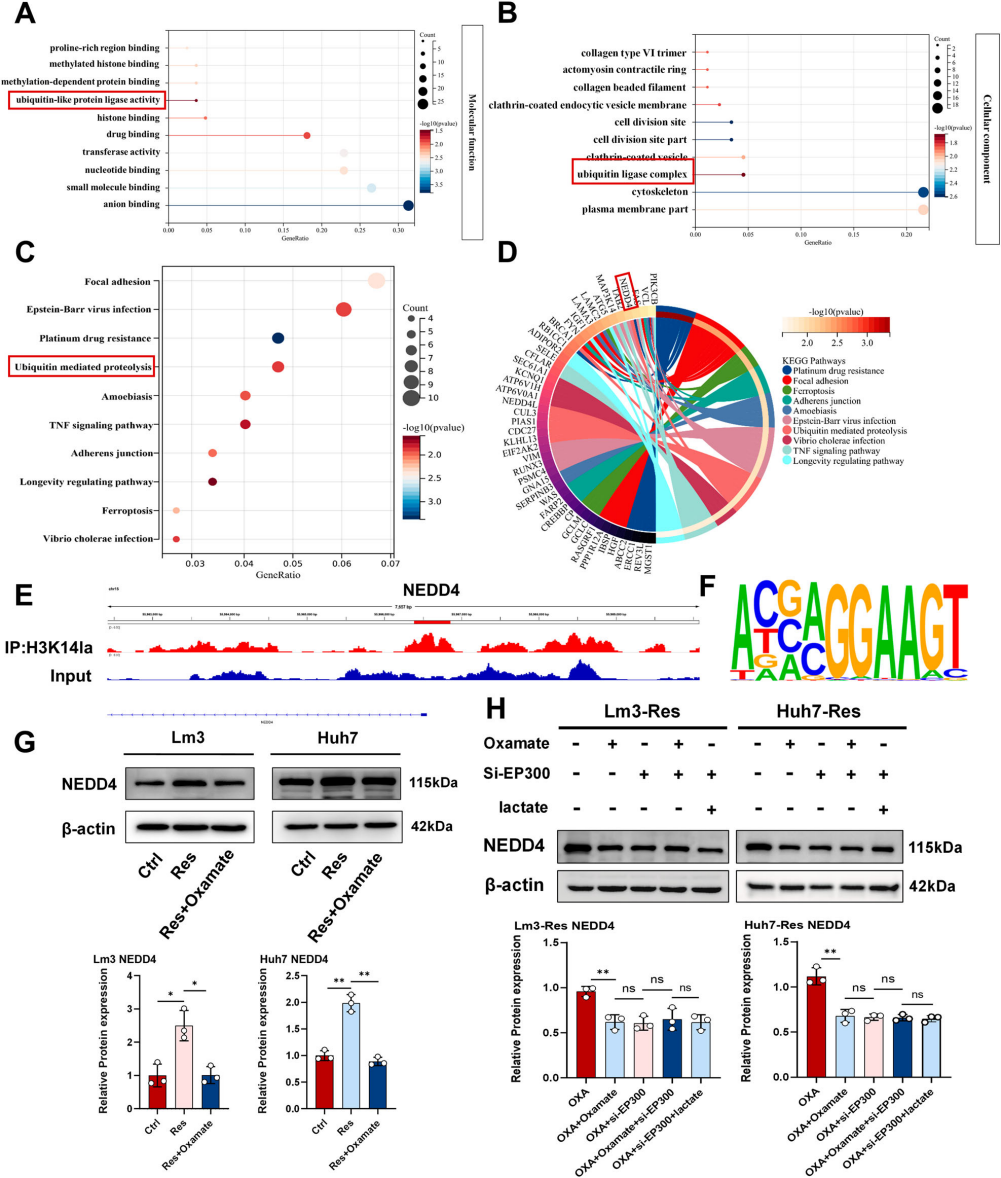
ChIP-Seq reveals the involvement of histone lactylation in modifying NEDD4 in OXA-resistant cells. **A**, **B** GO analysis confirmed that histone lactylation was involved in regulating cell function. **C**, **D** KEGG analysis confirmed that histone lactylation primarily regulated signaling pathways. **E** Integrative Genomics Viewer (IGV) trace of NEDD4 from ChIP-seq analysis. **F** Enriched promoter region bases. **G** NEDD4 protein levels were detected by western blotting (n = 3). **H** NEDD4 protein levels were detected by western blotting after the addition of si-EP300, oxamate or lactate (n = 3). *P < 0.05, **P < 0.01, ***P < 0.001, ****P < 0.0001.

To clarify the regulatory role of histone lactylation in OXA-resistant strains, we first performed chromatin immunoprecipitation followed by sequencing (ChIP-seq) using anti-H3K14la antibodies in OXA-resistant Lm3 cells, and then performed gene ontology (GO) and Kyoto Encyclopedia of Genes and Genomes (KEGG) analyses. The results showed that histone lactylation was mainly involved in regulating the intracellular ubiquitination process (Fig. 3A, B and Supplementary Fig. S3.B). In addition, the KEGG enrichment analysis suggested that histone lactylation may play a regulatory role through ubiquitination-mediated protein degradation (Fig. 3C). To further study how histone lactylation affects the ubiquitination process in OXA-resistant strains, we screened the related ubiquitin E3 ligase NEDD4 in the KEGG database (Fig. 3D). Previous studies have suggested that NEDD4 plays a key role in a variety of cellular processes through ubiquitination-mediated degradation of multiple substrates [29]. To confirm whether the activation of NEDD4 transcription was dependent on H3K14la, we observed marked enrichment of the H3K14la signal specific to the promoter region of the NEDD4 gene (Fig. 3E). The base pair enrichment of the ChIP-seq promoter region is shown in Fig. 3F. Additionally, we used western blotting to detect the intracellular NEDD4 protein level, and found that the NEDD4 protein level remained unchanged in the sensitive strain treated with OXA alone, but was increased in the OXA-resistant strain, and decreased after protein lactylation in the inhibition group (Fig. 3G). Given that EP300 is the key enzyme for histone lactylation, we used oxamate, si-EP300 and a combination of the two to inhibit the intracellular histone lactylation levels in OXA-resistant cells. The result showed that the NEDD4 protein level was significantly reduced, whereas there was no statistically significant change in the protein level under the above treatments. Moreover, adding additional lactate after EP300 silencing did not restore the protein level of NEDD4 (Fig. 3H). Similarly, the inhibition of intracellular lactylation levels by 2-DG and oxamate resulted in a concurrent reduction in overall intracellular ubiquitination levels (Supplementary Fig. S3.D). These data suggest that the expression of the E3 ubiquitin ligase NEDD4 in OXA-resistant strains is positively regulated by histone lactylation.

### Histone lactylation mediates malignant progression of OXA-resistant strains through NEDD4

**Fig. 4:**
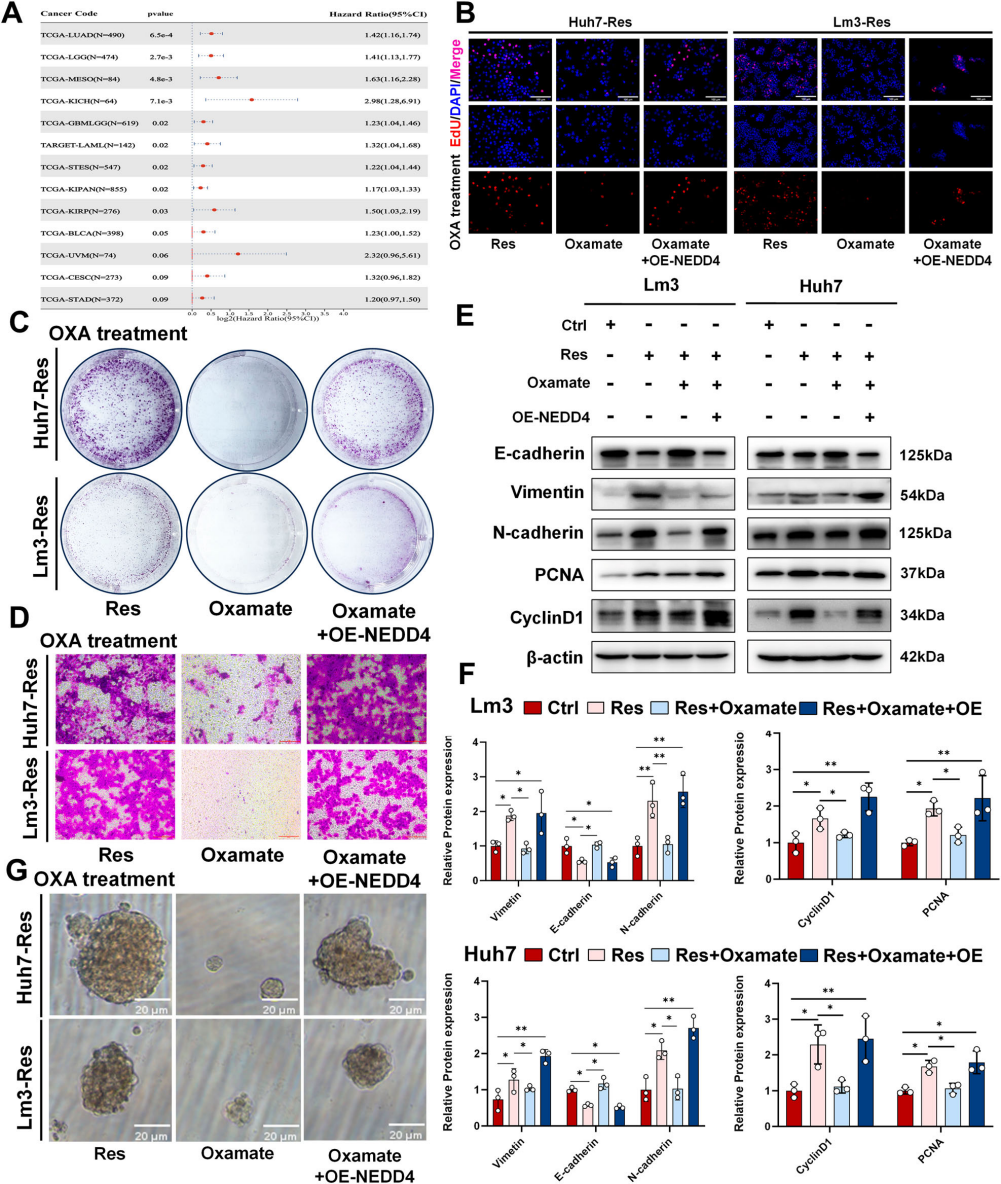
Histone lactylation mediates OXA resistance through NEDD4. **A** Analysis of the TCGA database to investigate the correlation between NEDD4 expression and the prognosis of patients with various tumors. After OXA-resistant cells (Res) were treated with low-dose OXA, they were given oxamate or OE-NEDD4. Cell proliferation was detected via **B** EdU (scale bar = 100 µm) and **C** colony formation, **D** cell migration was detected via Transwell assays (scale bar = 200 µm), and **G** cell stemness was detected via spheroid formation experiments (scale bar = 20 µm). **E**, **F** Western blotting was used to determine the expression of EMT-related proteins and proliferation-related proteins in OXA-sensitive (Ctrl) and OXA-resistant (Res) cells (n = 3). Lm3 and Huh7 OXA-resistant cells were treated with 6 μM and 14 μM OXA, respectively. *P < 0.05, **P < 0.01, ***P < 0.001, ****P < 0.0001.

Many studies have shown that NEDD4 plays a significant role in the progression of breast cancer [30], bladder cancer [31], and lung cancer [32]. In addition, the TCGA database shows that high expression of NEDD4 is associated with poor prognosis in patients with lung adenocarcinoma, multiple myeloma, and mesothelioma. However, the association between NEDD4 expression and patient prognosis in HCC remains unclear (Fig. 4A). Therefore, we verified the function of NEDD4 in HCC cells by silencing the expression of NEDD4 with siRNAs (si-NEDD4#1 and si-NEDD4#2). The EdU assay results showed that the proliferation of OXA-resistant cells was significantly reduced when NEDD4 was silenced, compared with that of control cells (Supplementary Fig. S3.E, F). Similar results were observed via western blotting, where the levels of the proliferation-related proteins PCNA and CyclinD1, as well as EMT-related proteins, were downregulated after NEDD4 silencing (Supplementary Fig. S3.G, H). Given that NEDD4 expression is regulated by H3K14la, we determined whether the tumor suppression induced by oxamate could be rescued by increasing NEDD4. After an OXA-resistant strain stably overexpressing NEDD4 was constructed using lentivirus and treated with OXA, we assessed alterations in cell proliferation and migration ability using EdU (Fig. 4B and Supplementary Fig. S4.A), colony formation (Fig. 4C and Supplementary Fig. S4.B), and Transwell assays (Fig. 4D and Supplementary Fig. S4.C). These results suggested that tumor behavior, which was suppressed by the inhibition of histone lactylation was effectively reversed after the overexpression of NEDD4. We subsequently observed alterations in the levels of proteins related to proliferation and EMT (Fig. 4E, F). The subsequent sphere-formation assay confirmed that the inhibition of cell stemness caused by histone lactylation inhibitors could be reversed by the overexpression of NEDD4 (Fig. 4G). Overall, our study found that the tumor suppressive effects of blocking histone lactylation could be hindered by OE-NEDD4, indicating that the tumor promotion effect mediated by histone lactylation was partly achieved through NEDD4.

### NEDD4-induced PTEN ubiquitination mediates OXA-resistance in HCC cells

**Fig. 5:**
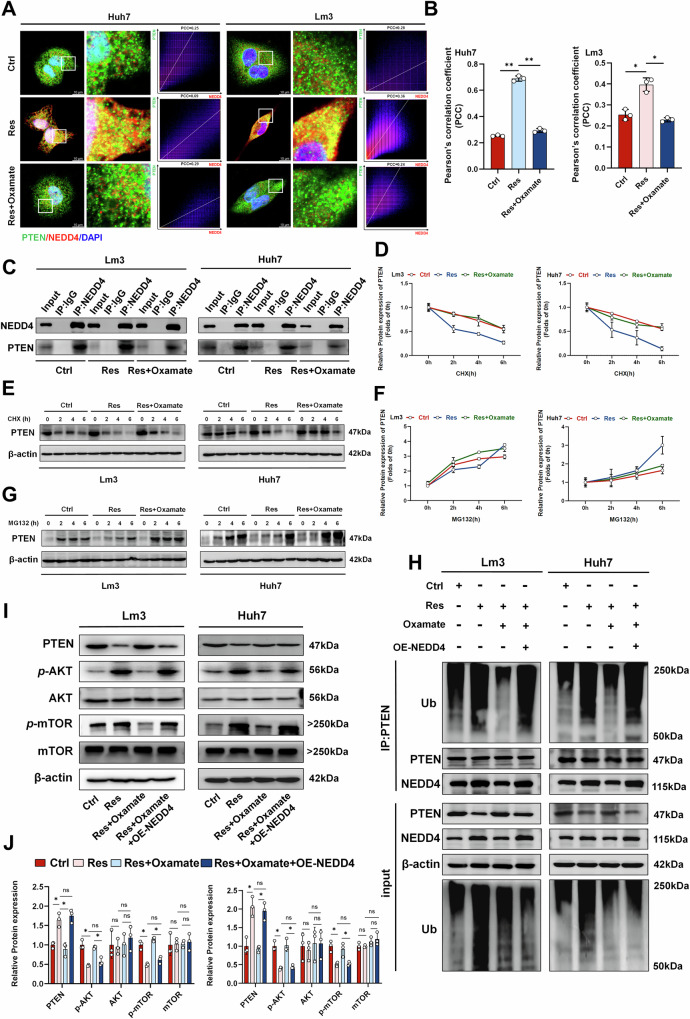
NEDD4 mediates PTEN ubiquitination to induce OXA resistance. **A** Immunofluorescence confirmed the colocalization of NEDD4 and PTEN in OXA-sensitive (Ctrl) and OXA-resistant (Res) cells, scale bar = 10 µm. The X-axis represents the pixel intensity of NEDD4 (red), and the Y-axis represents the pixel intensity of PTEN (green). **B** Colocalization was quantified via ImageJ (n = 3). **C** Co-IP confirmed that NEDD4 binds to PTEN in in OXA-sensitive (Ctrl) and OXA-resistant (Res) cells. **D**, **E** Western blotting was used to detect and quantify the half-life of PTEN after treatment with CHX alone. **F**, **G** Western blot analysis revealed changes in PTEN protein levels after treatment with MG132 alone. **H** Detection of NEDD4 binding and ubiquitination levels using western blotting after Co-IP with PTEN in OXA-sensitive (Ctrl) and OXA-resistant (Res) cells. **I**, **J** Western blot analysis of PTEN and AKT/mTOR protein levels (n = 3). PCC Pearson’s correlation coefficient *P < 0.05, **P < 0.01, ***P < 0.001, ****P < 0.0001.

Next, we further clarified how histone lactylation regulated the drug resistance mechanism of HCC through NEDD4. NEDD4, an E3 ubiquitin ligase, participates in regulating the ubiquitin degradation process of numerous proteins [33]. Numerous studies have shown that NEDD4 is involved in regulating the stability of PTEN [34–36]. Therefore, we investigated whether NEDD4 is also involved in mediating the ubiquitination and proteasomal degradation of PTEN in HCC. Western Blots showed that compared with that in the sensitive strain, the change in the PTEN protein level was not significant after treatment with OXA alone, whereas in the OXA-resistant strain, the PTEN protein level decreased and could be restored by oxamate (Supplementary Fig. S3.C). The immunofluorescence results indicated that the colocalization and binding of NEDD4 and PTEN in OXA-resistant strains were greater than those in sensitive strains, and this effect could be inhibited by histone lactylation inhibitors (Fig. 5A, B). Similar results were also observed in the Co-IP experiment (Fig. 5C). We further investigated the regulation of PTEN by the proteasome system. By employing the translation inhibitor cycloheximide (CHX) to inhibit intracellular protein translation, we observed a significant decrease in the half-life of the PTEN protein in the OXA-resistant strain compared with that of the control group. This phenomenon could also be blocked by histone lactylation inhibitors (Fig. 5D, E). The accumulation of PTEN in OXA-resistant cells was also significantly increased after treatment with a proteasome inhibitor (MG132) (Fig. 5F, G), indicating that PTEN in OXA-resistant cells is regulated mainly through the proteasome pathway. We further examined changes in PTEN ubiquitination levels, and found that the level of PTEN ubiquitination in OXA-resistant cells was significantly higher than that in OXA-sensitive cells. The overexpression of NEDD4 rescued the level of ubiquitination inhibited by oxamate (Fig. 5H) [37, 38]. These findings indicate that NEDD4 is involved in regulating the level of ubiquitinated PTEN in OXA-resistant cells. PTEN is one of the most frequently mutated or deleted genes in cancer [39, 40]. Loss of PTEN influences important cellular processes that are critical to cancer progression, including changes in survival, proliferation, energy metabolism, and cell structure [39]. PTEN has been found to interfere with tumor progression by inhibiting the PI3K/Akt/mTOR pathway [41]. Western blot analysis revealed that the PI3K/Akt/mTOR pathway was significantly activated in OXA-resistant cells compared with that in sensitive cells. After inhibiting histone lactylation, the levels of intracellular *p*-AKT and *p*-mTOR proteins were subsequently reduced. However, overexpression of NEDD4 restored the above protein levels in cells (Fig. 5I, J). Additionally, our ChIP data revealed the presence of NEDD4L, another member of the NEDD4 family. Previous studies have shown that NEDD4L has a complex regulatory relationship with PTEN [42, 43]. To determine whether NEDD4L is also involved in the regulation of PTEN in OXA-resistant HCC, we used si-NEDD4L to silence NEDD4L expression in cells (Supplementary Fig. S5.A). We then examined the expression of PTEN and its downstream molecules. The results showed that PTEN protein levels did not change significantly after si-NEDD4L treatment, and the histone lactylation inhibitor continued to inhibit PTEN degradation even after NEDD4L was silenced (Supplementary Fig. S5.B). Collectively, these results confirmes that histone lactylation promotes resistance to OXA by upregulating NEDD4-mediated ubiquitination and degradation of PTEN, thereby activating the PI3K/Akt/mTOR signaling pathway.

### Histone lactylation mediates 5-Fu resistance through NEDD4-induced PTEN ubiquitination

**Fig. 6:**
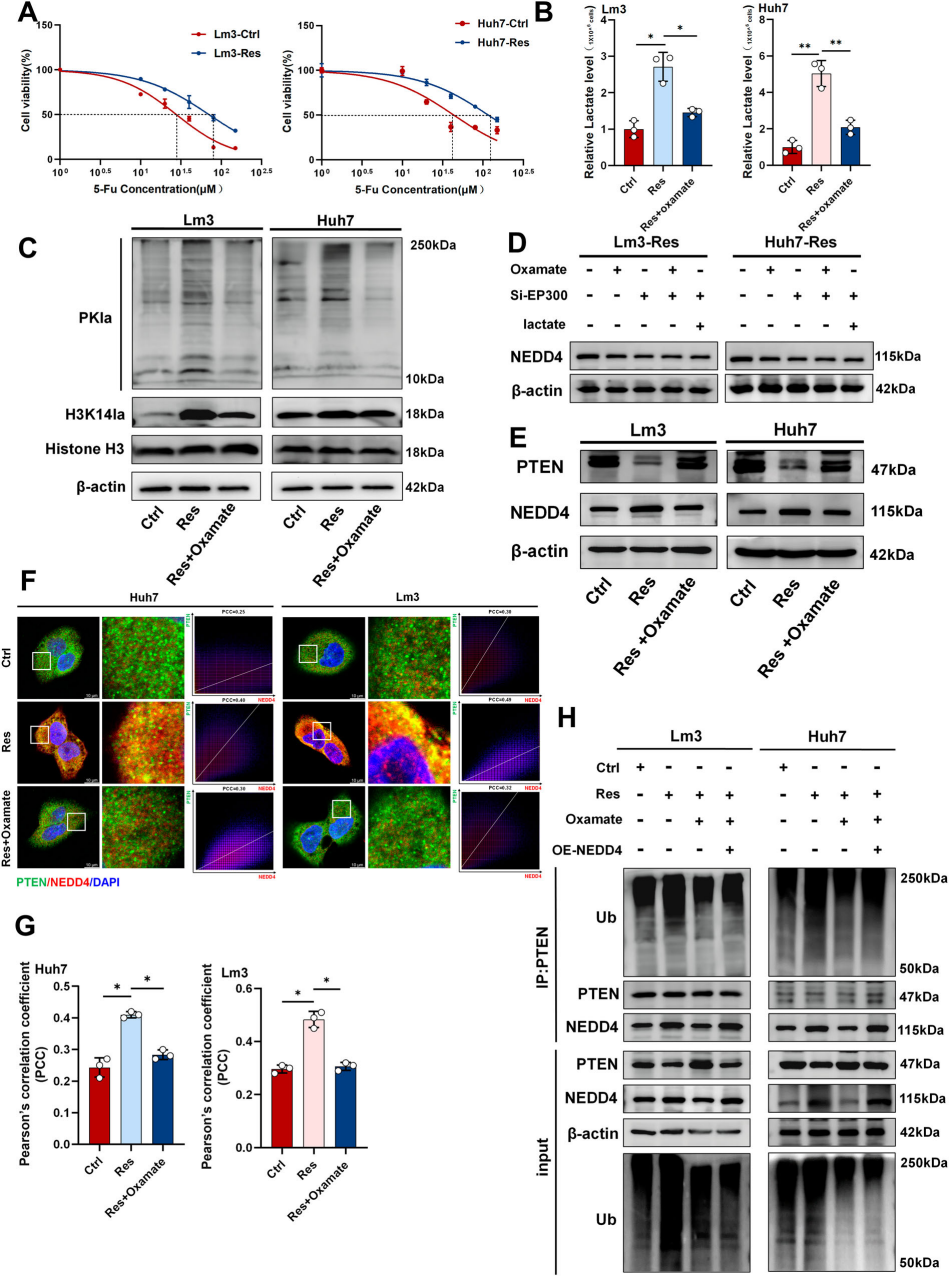
The histone lactylation/NEDD4/PTEN axis plays an important role in 5-Fu-resistant cells. **A** IC50 values of 5-Fu-sensitive (Ctrl) and 5-Fu-resistant strains (Res) in Lm3 and Huh7 cells. (The dashed line represents the drug concentration at which 50% of cells survive.) **B** Intracellular lactate production in 5-Fu-sensitive (Ctrl) and 5-Fu-resistant (Res) cells. (The dashed line represents the drug concentration at which 50% of cells survive.) **C** Western blot analysis of changes in PKla and H3K14la protein levels after oxamate treatment. **D** Changes in NEDD4 protein levels in 5-Fu-resistant cells (Res) under si-EP300, oxamate, and lactate treatments. **E** PTEN and NEDD4 protein levels were detected by western blotting. **F** Immunofluorescence confirmed the increased colocalization of PTEN and NEDD4 in 5-Fu-resistant cells, scale bar = 10 µm. The X-axis represents the pixel intensity of NEDD4 (red), and the Y-axis represents the pixel intensity of PTEN (green). **G** Colocalization was quantified using ImageJ (n = 3). **H** Detection of PTEN ubiquitination levels after Co-IP with PTEN in 5-Fu-sensitive (Ctrl) and resistant (Res) cells. *P < 0.05, **P < 0.01, ***P < 0.001, ****P < 0.0001.

Several recent studies have shown that transarterial infusion (TAI) chemotherapy with the FOLFOX regimen alone results in improved survival in advanced HCC patients [44, 45]. However, the effectiveness of FOLFOX combination chemotherapy is greatly limited by drug resistance. Although drug resistance is common in colorectal cancer, a similar phenomenon also exists in HCC [46, 47]. Therefore, we further explored whether there is a similar mechanism for resistance to 5-Fu. We constructed a 5-Fu resistant cell strain, in which the IC50 of Lm3-resistant cells increased from 27.64 μM to 70.09 μM in the sensitive strain, and the IC50 in the Huh7-resistant strain increased from 43.41 μM to 116.2 μM (Fig. 6A). The above results suggest that the 5-Fu resistant strain was successfully constructed. Similarly, active lactate production was also demonstrated in 5-Fu-resistant cell lines and could be inhibited by oxamate (Fig. 6B). Western blot analysis revealed that lactylation levels and H3K14la levels were significantly increased in 5-Fu-resistant cells (Fig. 6C). Similarly, we assessed global ubiquitination levels in 5-Fu-resistant cells, where the elevated ubiquitination observed in these resistant cells was effectively reduced by oxamate treatment (Supplementary Fig. S5.C). Changes in NEDD4 protein levels were detected using a lactylation inhibitor, si-EP300 and combination of two. The results showed that both lactylation inhibitors and si-EP300 downregulated NEDD4 protein levels, although the degree of downregulation was not statistically significant. Exogenous addition of lactate after si-EP300 transfection did not restore NEDD4 protein levels (Fig. 6D). We subsequently detected the protein levels of NEDD4 and PTEN, and found that the NEDD4 level was increased and the PTEN level was decreased in 5-Fu-resistant cell lines (Fig. 6E). Immunofluorescence indicated a strong increase in the colocalization of NEDD4 and PTEN in 5-Fu-resistant cell lines (Fig. 6F, G). Additionally, Co-IP experiments also demonstrated an increase in the binding of PTEN to NEDD4 in 5-Fu-resistant cell lines, along with an increase in the level of PTEN ubiquitination (Fig. 6H). To further confirm that NEDD4 mediates the ubiquitination and degradation of PTEN, we introduced Flag-NEDD4, Myc-PTEN, and HA-Ub into HEK293T cells for ubiquitination experiments. As expected, the introduction of NEDD4 significantly increased PTEN ubiquitination (Supplementary Fig. S6.A). These findings indicate that histone lactylation participated in the regulation of NEDD4-mediated ubiquitination and degradation of PTEN in HCC cells, playing a significant role in the development of resistance to 5-Fu.

In our study, we demonstrated that NEDD4 is highly expressed in cells resistant to 5-FU and OXA, and that OE-NEDD4 can partially reverse the inhibition of lactylation. However, it remains unclear whether HCC cells that naturally overexpress NEDD4 are inherently more resistant. Using the CCK-8 assay, we assessed changes in the IC50 values of OXA and 5-FU in both normal cells and cells overexpressing NEDD4. Overexpression of NEDD4 led to an increase in the IC50 of OXA from 6.096 μM to 8.883 μM in Lm3 cells and from 10.97 μM to 17.26 μM in Huh7 cells, as well as an increase in the IC50 of 5-FU from 27.44 μM to 37.75 μM in Lm3 cells and from 40.12 μM to 56.08 μM in Huh7 cells (Supplementary Fig. S6.B). Additionally, we analyzed the TCGA database to examine the relationship between NEDD4 expression and the IC50s of gemcitabine, cisplatin, sorafenib, and docetaxel. The results indicated that higher NEDD4 expression correlated with a decrease in the IC50 values of sorafenib, but an increase in the IC50 values of cisplatin and docetaxel. In addition, the IC50 change of gemcitabine was not significantly related to the expression of NEDD4 (Supplementary Fig. S6.C). In general, whether HCC that naturally overexpresses NEDD4 is prone to drug resistance appears to depend on the specific drug. However, this conclusion requires further validation through clinical data.

### Glycolysis/H3K14la/PTEN forms a positive feedback loop in OXA and 5-Fu resistant HCC

**Fig. 7:**
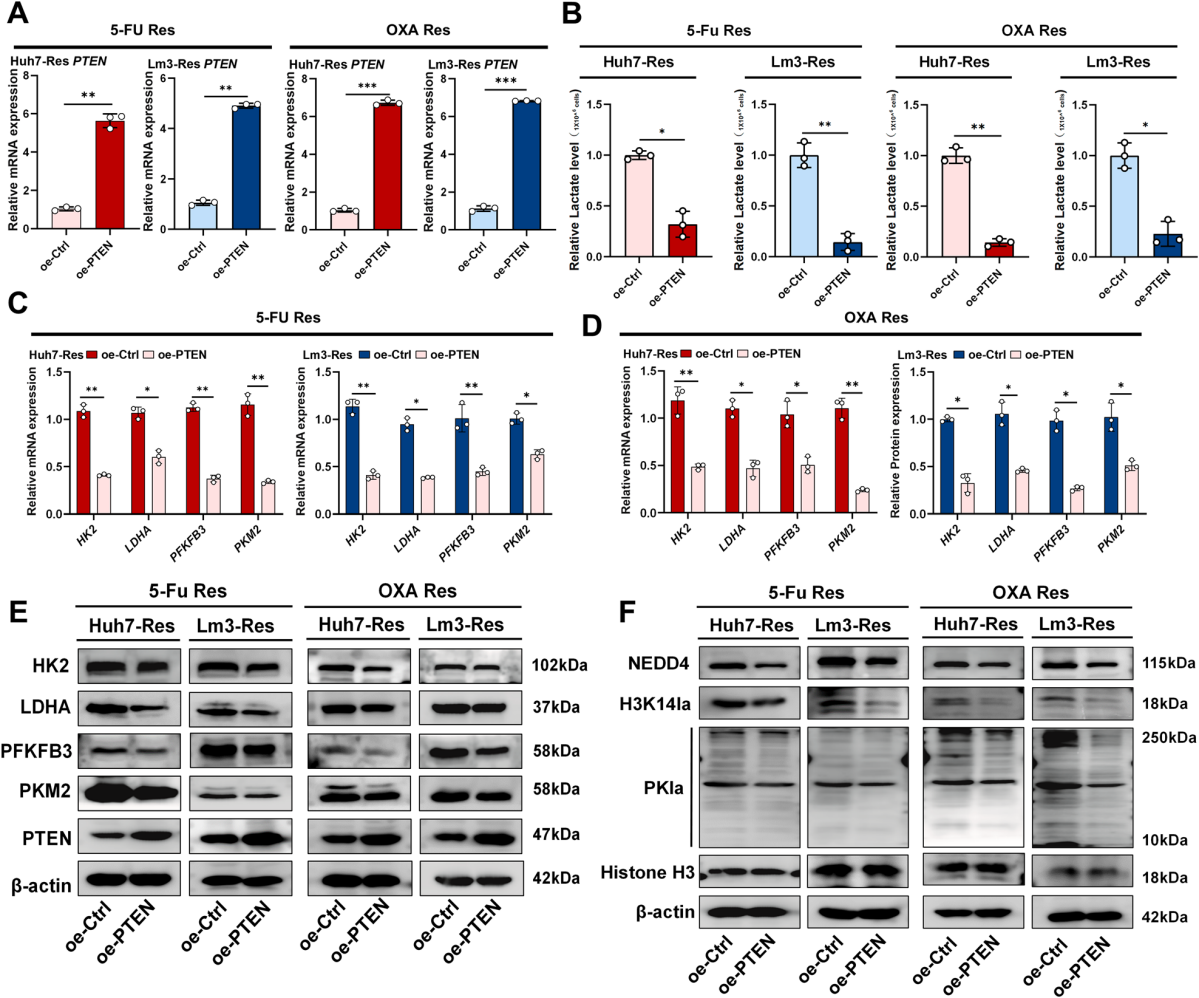
Overexpression of PTEN blocks the glycolysis/H3K14la/PTEN positive feedback loop in OXA and 5-Fu resistant HCC. **A** qRT‒PCR was used to verify the successful induction of PTEN overexpression of PTEN overexpression in OXA- and 5-Fu -resistant cell lines (Res) (n = 3). **B** Measurement of lactate levels in resistant cells overexpressing PTEN (n = 3). **C** qRT‒PCR confirmed altered levels of the glycolysis-related genes *PKM2, PFKFB3, LDHA* and *HK2* after OE-PTEN treatment in 5-Fu resistant cells (Res) (n = 3). **D** qRT‒PCR confirmed altered levels of the glycolysis-related genes *PKM2, PFKFB3, LDHA* and *HK2* after OE-PTEN treatment in OXA resistant cells (Res) (n = 3). **E** The protein levels of PKM2, PFKFB3, LDHA and HK2, which are key enzymes involved in the glycolytic process, were assayed after OE-PTEN treatment in resistant cells. **F** After OE-PTEN treatment, western blotting was used to detect PKla, H3K14la and NEDD4 protein levels in resistant cells. *P < 0.05, **P < 0.01, ***P < 0.001, ****P < 0.0001.

Previous studies have shown that PTEN plays an important role in regulating intracellular glycolysis [48, 49]. For example, *PKM2* transcription is regulated by PTEN/mTOR [50], and PTEN also reduces the stability of PFKFB3 [51]. To investigate whether PTEN also regulates glycolysis and influences H3K14la to establish a positive feedback loop in OXA and 5-Fu resistant cells, we overexpressed PTEN in OXA- and 5-Fu-resistant cells (OE-PTEN) (Fig. 7A) and found that PTEN overexpression significantly reduced the intracellular lactate levels (Fig. 7B). qRT‒PCR analysis revealed that the mRNA levels of glycolysis-related genes *PKM2*, *PFKFB3*, *LDHA*, and *HK2* decreased to varying degrees (Fig. 7C, D). Western blot assays showed that the protein levels of the key glycolytic enzymes PKM2, PFKFB3, LDHA and HK2 were significantly reduced (Fig. 7E). Importantly, the levels of PKla, H3K14la and NEDD4 also decreased dramatically (Fig. 7F). Overall, restoring PTEN levels in OXA- and 5-Fu-resistant cells significantly reduced glycolysis levels and disrupted the glycolysis/H3K14la/PTEN positive feedback loop.

### Histone lactylation regulates OXA resistance via NEDD4/PTEN in vivo

**Fig. 8:**
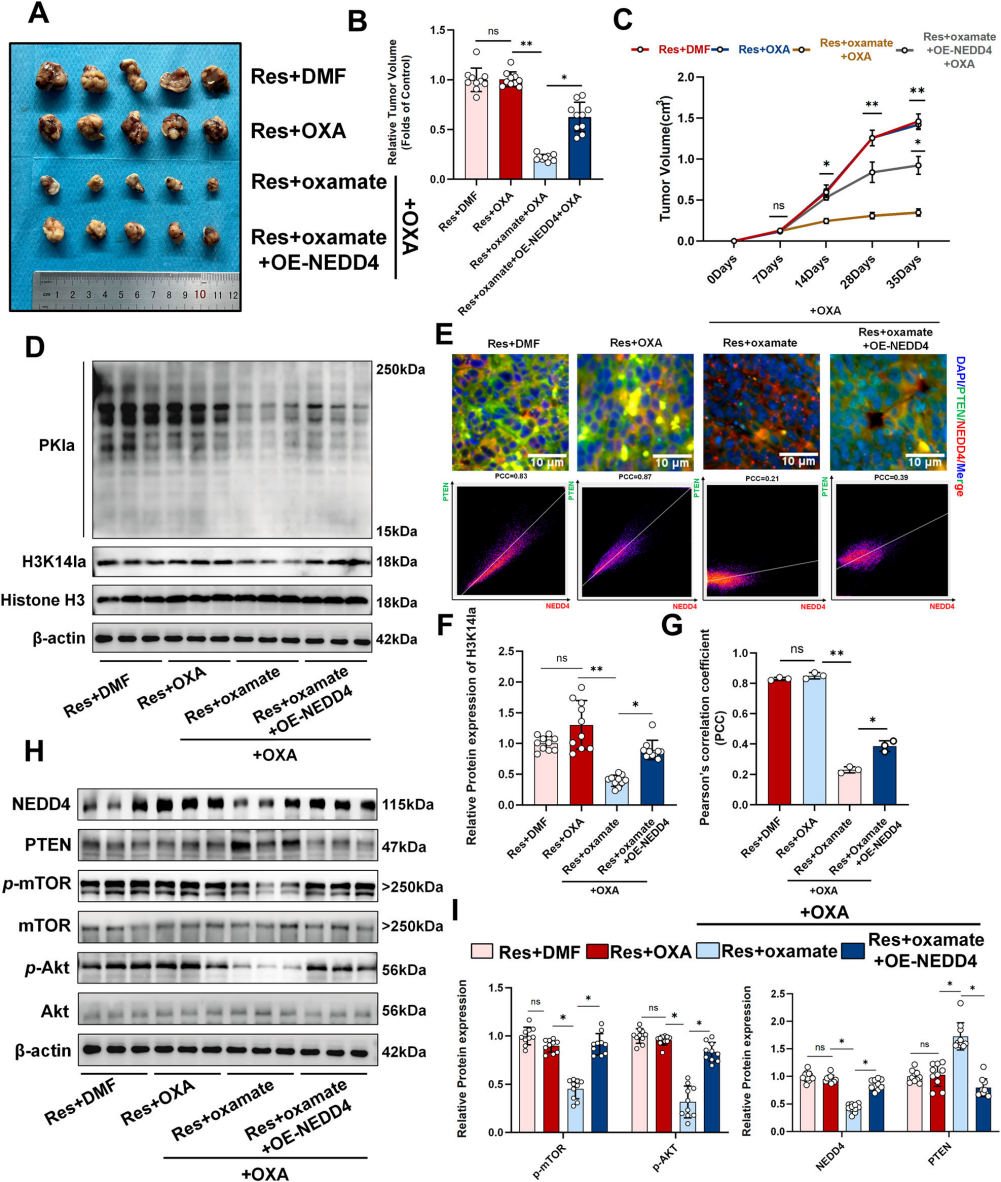
In vivo validation of the role of the histone lactylation/NEDD4/PTEN axis in OXA resistance. **A** Gross nude mouse xenograft (n = 5). **B** Quantification of tumor volume (n = 10). **C** Tumor volume changed over time (n = 10). **D** Western blot analysis of lactylation and histone lactylation levels in vivo. **E** Immunofluorescence detection and quantification of NEDD4 and PTEN colocalization in xenografts, scale bar = 10 µm. The X-axis represents the pixel intensity of NEDD4 (red), and the Y-axis represents the pixel intensity of PTEN (green). **F** Quantification of H3K14la protein levels (n = 10). **G** Quantification of Pearson’s correlation coefficient with immunofluorescence (n = 3). **H**, **I** Western blot analysis revealed changes in the levels of proteins, such as NEDD4, PTEN, and mTOR (n = 3). PCC Pearson’s correlation coefficient *P < 0.05, *P < 0.05, **P < 0.01, ***P < 0.001, ****P < 0.0001.

To investigate whether a similar mechanism of histone lactylation exists in vivo, OXA-resistant Lm3 cells were injected into male and female nude mice to establish a xenograft tumor model (Fig. 8A and Supplementary Fig. S7.A). Compared with OXA alone, the addition of the histone lactylation inhibitor oxamate significantly reduced the tumor volume. Conversely, the introduction of OE-NEDD4 increased the tumor volume accordingly (Fig. 8B, C). We subsequently detected changes in protein levels. The results suggested that OE-NEDD4 can significantly increase the levels of inhibited lactylation and histone lactylation (Fig. 8D, F and Supplementary Fig. S7.B). Immunofluorescence showed that NEDD4 colocalized with PTEN in vivo and was restored by NEDD4 overexpression after inhibition (Fig. 8E, G). Finally, similar results were also observed for the protein levels of NEDD4, PTEN, *p*-AKT, and *p*-mTOR in vivo (Fig. 8H, I and Supplementary Fig. S7.C, D). These results demonstrate that the histone lactylation/NEDD4/PTEN axis plays an important regulatory role in OXA resistance in vivo and this regulatory role is not significantly related to gender.

## Discussion

FOLFOX is widely recognized as an established treatment for HCC. Although combination therapy has been proven to improve therapeutic outcomes, the efficacy of FOLFOX is somewhat compromised by the emergence of drug resistance [52, 53]. In the present study, we generated HCC cell lines resistant to OXA and 5-Fu (the primary constituents of the FOLFOX regimen) and found that drug-resistant cells displayed enhanced glycolytic activity, which is consistent with previous investigations in this field [54–56]. However, unlike previous studies, we discovered that active glycolysis impacts tumor cell drug resistance by regulating signaling pathways, and it also demonstrated that the glycolysis product lactate is a substrate that enhances HCC drug resistance through histone lactylation [57, 58]. Mechanistically, our studies showed that histone lactylation enhanced NEDD4 expression and that the interaction of NEDD4 with PTEN promoted PTEN ubiquitination and degradation, thereby promoting glycolysis in a positive feedback loop and ultimately leading to the development of resistance to OXA and 5-Fu.

Although the mechanism of chemotherapy resistance in HCC has been extensively studied [59, 60], clinical practice often involves the use of multiple chemotherapeutic drugs in combination [61, 62]. Therefore, it is necessary to study the resistance mechanism of the combined use of multiple chemotherapeutic drugs. Our study revealed that histone lactylation plays an important regulatory role in OXA-resistant HCC cells. Interestingly, 5-Fu has a similar mechanism of action in drug-resistant cell lines, suggesting that histone lactylation may play a significant role in multidrug resistance of HCC. The use of inhibitors that target histone lactylation effectively sensitized cells to HCC chemotherapy, which may provide a novel approach for the clinical treatment of chemoresistant patients.

Metabolic reprogramming from oxidative phosphorylation to aerobic glycolysis, a phenomenon known as the “Warburg effect”, is a hallmark of cancer. Numerous studies have shown that glycolysis plays a regulatory role in tumor chemotherapy resistance by regulating intracellular redox, fat metabolism, and inducing cell death [8, 63, 64]. However, we found in this study that glycolysis plays a regulatory role through the lactylation modification of protein lysine sites by its metabolite lactate. Zhang and colleagues discovered a chemical modification called lactylation, which originally referred to the addition of lactyl groups to lysine amino acid residues in histone tails, and altered gene expression levels [9, 65]. Furthermore, histone lactylation has been extensively studied in various fields, including tumors [23], liver fibrosis [66], and myocardial infarction [67]. Since the discovery of histone lactylation, many genes regulated by it have been identified. In our study, we observed high levels of glycolysis and lactylation in OXA and 5-Fu resistant cells. In addition, we found that H3K14la played an important role in OXA and 5-Fu resistant HCC cells. Furthermore, based on ChIP-seq analysis, H3K14la was found to bind to the NEDD4 promoter region. Using a histone lactylation inhibitor and a silencing transferase (si-EP300), we subsequently demonstrated that NEDD4 is regulated by histone lactylation. To the best of our knowledge, this study is the first to report that NEDD4 is regulated by histone lactylation.

As an E3 ligase, NEDD4 is highly expressed in breast cancer [68], pancreatic cancer [30], and gastric cancer [69]. It is associated with poor survival in cancer patients by regulating tumor cell proliferation, apoptosis and stemness [13]. NEDD4 has also been demonstrated to be involved in the resistance of tumor cells, including gastric cancer, glioblastoma and skin cancer cells, to chemotherapy [70]. In this study, we also found that NEDD4 plays a cancer-promoting role in OXA-resistant cells by silencing NEDD4 (si-NEDD4), which is consistent with the findings of previous studies. Our study found that NEDD4 mediated the ubiquitination and degradation of PTEN by binding to PTEN, which activated the downstream PI3K/Akt/mTOR signaling pathway, thus promoting OXA and 5-Fu resistance in tumor cells. Previous studies have confirmed that NEDD4 is involved in the ubiquitination and degradation of the large tumor suppressor kinase 1 (LATS1) in tumor cells, thereby affecting the activity of the Hippo pathway [13]. Additionally, NEDD4 plays a role in regulating the protein expression of guanylyl cyclase domain-containing 1 (GUCD1), insulin receptor substrate 2 (IRS-2), and other related proteins, contributing to its biological regulatory mechanisms [71]. However, whether these mechanisms are also active in OXA- and 5-Fu-resistant HCC remains unexplained in this study. Interestingly, our study also found that the overexpression of PTEN in drug-resistant cells effectively reduced the levels of intracellular glycolysis, PKla, H3K14la, and NEDD4 levels, suggesting that glycolysis/H3K14la/PTEN positive feedback loop may exist in OXA- and 5-Fu- resistant cells. Restoring PTEN levels in drug-resistant cells may prove to be an effective therapeutic strategy. Although previous studies have shown that PTEN may affect glycolysis by regulating PKM2 and PFKFB3 [72], this study did not investigate the specific molecular mechanism by which PTEN affects glycolysis. In conclusion, our study found that NEDD4 mediated PTEN ubiquitination in OXA and 5-Fu resistant HCC cells, thereby promoting active intracellular glycolysis to form a positive feedback loop. In addition, the degradation of PTEN also activated the PI3K/Akt/mTOR pathway, thus promoting the malignant behavior of cells. These findings elucidate potential targets for HCC drug resistance and provide an alternative mechanism for regulating PTEN that differs from previous studies.

This study has certain limitations. First, there is a lack of support from clinical samples and supplementation in clinical trials. We studied OXA and 5-Fu resistant cell lines, but we were unable to construct FOLFOX-resistant HCC cell lines. In addition, we did not use commercially available multi-validated resistant strains due to the limitations of our conditions. Biological effects of the metabolic function of lactate cannot be completely ruled out, so whether lactylation and the metabolic effects of lactate coexist needs further study. In addition, whether there are other mechanisms of action in OXA- and 5-Fu-resistant cells, whether other targets of NEDD4 play a role, and the causal relationship between NEDD4 and lactylation modification in resistant cells remain to be further studied. Finally, further investigation is needed to elucidate the mechanism by which H3K14la regulates NEDD4 and its binding site on the promoter.

Overall, our results demonstrate that histone lactylation leads to the upregulation of NEDD4 expression and facilitates PTEN ubiquitination and degradation. This process subsequently activates the PI3K/Akt/mTOR signaling pathway, promoting multidrug resistance in HCC. Fully clarifying the molecular mechanisms of HCC resistance to combined chemotherapy and chemotherapy resensitization represents a new therapeutic strategy for treating HCC.

## Conclusions

**Fig. 9:**
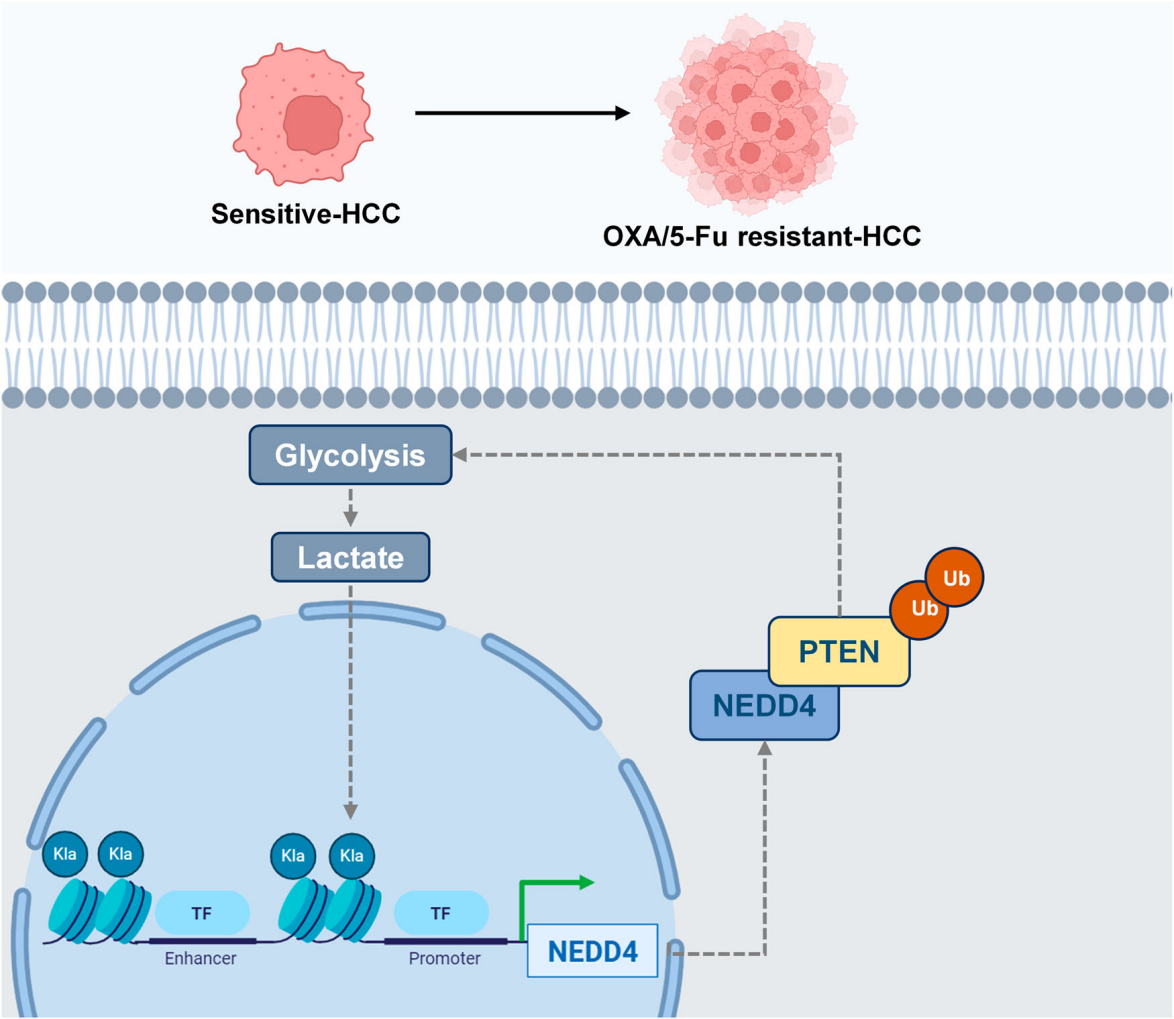
Illustration of the molecular mechanisms in OXA- and 5-Fu-resistant HCC.

Our study revealed that the level of intracellular lactylation, which plays a regulatory role in the form of histone lactylation, was increased in OXA- and 5-Fu-resistant HCC cells. H3K14la regulated NEDD4 expression and further promoted the ubiquitination and degradation of PTEN. Reduced PTEN activated the PI3K/Akt/mTOR signalling pathway and induced chemotherapy resistance in HCC. Furthermore, PTEN degradation alleviated the inhibition of intracellular glycolysis, thereby increasing the activity of intracellular glycolysis and establishing a positive feedback loop. Inhibiting intracellular histone lactylation could effectively inhibits the malignant progression of OXA and 5-Fu resistant cells and increases the sensitivity of HCC cells to chemotherapy (Fig. 9).

## Supplementary information

Supplementary Figure

Supplementary Methods

Supplementary Table

un-cropped images

## Data Availability

The datasets generated during and/or analyzed during the current study are available from the corresponding author upon reasonable request. The full-length uncropped original Western blots were uploaded as “original Western blots”.
