# Supplementary material for: Histone lactylation promotes multidrug resistance in hepatocellular carcinoma by forming a positive feedback loop with PTEN
Source: Cell Death Dis. 2025 Jan 31;16(1):59. doi: 10.1038/s41419-025-07359-9 (PMC11785747; doi:10.1038/s41419-025-07359-9)
Supplement: Supplementary file 1 — Supplementary Figure [file 41419_2025_7359_MOESM1_ESM.docx]

**Supplementary Figure:**

**
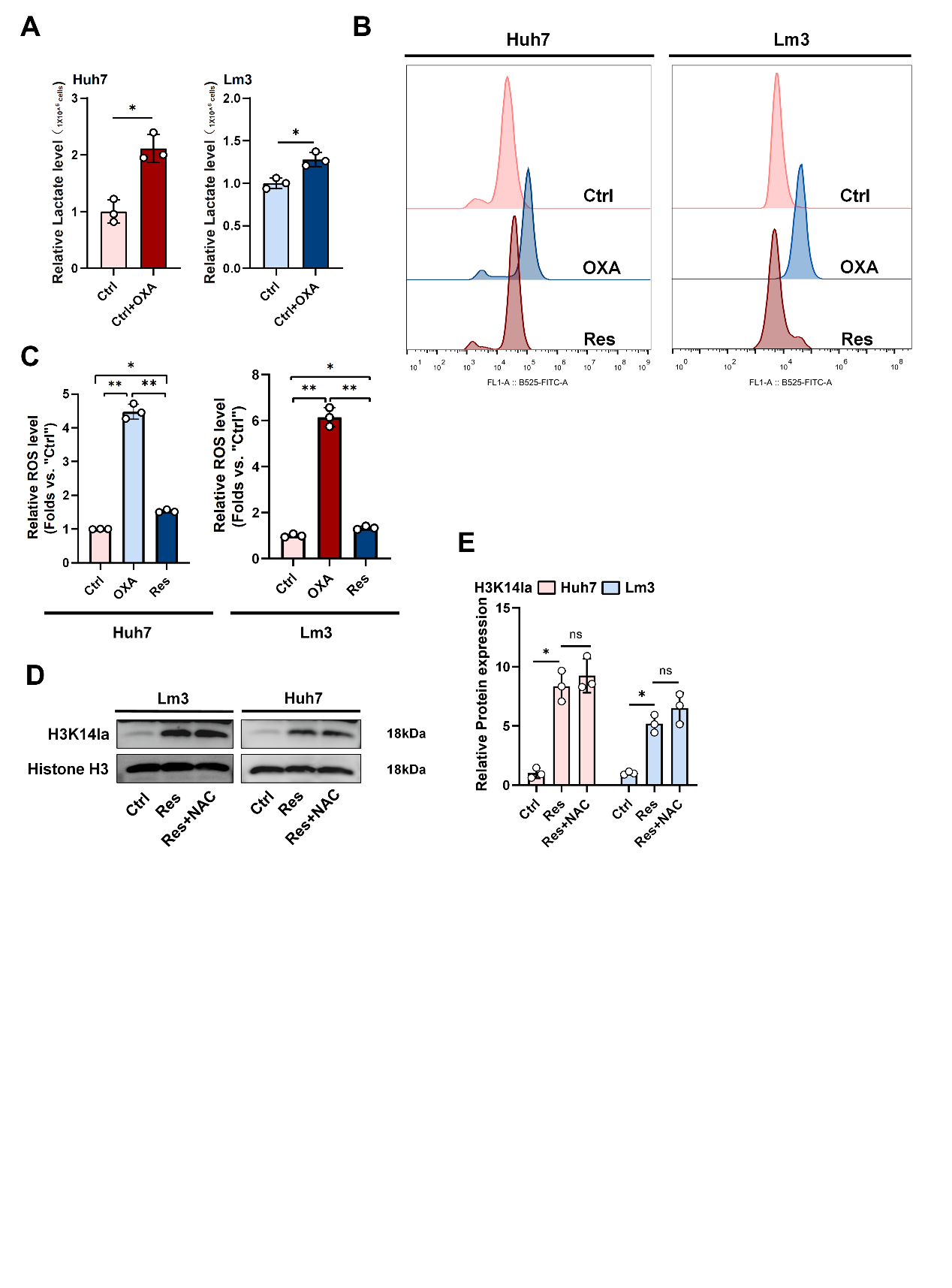
**

**S1. ROS accumulation in drug-resistant cells does not affect histone lactylation.** (A) Lactate levels in sensitive cells (Ctrl), both untreated and treated with OXA, were measured. (B-C) Flow cytometry was used to detect and quantify ROS accumulation in sensitive strains, OXA-treated sensitive strains, and resistant strains. (n=3). (D-E) Western blotting was used to detect and quantify H3K14la protein expression after treatment with the ROS scavenger NAC (n=3). *P < 0.05, *P < 0.05, **P < 0.01, ***P < 0.001, ****P < 0.0001.


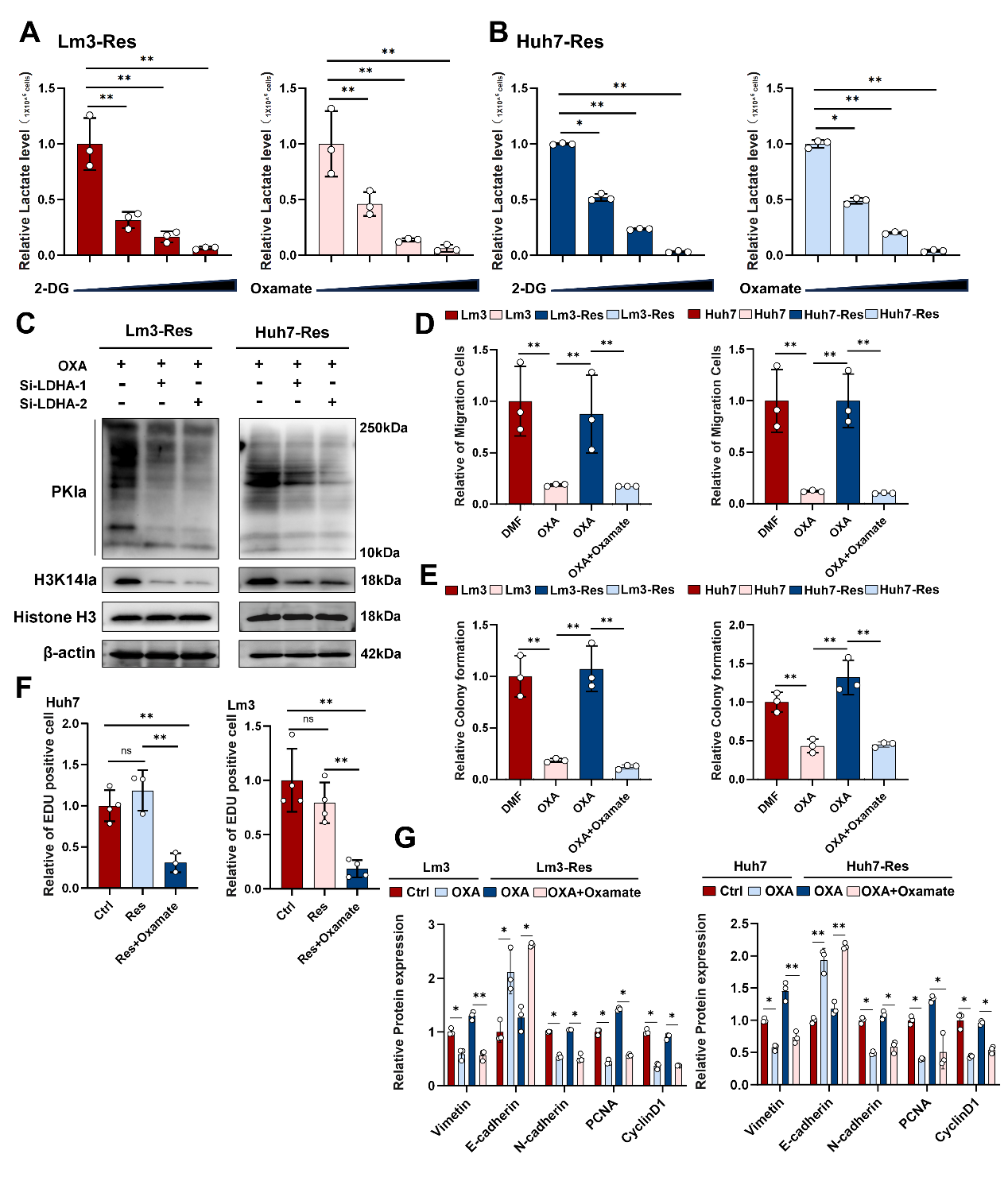


**S2.** **Inhibiting histone lactylation inhibits OXA resistance.** (A-B) Lactate production in OXA-resistant Huh7 (Huh7-Res) cells after the administration of 2-DG or oxamate. (C) Western blot analysis of PKla and H3K14la expression levels after si-LDHA and si-LDHB treatments. (D) Quantification of OXA-sensitive (Ctrl) and OXA-resistant (Res) cells in the Transwell system (n=3). (E) Quantification of colony formation in OXA-sensitive (Ctrl) and OXA-resistant (Res) cells (n=3). (F) Quantification of EdU in OXA-sensitive (Ctrl) and OXA-resistant (Res) cells (n=3). (G) Quantitative western blots in OXA-sensitive (Ctrl) and OXA-resistant (Res) cells (n=3). *P < 0.05, *P < 0.05, **P < 0.01, ***P < 0.001, ****P < 0.0001.


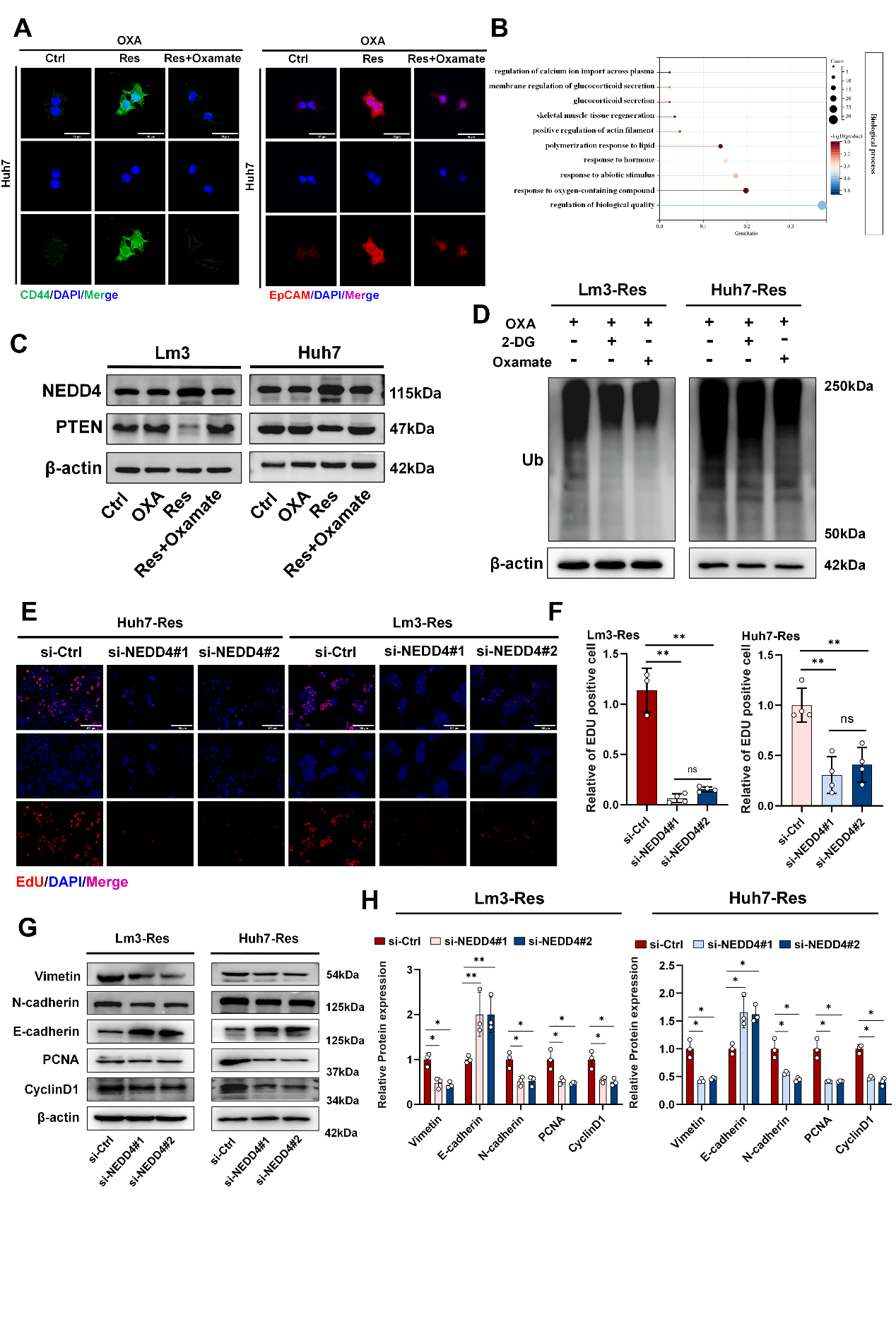


**S3. NEDD4 mediates the malignant behavior of OXA-resistant cells.** (A) Both sensitive and resistant Huh7 cells were treated with OXA and immunofluorescence was used to detect changes in the protein levels of the stemness-related proteins CD44 and EpCAM, scale bar = 10 µm. (B) GO analysis of ChIP-Seq data. (C) Western blotting was used to detect the expression levels of NEDD4 and PTEN proteins in sensitive strains (Ctrl), OXA-treated sensitive strains, resistant strains (Res) and oxamate-treated resistant strains. (D) Detection of total ubiquitination levels in OXA-resistant cells. (E-F) EdU was used to assess and quantify cell proliferation after NEDD4 was silenced with si-NEDD4 in OXA-resistant (Res) cells (n=3), scale bar = 100 µm. (G-H) Western blot analysis of changes in the levels of EMT-related proteins and proliferation-related proteins after si-NEDD4 transfection (n=3). *P < 0.05, *P < 0.05, **P < 0.01, ***P < 0.001, ****P < 0.0001.


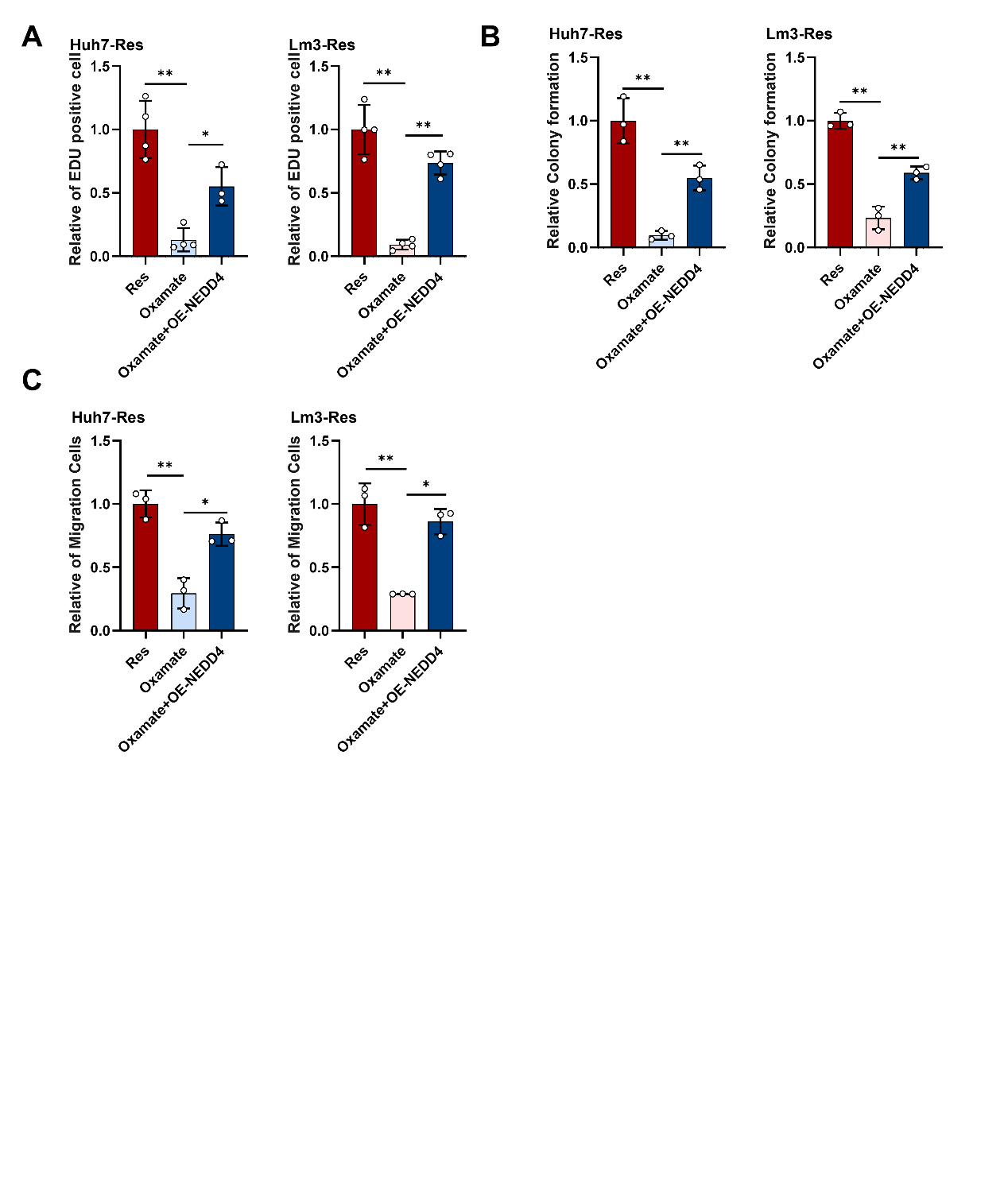


**S4. Quantification of cell proliferation and migration experiments.** (A) Quantification of EdU assays after OXA-resistant cells (Res) were treated with low-dose OXA, and then treated with oxamate or OE-NEDD4 (n=3). (B) Quantification of colony formation experiments after OXA-resistant cells (Res) were treated with low-dose OXA, and then treated with oxamate or OE-NEDD4 (n=3). (C) Quantification of Transwell assays after OXA-resistant cells (Res) were treated with low-dose OXA, and then treated with oxamate and OE-NEDD4 respectively (n=3). *P < 0.05, *P < 0.05, **P < 0.01, ***P < 0.001, ****P < 0.0001.


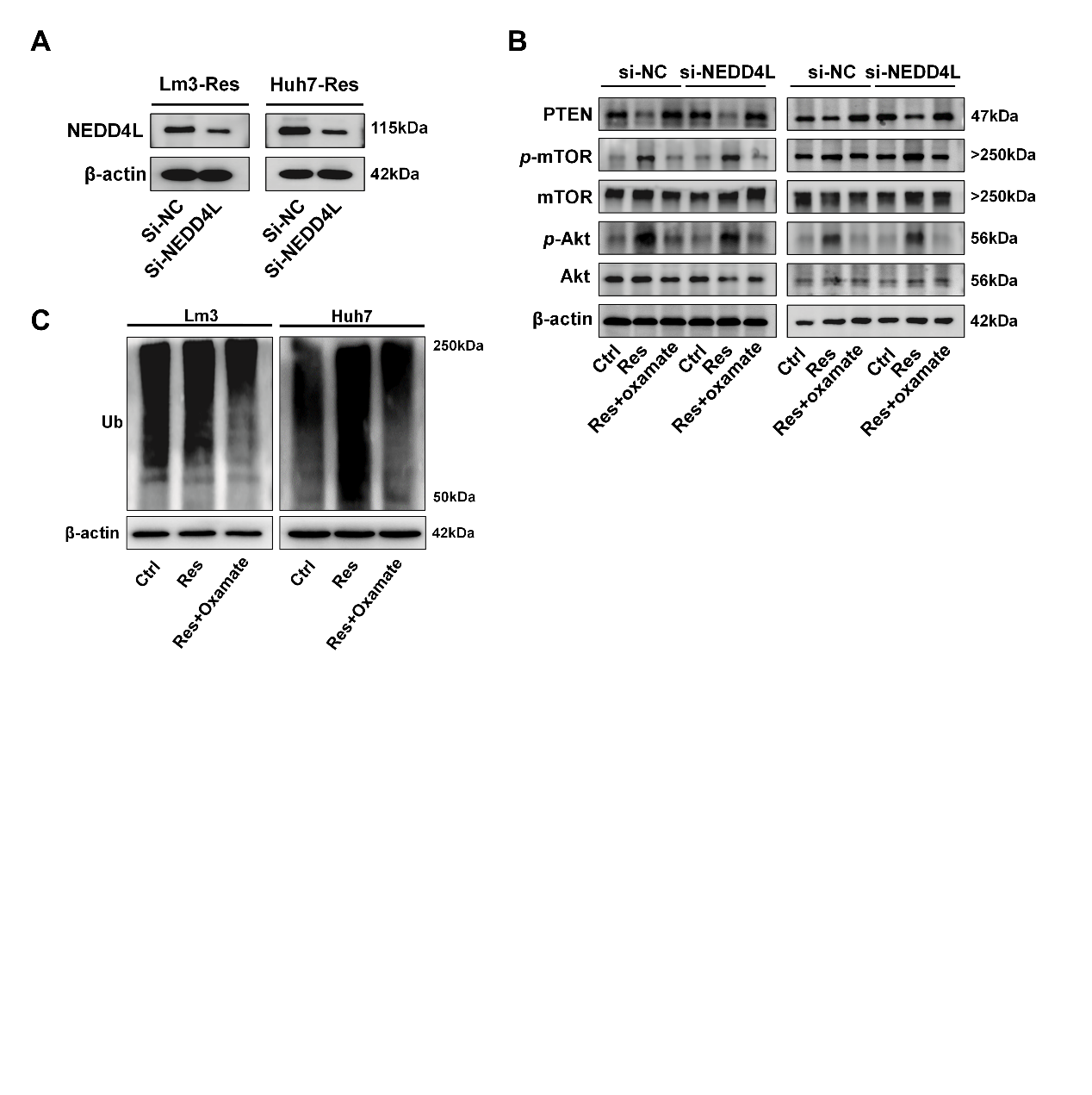


**S5. Histone lactylation exerts its biological effects through NEDD4 rather than NEDD4L.** (A) Western blot analysis verified the silencing efficiency of si-RNAs in drug-resistant cell lines. (B) After silencing NEDD4L, changes in PTEN and downstream pathways were observed. (C) Detection of the overall intracellular ubiquitination level in 5-Fu-resistant cells. *P < 0.05, *P < 0.05, **P < 0.01, ***P < 0.001, ****P < 0.0001.


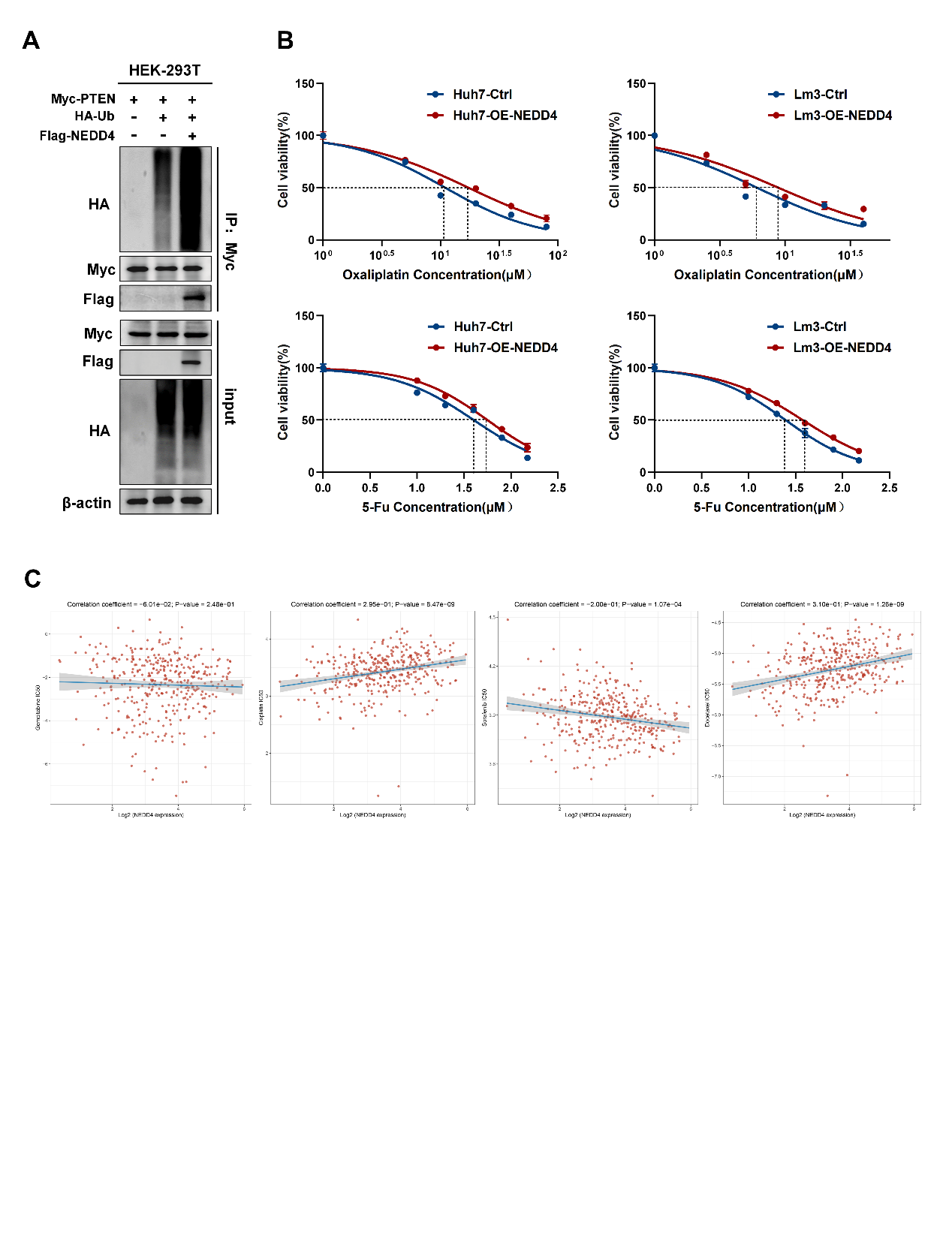


**S6. NEDD4 mediates the ubiquitination of PTEN.**

(A) Ubiquitination levels were detected after Myc-PTEN, HA-Ub, and Flag-NEDD4 were transferred into HEK-293T cells. (B) Changes in OXA and 5-Fu IC50 values in Lm3 and Huh7 cells after OE-NEDD4 was detected with CCK8 kit (n=3). (C) The correlations between NEDD4 expression and gemcitabine, sorafenib, cisplatin and docetaxel IC50 values was analysed in the TCGA database. *P < 0.05, *P < 0.05, **P < 0.01, ***P < 0.001, ****P < 0.0001.


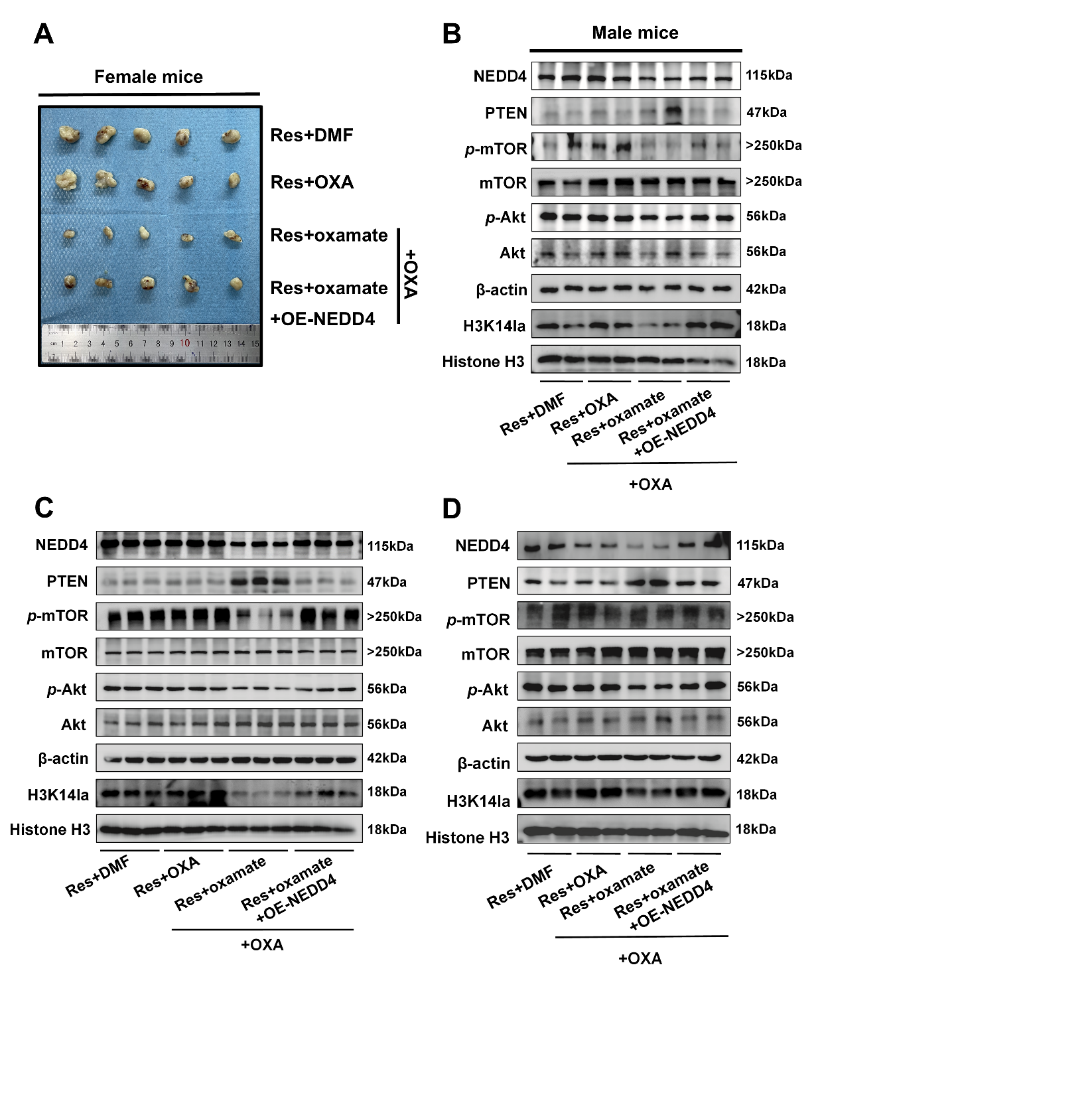


**S7. Validation of the H3K14la/NEDD4/PTEN axis in female mice.** (A) Gross image of tumor in female mice (n=5). (B) Western blotting was used to detect the changes in H3K14la/NEDD4/PTEN protein levels in male mice (n=2). (C-D) Western blot detection and quantification of H3K14la/NEDD4/PTEN protein levels in tumor tissues of female mice (n=5).
