# Supplementary material for: Histone lactylation promotes multidrug resistance in hepatocellular carcinoma by forming a positive feedback loop with PTEN
Source: Cell Death Dis. 2025 Jan 31;16(1):59. doi: 10.1038/s41419-025-07359-9 (PMC11785747; doi:10.1038/s41419-025-07359-9)
Supplement: Supplementary file 2 — Supplementary Methods [file 41419_2025_7359_MOESM2_ESM.docx]

**Supplementary methods**

*Spheroid formation assay*

A total of 5000 cells/mL were resuspended in spheroid formation medium (TSCM medium, QIDABIO, China) and seeded in ultralow attachment 12-well plates (1000 cells/well, Corning, USA). After 10 d of culture, the size of the cancer spheres was observed under an inverted microscope by randomly selecting Ten fields of view. A total of three biological replicates were performed for quantitative analysis.

*EdU proliferation assay*

In accordance with our previous study(1), cell proliferation was assessed using a Cell-Light 5-ethynyl-2-deoxyuridine (EdU) Apollo567 in vitro kit (Beyotime, #C0078S, China) according to the manufacturer's instructions. Briefly, cells (2 × 10⁴ per well) were cultured on 12-well glass slides (Biosharp, China), incubated with 50 μM EdU (1:1000) for 6-h, fixed with 4% formaldehyde for 20 min, and permeabilized with 0.5% Triton X-100. Next, 100 μL of Apollo® reaction mixture was added to each well and incubated for 30 min under light-shielded conditions. After three washes with PBS,the cell nuclei were counterstained with DAPI (Biosharp, #BL520B, China) for 20 min. EdU-labelled cells were observed by fluorescence microscopy (Leica, DM6 B, 40× objective) and normalized to the total number of DAPI-stained cells. Ten fields were randomly selected for quantitative analysis in each sample. A total of three biological replicates were performed for quantitative analysis.

*Colony formation assay*

In accordance with our previous studies(1), cells were seeded at a density of 600 cells/well in 6-well plates, cultured in 6 μM OXA and subjected to different treatments for 7 days, fixed with 4% paraformaldehyde, stained with crystal violet (Biosharp, #BL802A, China) for 20 min, rinsed with running water, and photographed after drying. Each experiment was performed with three biological replicates, and each complete area was photographed and quantified.

*Cell transfection*

Cells in the logarithmic growth phase were seeded in 6-well plates (6 × 10 5 cells/well) and cultured for 24 h. siRNA (20 nM), Lipofectamine 3000 (Invitrogen, #L3000015, USA), and Opti-MEM low serum medium (Gibco, #319085062, USA) were added according to the manufacturer's instructions, and the cells were cultured for another 24 h before subsequent experiments. siRNAs were designed and synthesized by RiboBio (Guangzhou, China). The plasmid loaded with NEDD4 cDNA was constructed and packaged into lentivirus by Wuxi Puhe Biotechnology Co., Ltd. The lentiviral particles were added to polybrene medium and cultured for 24 h. The cells were selected with 2.5 μg/ml puromycin according to the manufacturer's instructions.

*CHX and MG132 treatment*

Cycloheximide (CHX) pulse-chase experiments were performed to measure protein half-lives. After the indicated treatment, the cells were evenly distributed in 6-well plates. Huh7 cells, Lm3 cells, and resistant strains (1 × 10⁵ cells each) were treated with 20 μg/mL CHX (Sigma, #C7698). The cells were harvested at 0, 2, 4 and 6 h post-treatment and subjected to western blot analysis. Similarly, the same cells were treated with 10 μM MG132 (Sigma, #M8699), harvested at 0, 2, 4 and 6 h post treatment, and then analysed by Western blot.

*Coimmunoprecipitation (Co-IP)*

IgG and the indicated antibodies were used for Co-IP according to the manufacturer's instructions. Briefly, cell lysates were preincubated with antibodies on a rotator at 4°C for 2 h. Protein A/G plus agarose (Santa Cruz, USA) was then added to the samples, which were subsequently incubated overnight at 4 °C. The complexes were washed three times with immunoprecipitation assay lysis buffer supplemented with a protease inhibitor cocktail, and then boiled in 2 × SDS‒PAGE loading buffer for 10 min before detection.

*Ubiquitination assay*

To analyze the ubiquitination of PTEN in vivo, cells were co-transfected with Myc-PTEN, HA-Ub, and Flag-NEDD4, treated with MG132 for 6 h, and lysed in NP-40 lysis buffer (50 mM Tris-HCl, pH 7.4; 150 mM NaCl; 1% NP-40) containing 0.1 mM phenylmethylsulfonyl fluoride (PMSF), 2 mM aprotinin, and 10 mM N-ethylmaleimide (NEM). High-quality Myc antibodies pre-coupled to Protein A/G agarose were added to the protein lysate and incubated with rotation overnight at 4°C. Finally, the beads were washed five times with lysis buffer, ubiquitinated PTEN was separated on a 10% SDS-PAGE gel, and detected by Western blotting using anti-HA antibodies.

*TCGA database analysis*

The online website module (https://www.home-for-researchers.com/#/) was used to analyze the correlation between NEDD4 expression and prognosis in different tumor tissues in the TCGA database. The relationship between NEDD4 expression and gemcitabine, cisplatin, sorafenib, and docetaxel IC50 in HCC patients in the TCGA database (n=371) was further analyzed. GO and KEGG were completed with the help of an online platform (http://www.sangerbox.com/).

**References：**

1. Zeng Y, Jiang H, Zhang X, Xu J, Wu X, Xu Q, et al. Canagliflozin reduces chemoresistance in hepatocellular carcinoma through PKM2-c-Myc complex-mediated glutamine starvation. Free Radic Biol Med. 2023;208:571-86.
