# Supplementary material for: Histone lactylation promotes multidrug resistance in hepatocellular carcinoma by forming a positive feedback loop with PTEN
Source: Cell Death Dis. 2025 Jan 31;16(1):59. doi: 10.1038/s41419-025-07359-9 (PMC11785747; doi:10.1038/s41419-025-07359-9)
Supplement: Supplementary file 3 — Supplementary Table [file 41419_2025_7359_MOESM3_ESM.docx]

**Supplementary Table**

**Table1. Primer sequences**

| Gene |  | Sequence |
| --- | --- | --- |
| *HK-II* | F | TGGAACTGGTGGAAGGAGAAGAGG |
|  | R | AATTCTGTGCGGAAGTCATCTAGGC |
| *PKM* | F | TGCCGCCTGGACATTGATTCAC |
|  | R | AGTTCAGACGAGCCACATTCATTCC |
| *PTEN* | F | TGGATTCGACTTAGACTTGACCT |
|  | R | GGTGGGTTATGGTCTTCAAAAGG |
| *LDHA* | F | AGATTCCAGTGTGCCTGTATG |
|  | R | ACCTCTTTCCACTGTTCCTTATC |
| *PFK* | F | ATCGTCGACAGCATCGAATC |
|  | R | CGACGACGCTCACCTTTAAT |

**Table2.** **Antibody manufacturer and product number were used in this study**

| Gene | Merchant | Cas |
| --- | --- | --- |
| PKM2 | Proteintech | 15822-1-AP |
| PCNA | Proteintech | 10205-2-AP |
| E-cadherin | Proteintech | 20874-1-AP |
| Vimentin | Proteintech | 10366-1-AP |
| N-cadherin | Proteintech | 22018-1-AP |
| H3K14la | PTMBIO | PTM-1414RM |
| H3K14la | PTMBIO | PTM-1426RM |
| PKla | PTMBIO | PTM-1401 |
| Histone-H3 | Proteintech | 17168-1-AP |
| Cyclin D1 | Proteintech | 26939-1-AP |
| NEDD4 | Proteintech | 21698-1-AP |
| NEDD4L | Proteintech | 13690-1-AP |
| HK-II | Proteintech | 22029-1-AP |
| LDHA | Proteintech | 19987-1-AP |
| AKT | Proteintech | 60203-2-Ig |
| *p*-AKT | Proteintech | 80455-1-RR |
| mTOR | Proteintech | 28273-1-AP |
| *p*-mTOR | Abcam | ab109268 |
| Ubiquitin | Proteintech | 10201-2-AP |
| PTEN | Affnity | #AF5447 |
| PFKFB3 | Proteintech | 13763-1-AP |
