# Supplementary material for: Histone lactylation promotes multidrug resistance in hepatocellular carcinoma by forming a positive feedback loop with PTEN
Source: Cell Death Dis. 2025 Jan 31;16(1):59. doi: 10.1038/s41419-025-07359-9 (PMC11785747; doi:10.1038/s41419-025-07359-9)
Supplement: Supplementary file 4 — un-cropped images [file 41419_2025_7359_MOESM4_ESM.pdf]

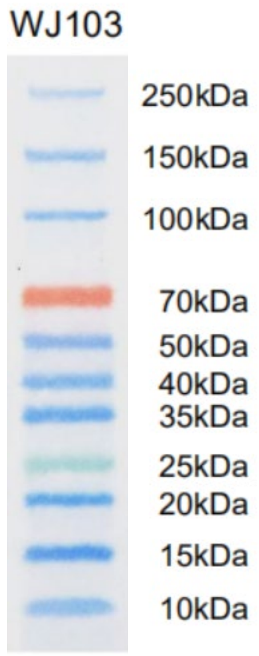

The markers were purchased from Yazyme Biotechnology Co., Ltd. (WJ101, China), and their molecular weights are indicated in the figure. For our experiments, we utilize 10% or 12.5% SDS-PAGE gels. After transferring to a PVDF membrane (ISEQ00010, Merck KGaA, Darmstadt, Germany), we cut out the respective membrane segment based on the target protein's molecular weight and the intervals as indicated by the marker. Subsequently, the corresponding primary antibody for the target protein is incubated. Finally, the development of the western blot is carried out using ProteinSimple FluorChemE, eBlot Touch Imager, and Bio-Rad ChemiDoc western blot imaging system.

Figure.1E

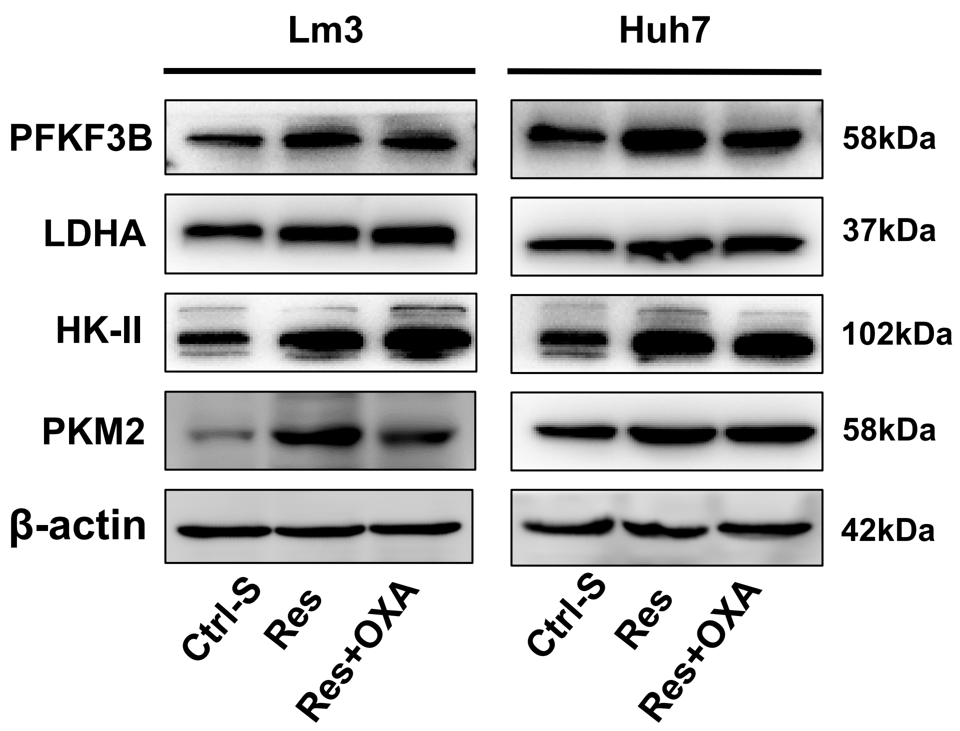
Lm3
Huh7
PFKF3B
LDHA
HK-II
PKM2
β-actin
58kDa
37kDa
102kDa
58kDa
42kDa
Ctrl-S
Res
Res+OXA
Ctrl-S
Res
Res+OXA

Lm3

PFKF3B 58kDa

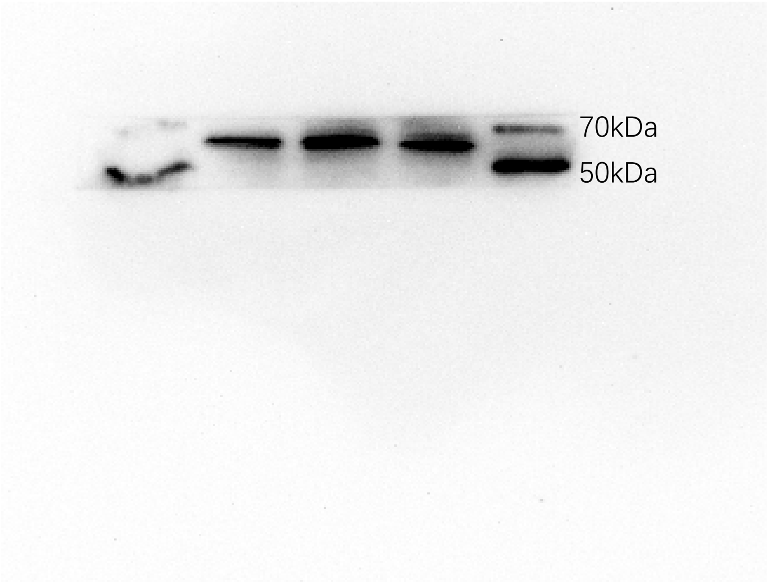

LDHA 37kDa

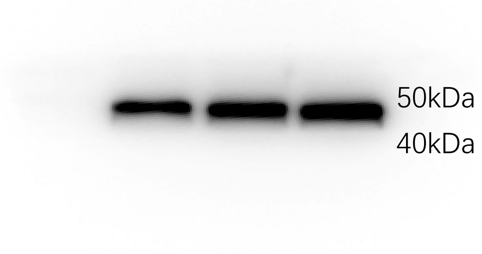

HK-II 102kDa

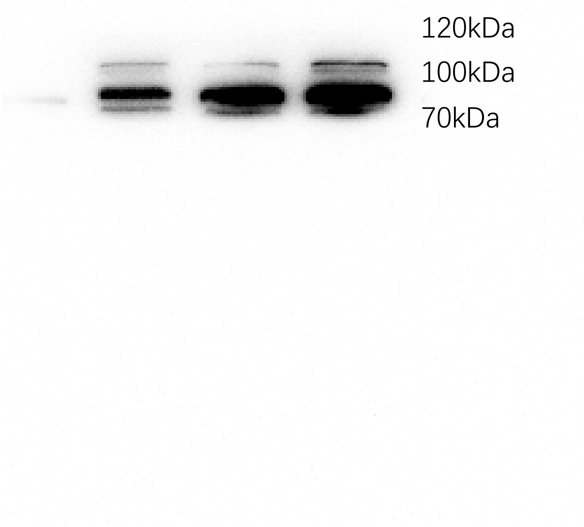

PKM2 58kDa

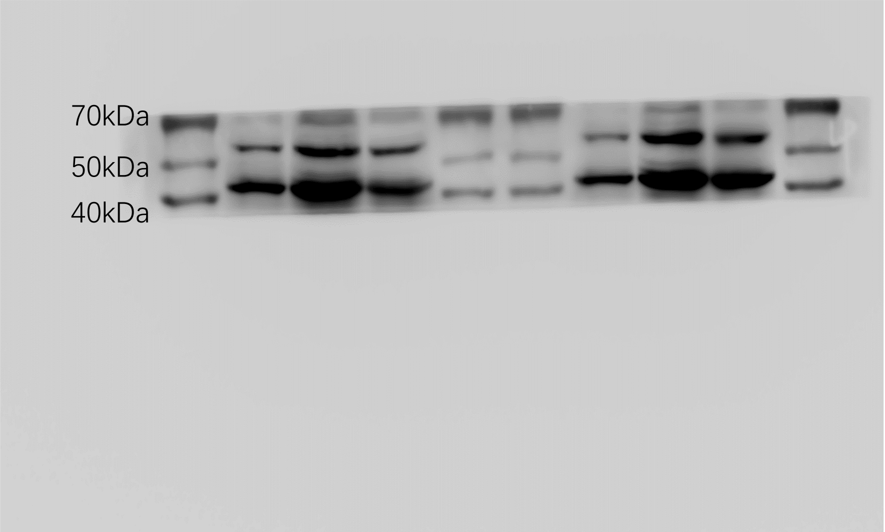

β-actin 42kDa

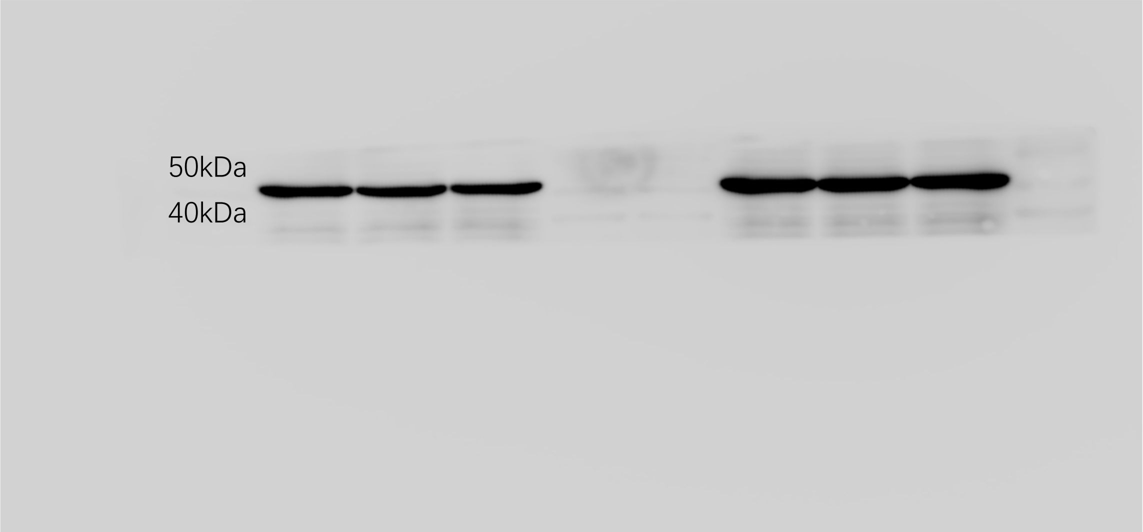

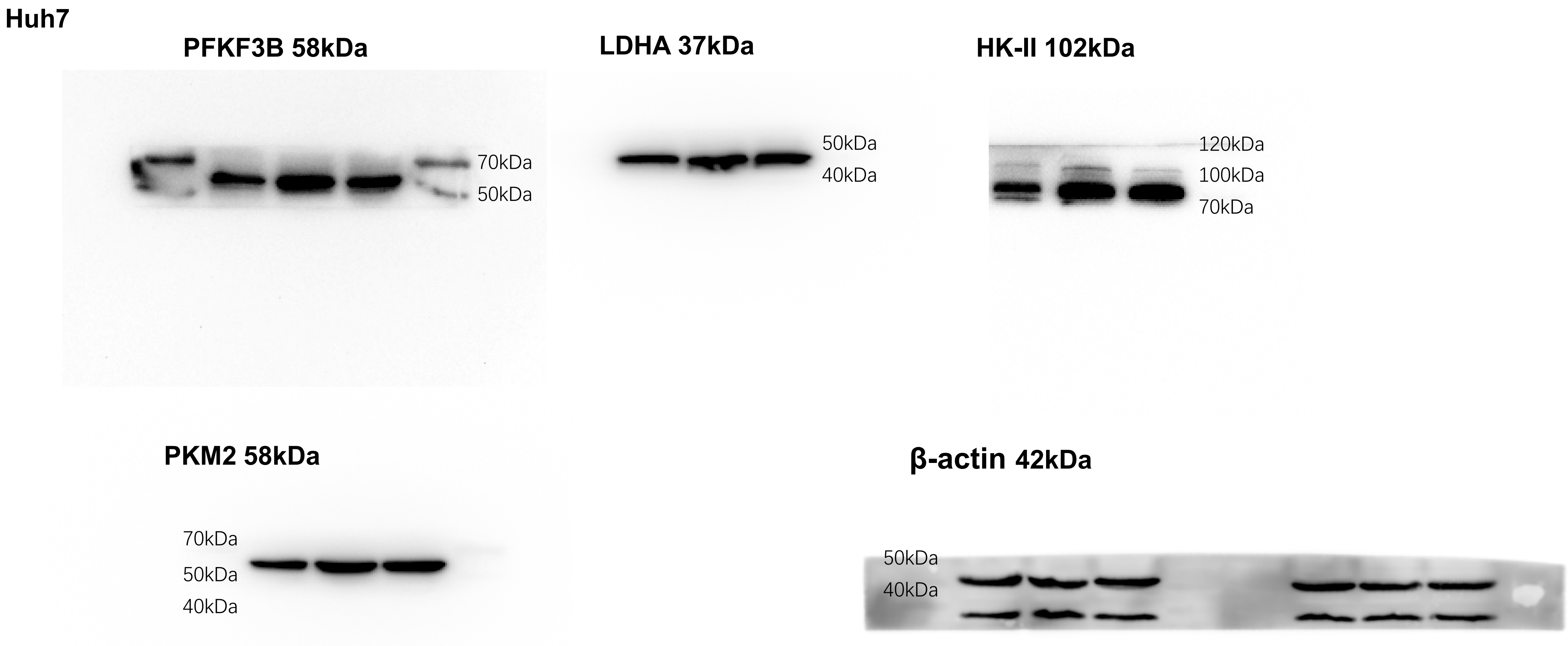
Huh7
PFKF3B 58kDa
70kDa
50kDa
LDHA 37kDa
50kDa
40kDa
HK-II 102kDa
120kDa
100kDa
70kDa
PKM2 58kDa
70kDa
50kDa
40kDa
β-actin 42kDa
50kDa
40kDa

Figure.1I

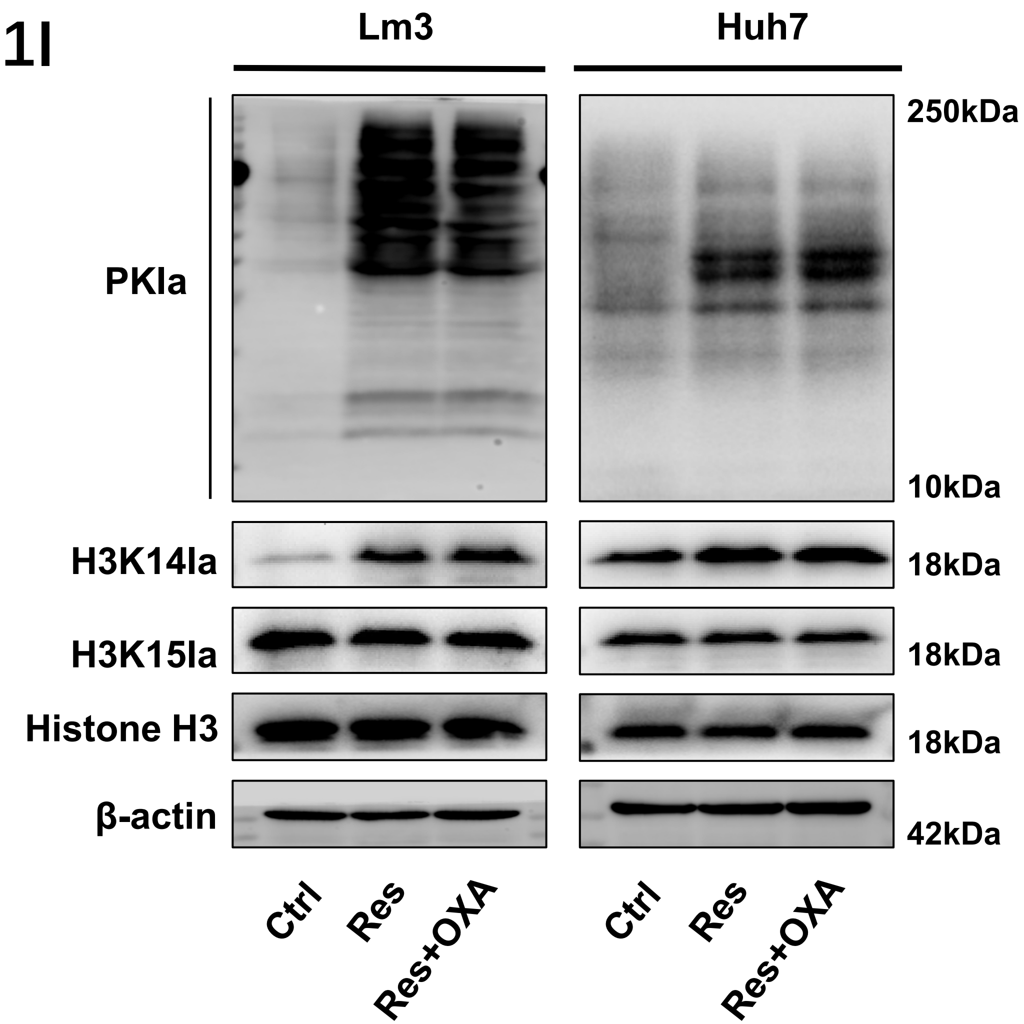

Lm3
Huh7
PKla
H3K14la
H3K15la
Histone H3
β-actin
250kDa
10kDa
18kDa
18kDa
18kDa
42kDa
Ctrl
Res
Res+OXA
Ctrl
Res
Res+OXA

Lm3

Pkla 10-250kDa

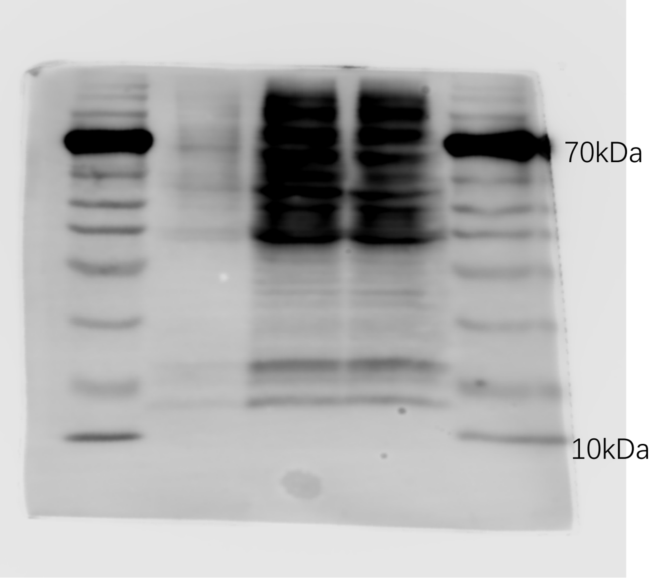

H3K14la 18kDa

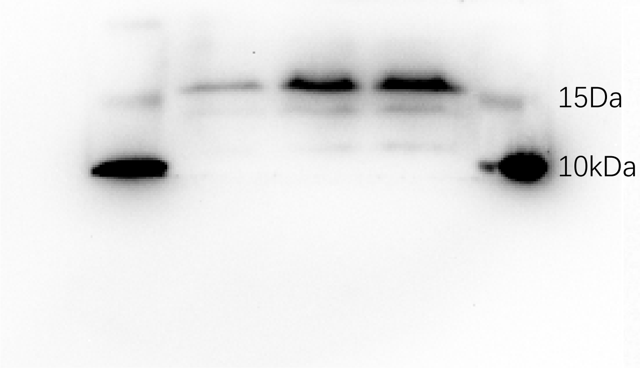

H3K15la 18kDa

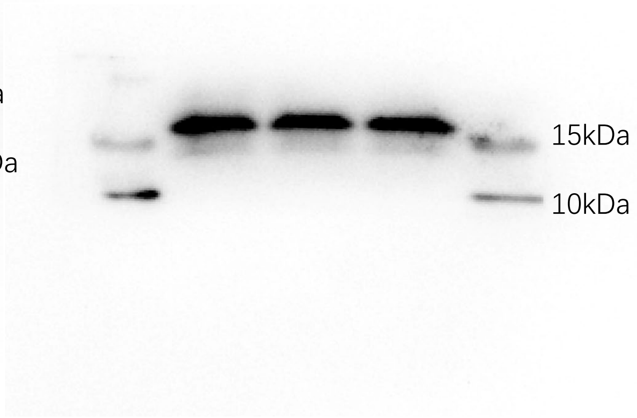

Histone H3 18kDa

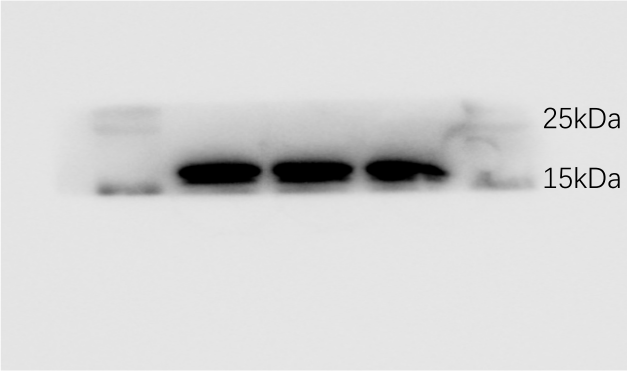

β-actin 42kDa

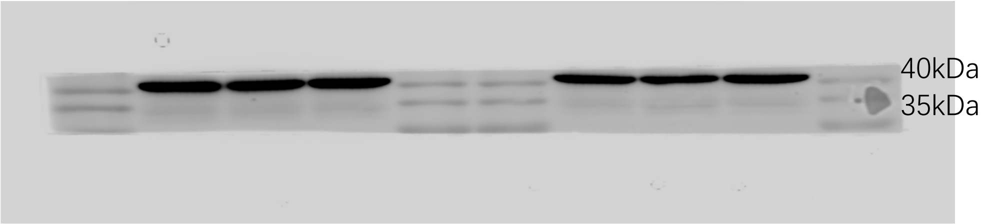

Huh7

Pkla 10-250kDa

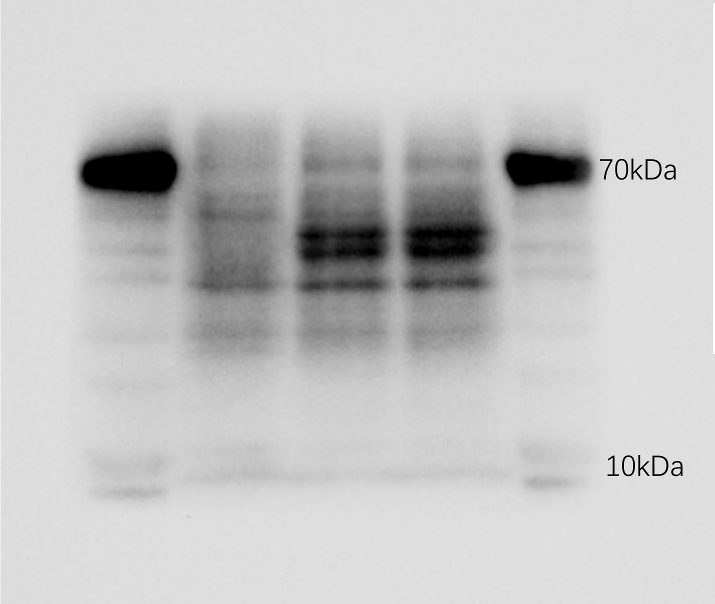

H3K14la 18kDa

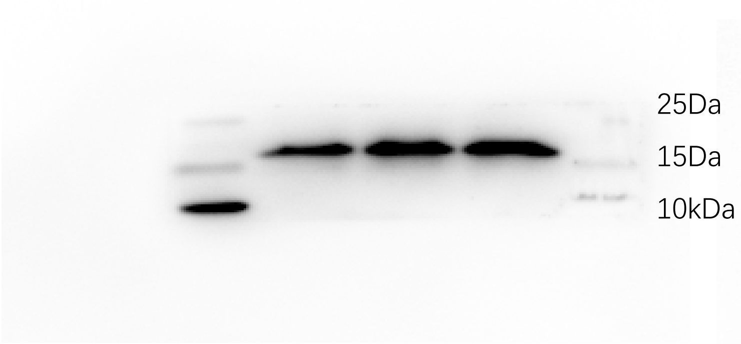

H3K15la 18kDa

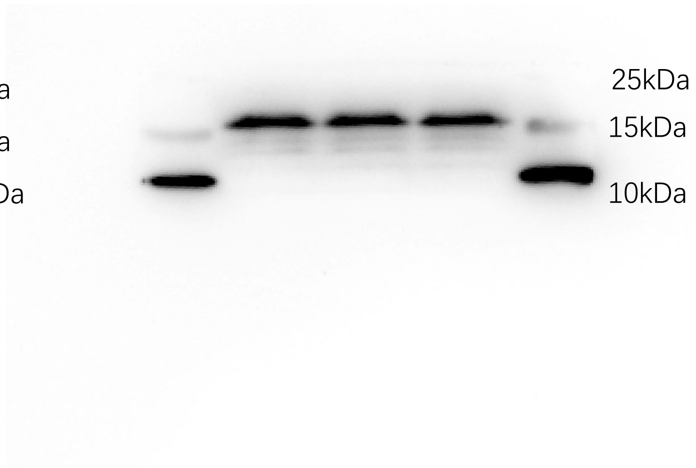

Histone H3 18kDa

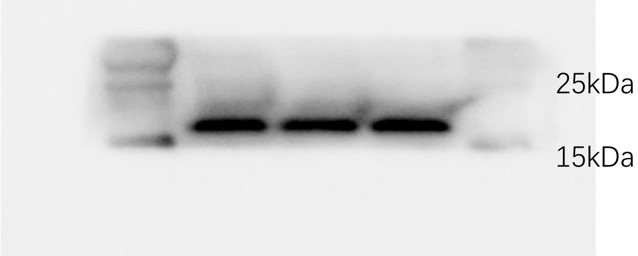

β-actin 42kDa

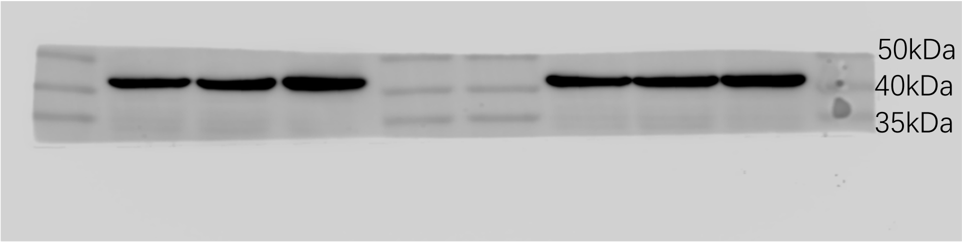

Figure.2B

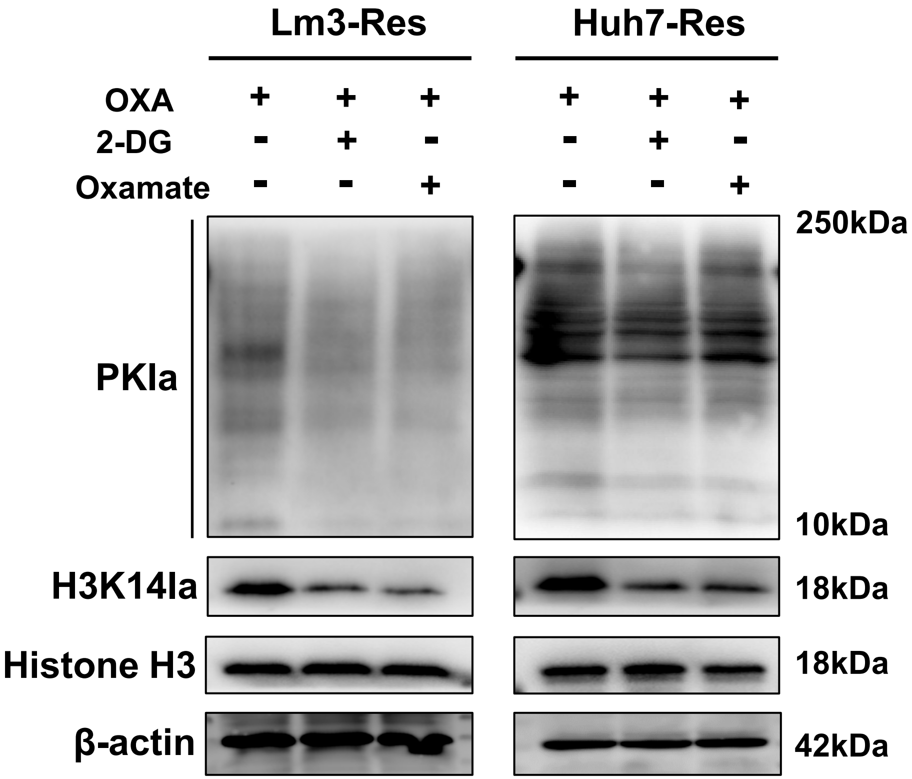
Lm3-Res
Huh7-Res
OXA + + + + + +
2-DG - + - - + -
Oxamate - - + - - +
PKla
H3K14la
Histone H3
β-actin
250kDa
10kDa
18kDa
18kDa
42kDa

Lm3-Res

Pkla 10-250kDa

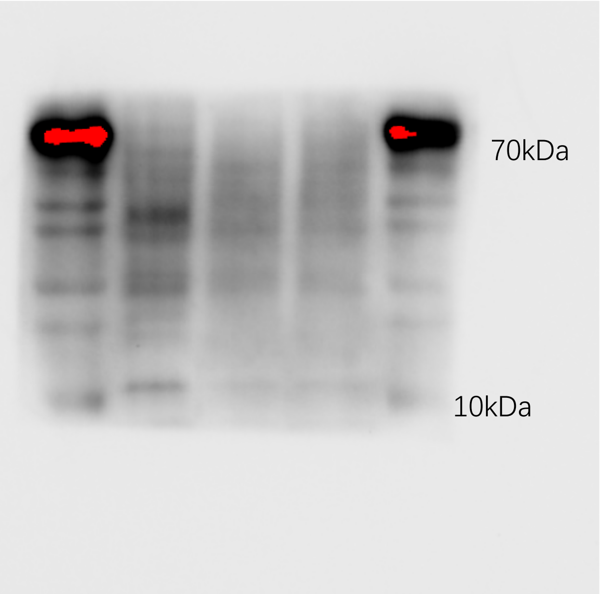

H3K14la 18kDa

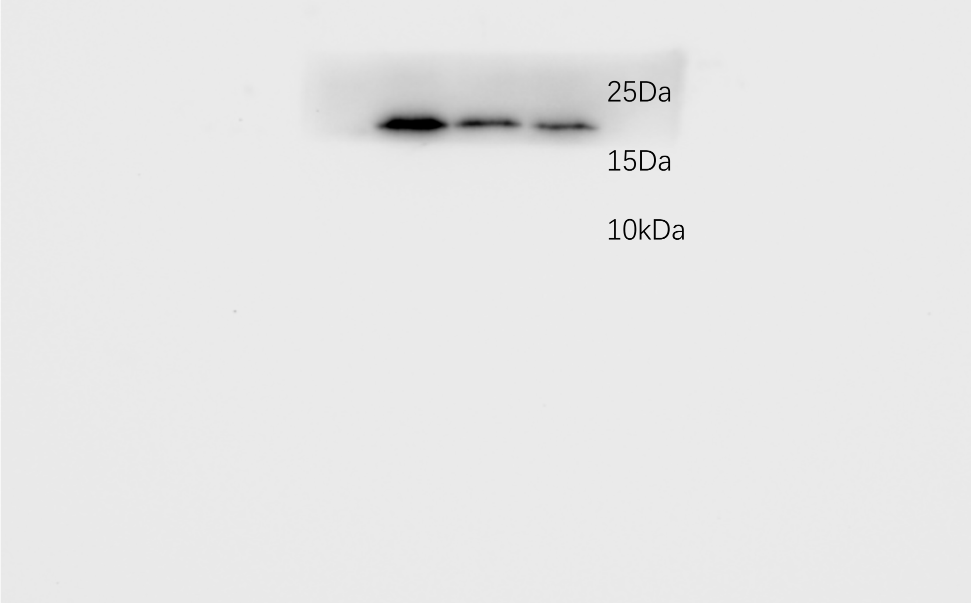

Histone H3 18kDa

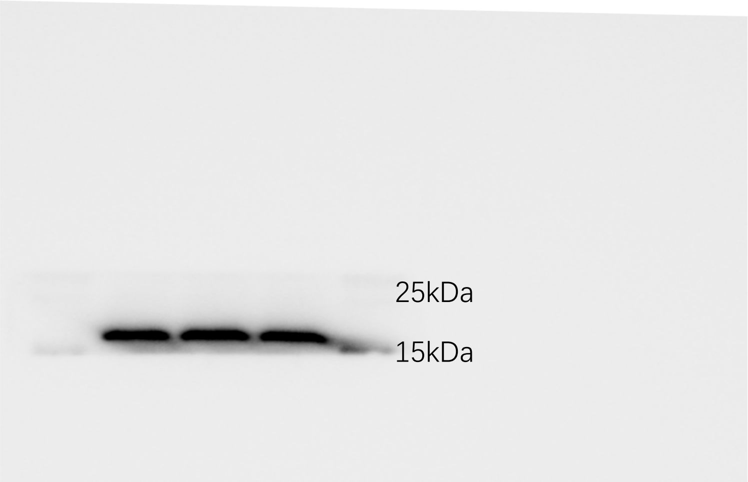

β-actin 42kDa

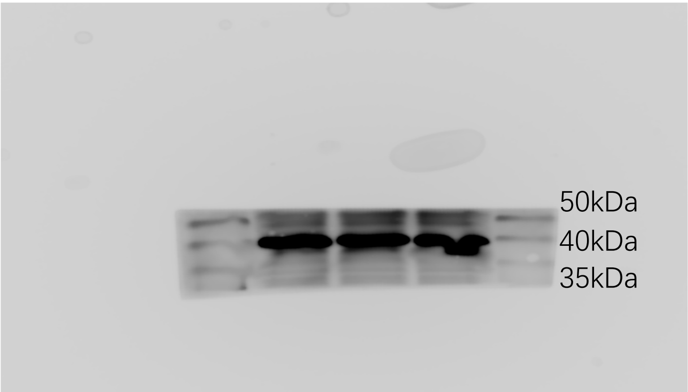

Huh7-Res

Pkla 10-250kDa

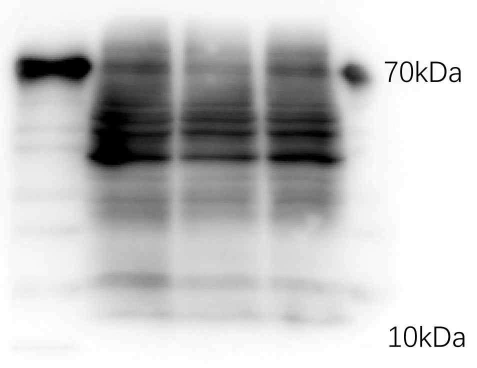

H3K14la 18kDa

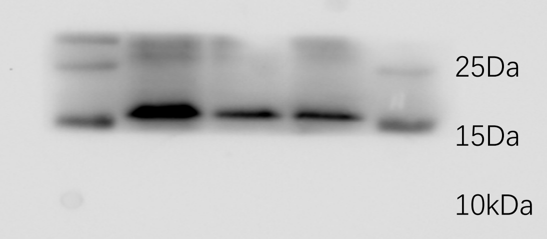

Histone H3 18kDa

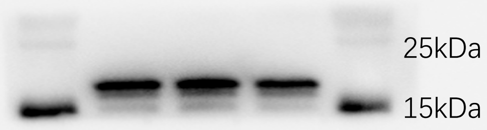

β-actin 42kDa

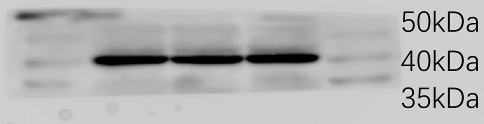

Figure.2F

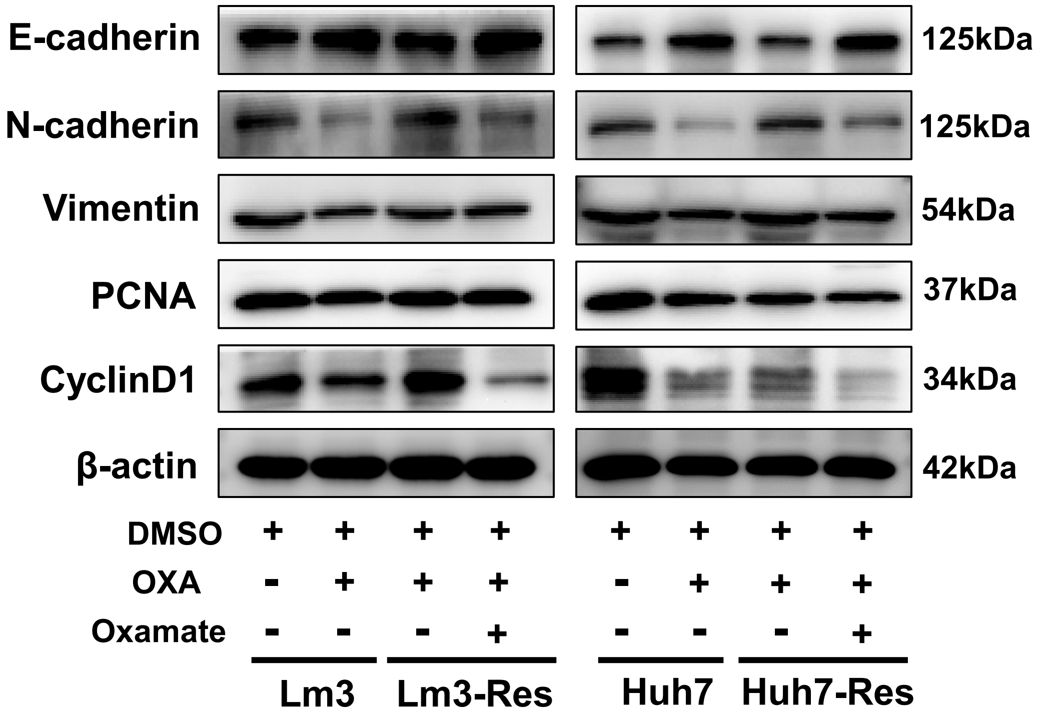
E-cadherin
N-cadherin
Vimentin
PCNA
CyclinD1
β-actin
125kDa
125kDa
54kDa
37kDa
34kDa
42kDa
DMSO + + + + + + + +
OXA - + + + - + + +
Oxamate - - - + - - - +
Lm3
Lm3-Res
Huh7
Huh7-Res

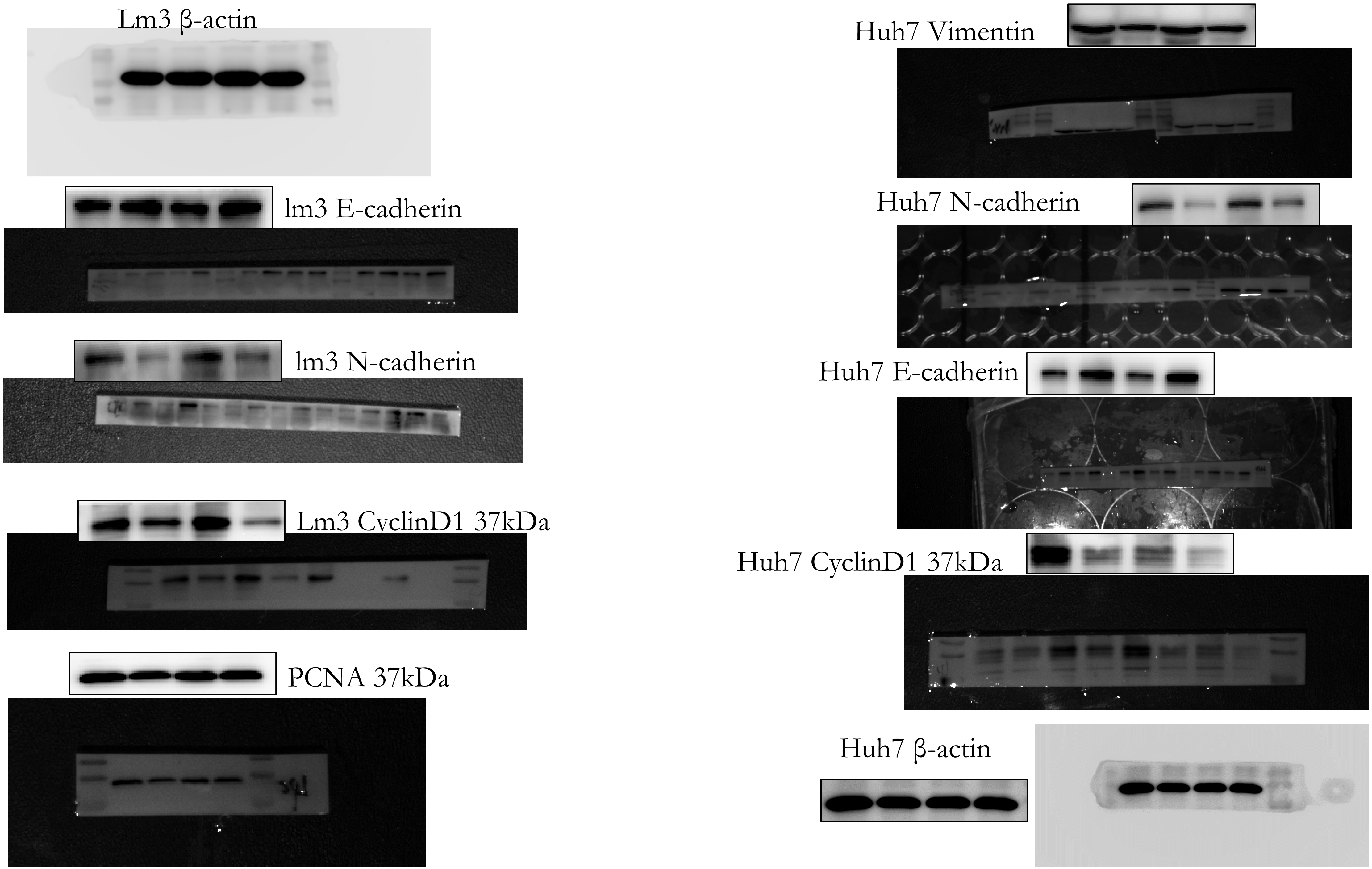
Lm3 β-actin
lm3 E-cadherin
lm3 N-cadherin
Lm3 CyclinD1 37kDa
PCNA 37kDa
Huh7 Vimentin
Huh7 N-cadherin
Huh7 E-cadherin
Huh7 CyclinD1 37kDa
Huh7 β-actin

Figure.3G

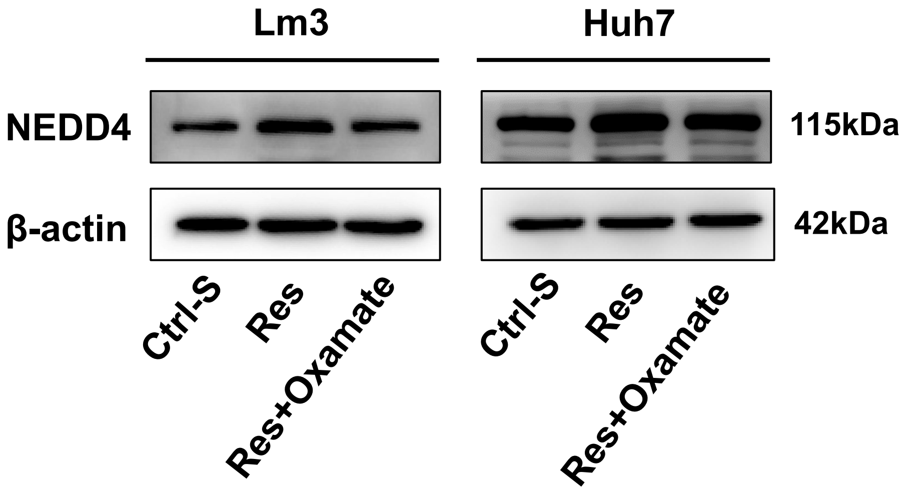
Lm3
Huh7
NEDD4
β-actin
115kDa
42kDa
Ctrl-S
Res
Res+Oxamate
Ctrl-S
Res
Res+Oxamate

Lm3 NEDD4 115kDa

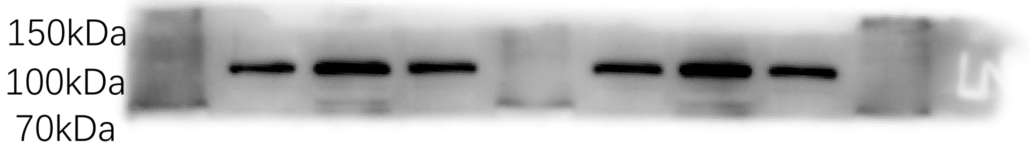

Huh7 NEDD4 115kDa

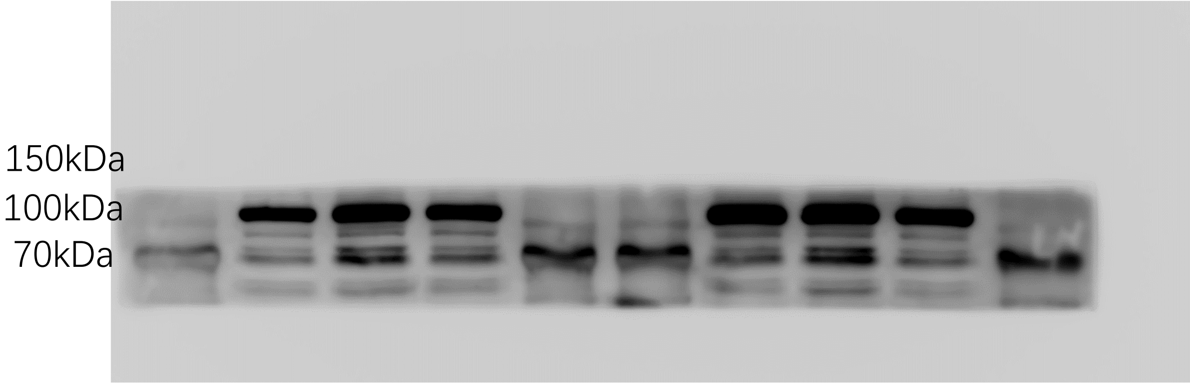

Lm3 β-actin 42kDa

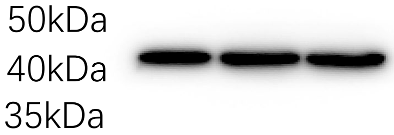

Huh7 β-actin 42kDa

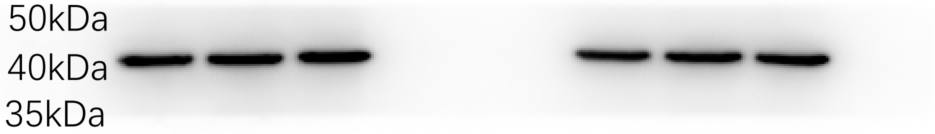

Figure.3H

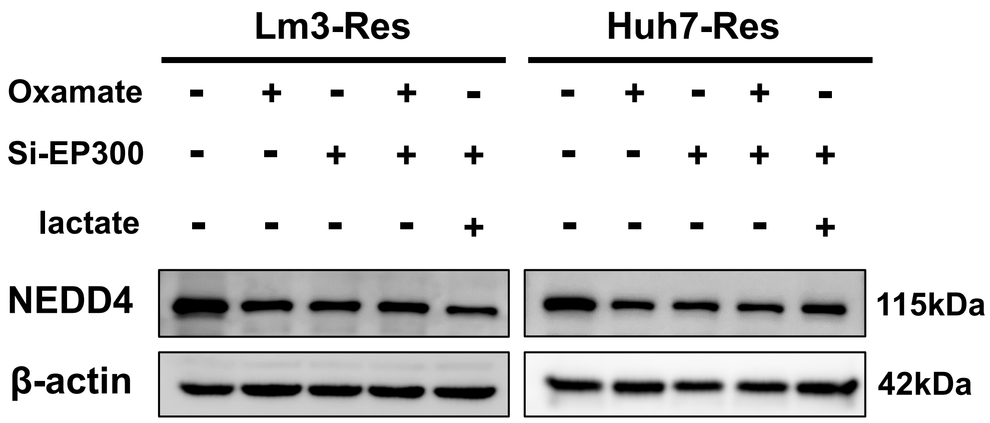
Lm3-Res
Huh7-Res
Oxamate - + - + - - + - + -
Si-EP300 - - + + + - - + + +
lactate - - - - + - - - - +
NEDD4
β-actin
115kDa
42kDa

Huh7 NEDD4 115kDa

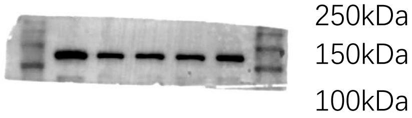

Lm3 NEDD4 115kDa

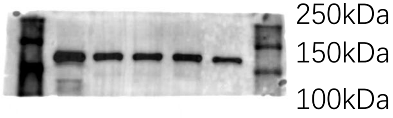

Lm3 β-actin 42kDa

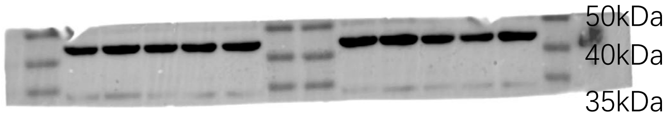

Huh7 β-actin 42kDa

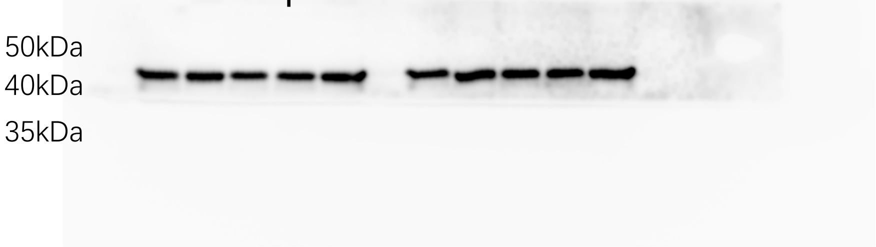

Figure.4E

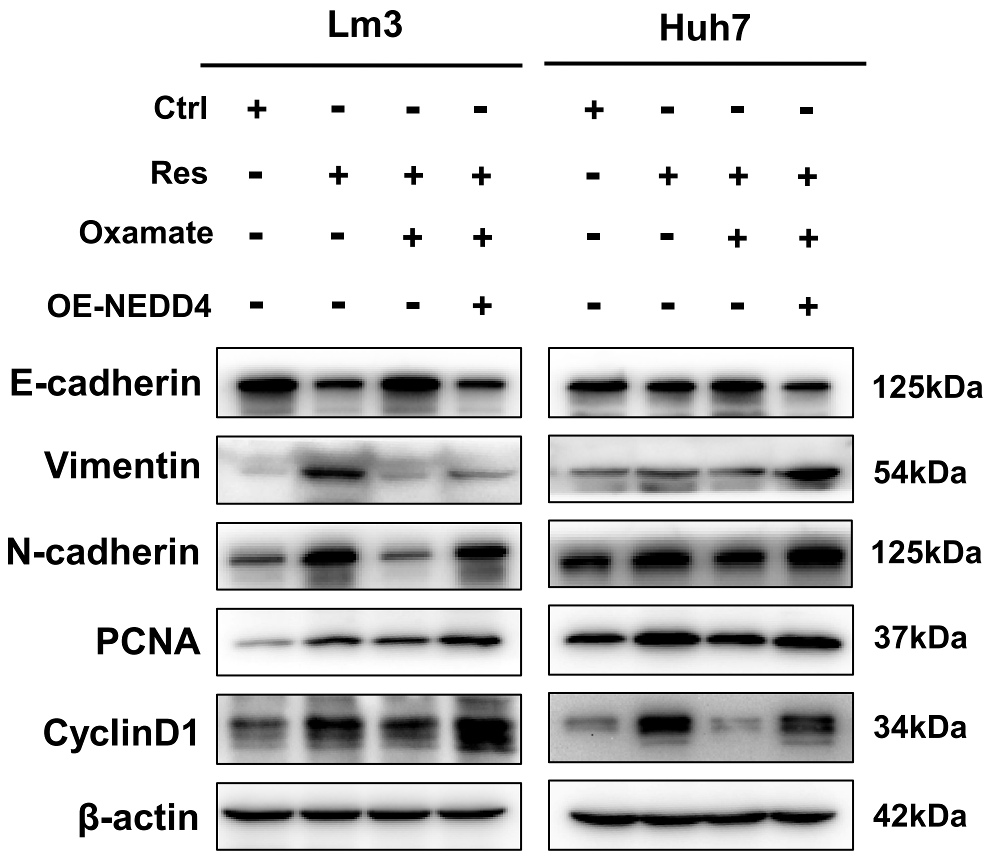
Lm3
Huh7
Ctrl + - - - + - - -
Res - + + + - + + +
Oxamate - - + + - - + +
OE-NEDD4 - - - + - - - +
E-cadherin
Vimentin
N-cadherin
PCNA
CyclinD1
β-actin
125kDa
54kDa
125kDa
37kDa
34kDa
42kDa

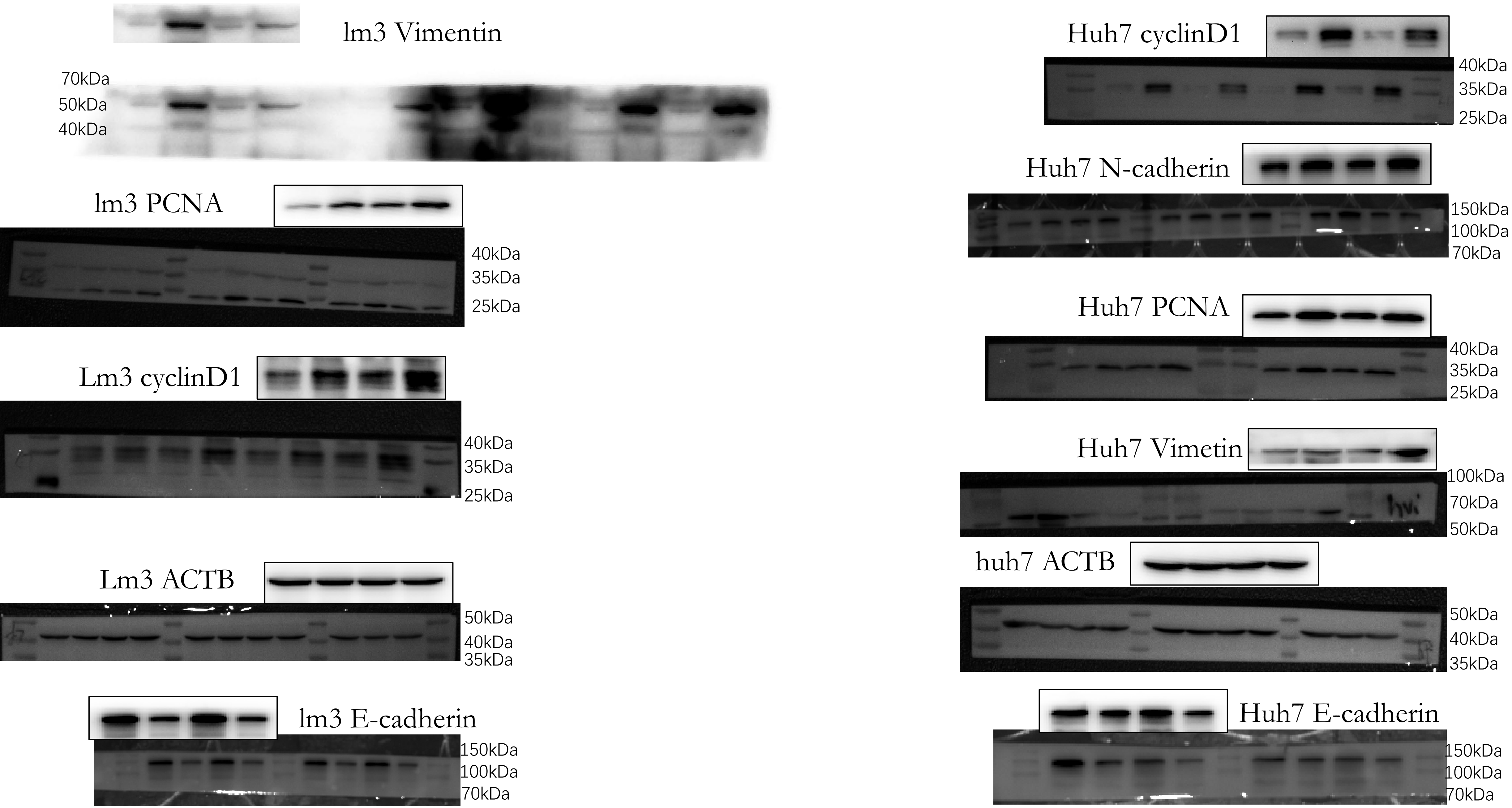
lm3 Vimentin
70kDa
50kDa
40kDa
lm3 PCNA
40kDa
35kDa
25kDa
Lm3 cyclinD1
40kDa
35kDa
25kDa
Lm3 ACTB
50kDa
40kDa
35kDa
lm3 E-cadherin
150kDa
100kDa
70kDa
Huh7 cyclinD1
40kDa
35kDa
25kDa
Huh7 N-cadherin
150kDa
100kDa
70kDa
Huh7 PCNA
40kDa
35kDa
25kDa
Huh7 Vimetin
100kDa
70kDa
50kDa
huh7 ACTB
50kDa
40kDa
35kDa
Huh7 E-cadherin
150kDa
100kDa
70kDa

Figure.5B

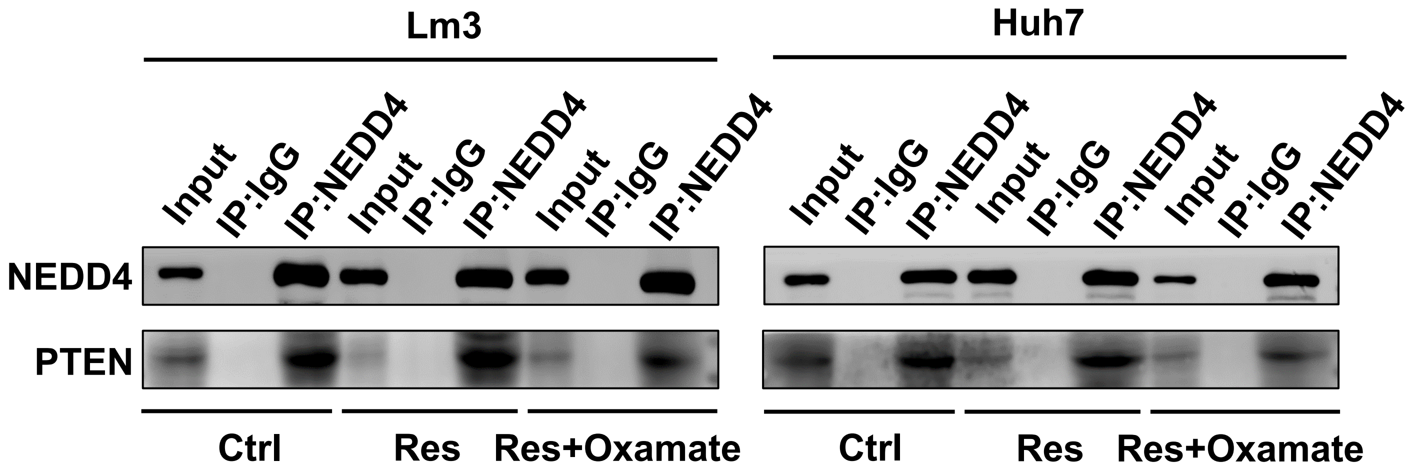

Lm3
Huh7
Input
IP:IgG
IP:NEDD4
Input
IP:IgG
IP:NEDD4
Input
IP:IgG
IP:NEDD4
Input
IP:IgG
IP:NEDD4
Input
IP:IgG
IP:NEDD4
Input
IP:IgG
IP:NEDD4
NEDD4
PTEN
Ctrl
Res
Res+Oxamate
Ctrl
Res
Res+Oxamate

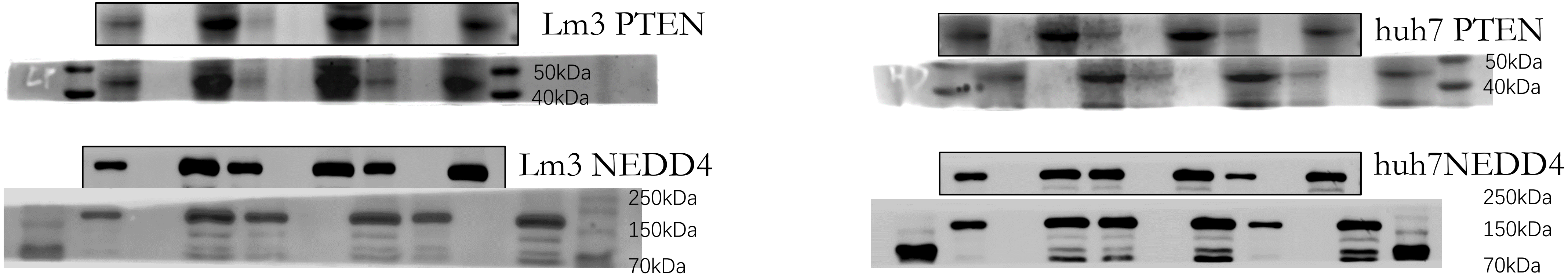
Lm3 PTEN
50kDa
40kDa
Lm3 NEDD4
250kDa
150kDa
70kDa
huh7 PTEN
50kDa
40kDa
huh7NEDD4
250kDa
150kDa
70kDa

Figure.5C

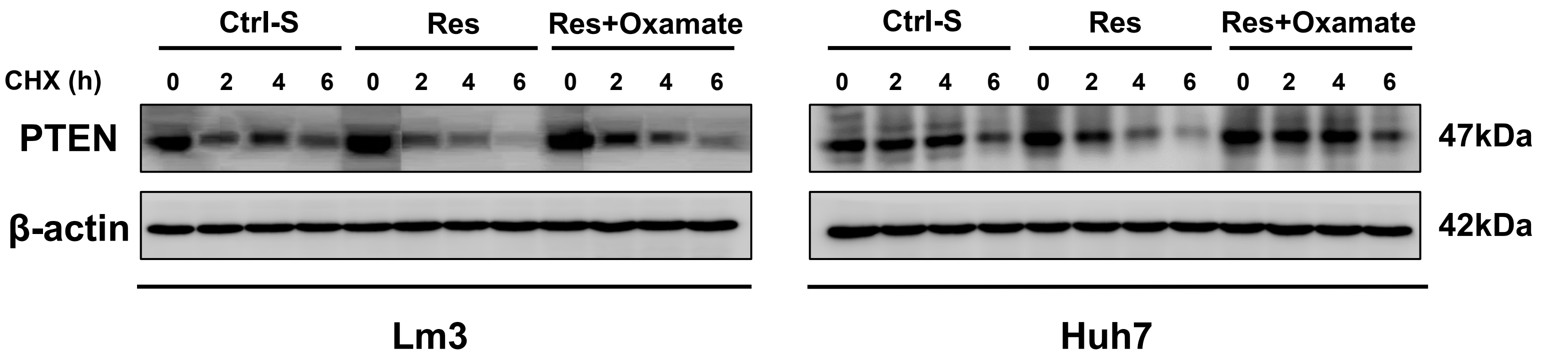
Ctrl-S
Res
Res+Oxamate
CHX (h)
0 2 4 6 0 2 4 6 0 2 4 6
PTEN
β-actin
47kDa
42kDa
Lm3
Huh7

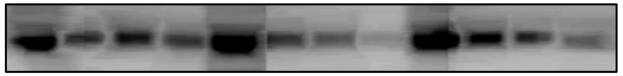

Lm3 PTEN

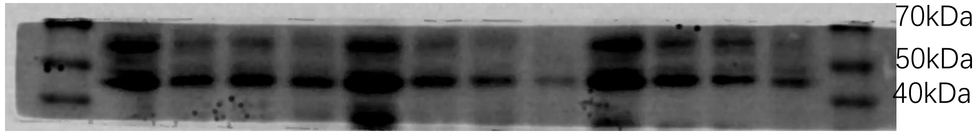

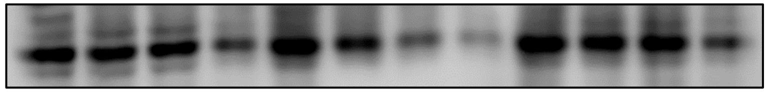

huh7 PTEN

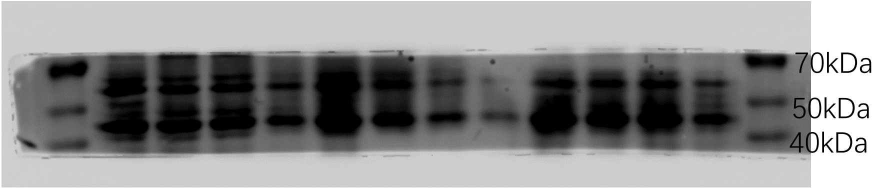

Lm3 ACTB

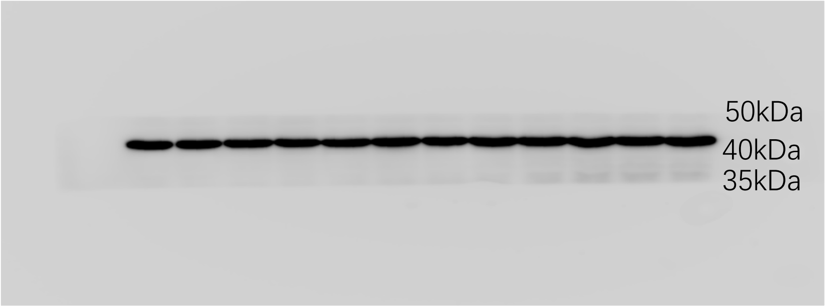

Huh7 ACTB

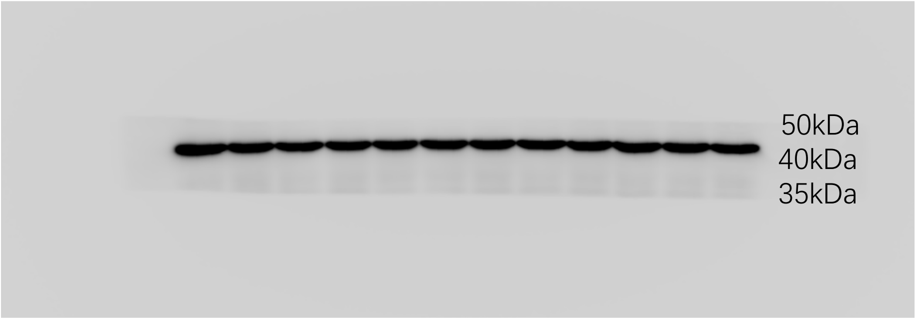

Figure.5D

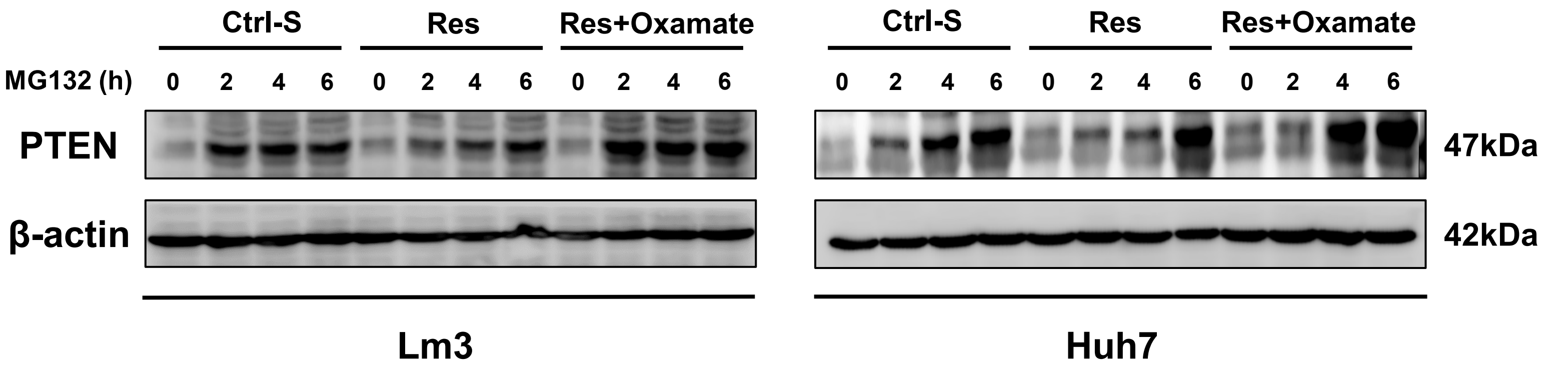
Ctrl-S
Res
Res+Oxamate
MG132 (h)
0 2 4 6 0 2 4 6 0 2 4 6
PTEN
β-actin
47kDa
42kDa
Lm3
Huh7

Lm3 PTEN

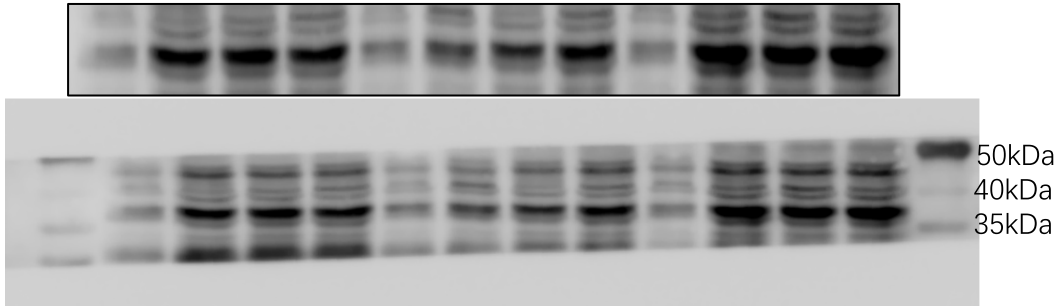

huh7 PTEN

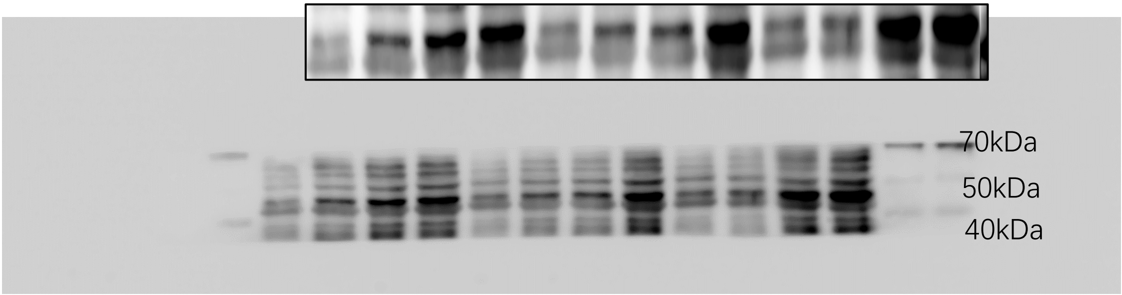

Lm3 actb

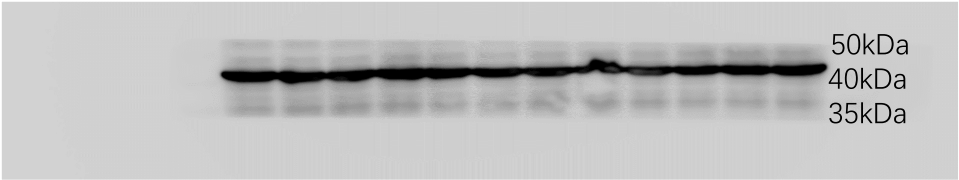

Huh7 actb

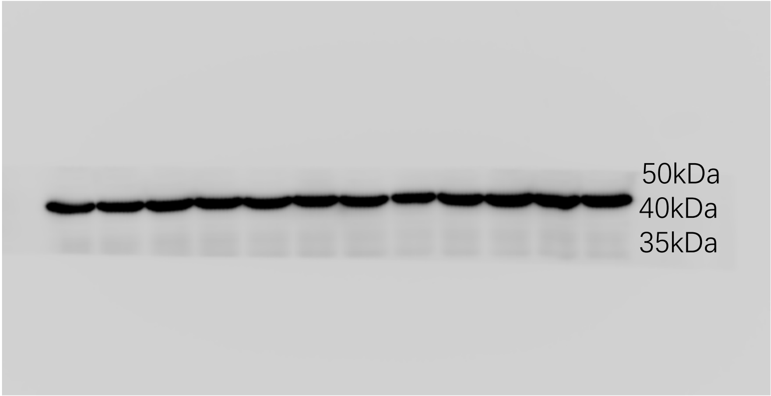

Figure.5H

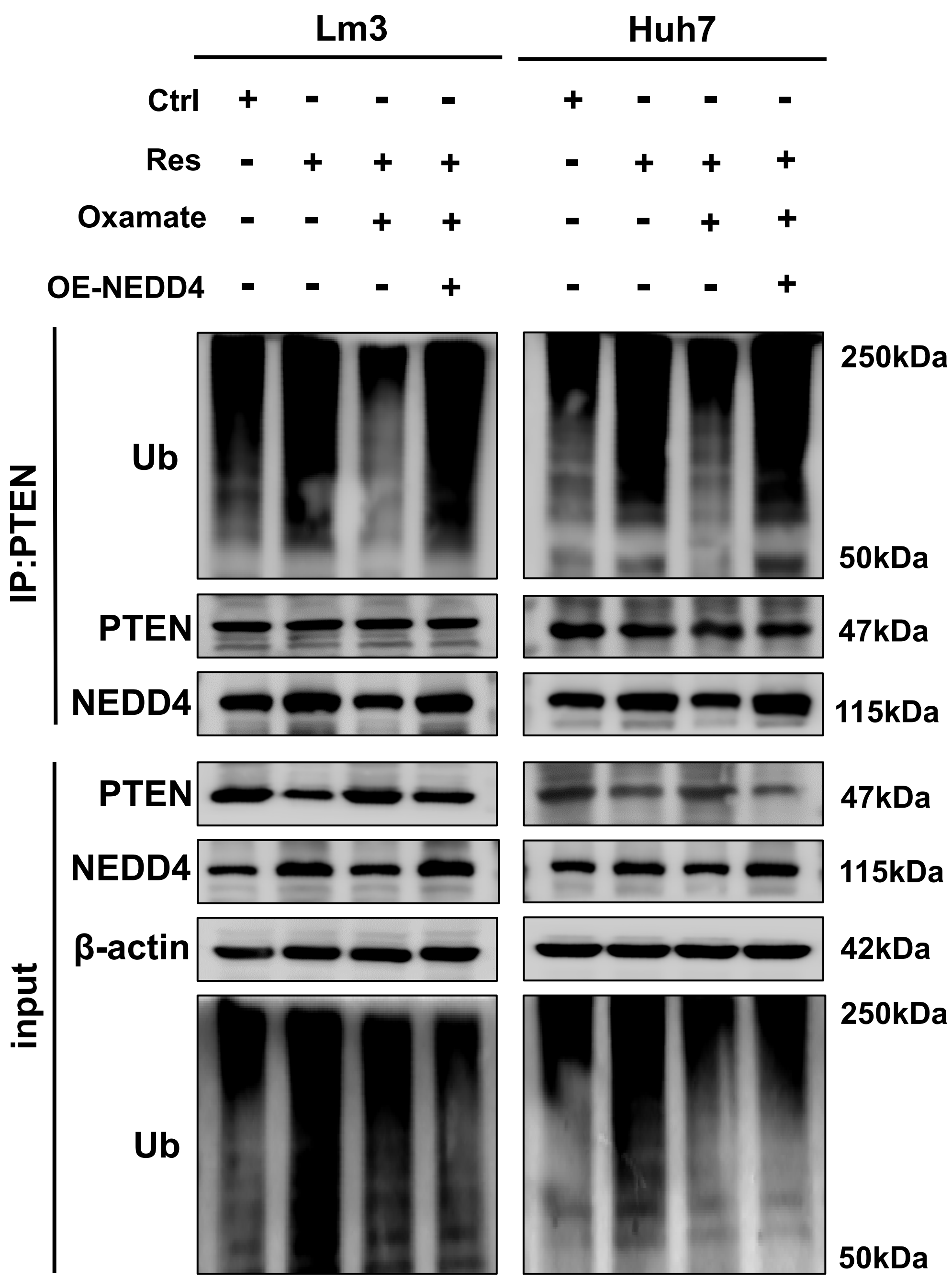
Lm3
Huh7
Ctrl
Res
Oxamate
OE-NEDD4
IP:PTEN
Ub
PTEN
NEDD4
input
PTEN
NEDD4
β-actin
Ub
250kDa
50kDa
47kDa
115kDa
47kDa
115kDa
42kDa
250kDa
50kDa

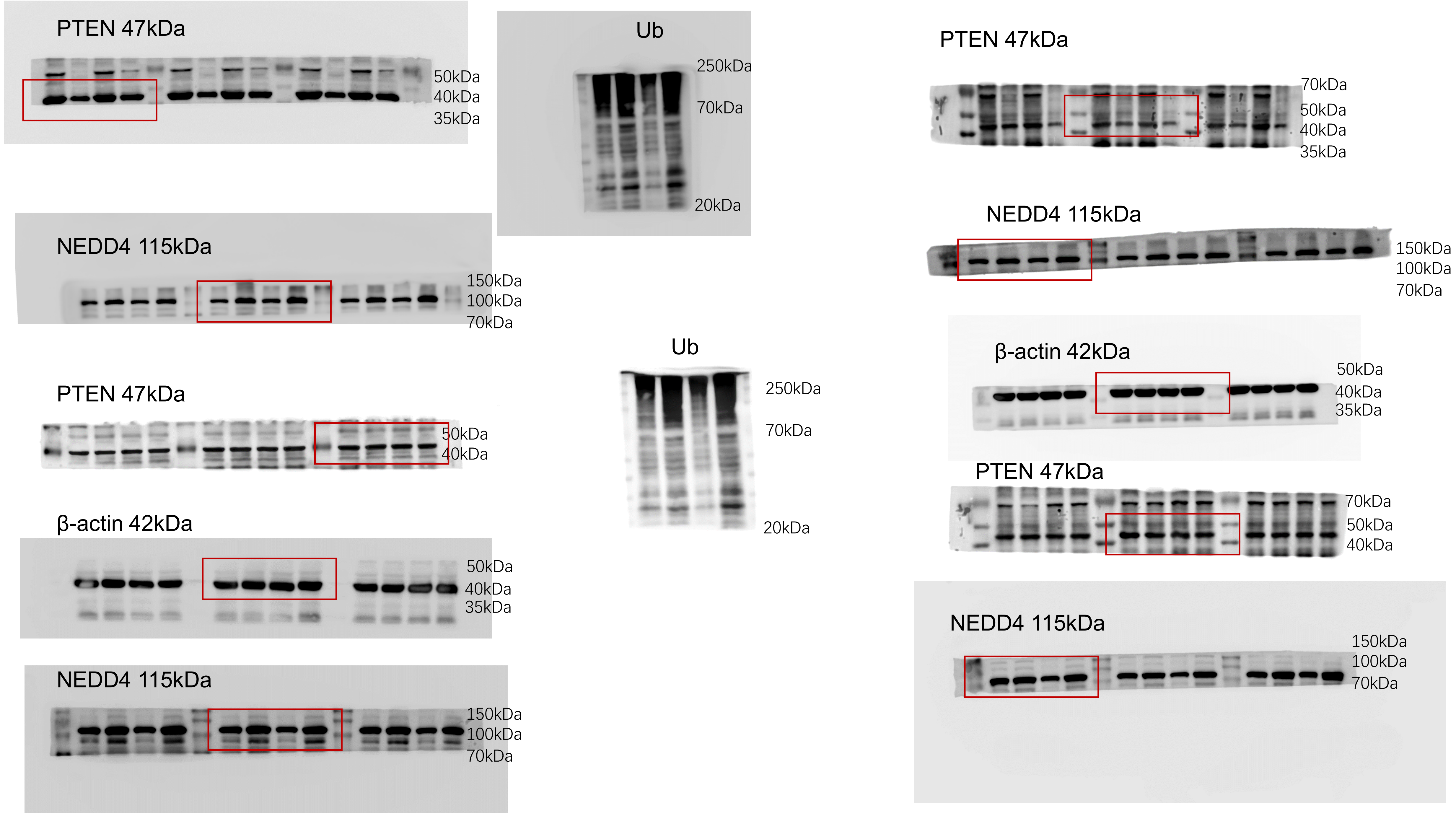

PTEN 47kDa
50kDa
40kDa
35kDa
Ub
250kDa
70kDa
20kDa
PTEN 47kDa
70kDa
50kDa
40kDa
35kDa
NEDD4 115kDa
150kDa
100kDa
70kDa
NEDD4 115kDa
150kDa
100kDa
70kDa
Ub
250kDa
70kDa
20kDa
β-actin 42kDa
50kDa
40kDa
35kDa
PTEN 47kDa
50kDa
40kDa
PTEN 47kDa
70kDa
50kDa
40kDa
β-actin 42kDa
50kDa
40kDa
35kDa
NEDD4 115kDa
150kDa
100kDa
70kDa
NEDD4 115kDa
150kDa
100kDa
70kDa

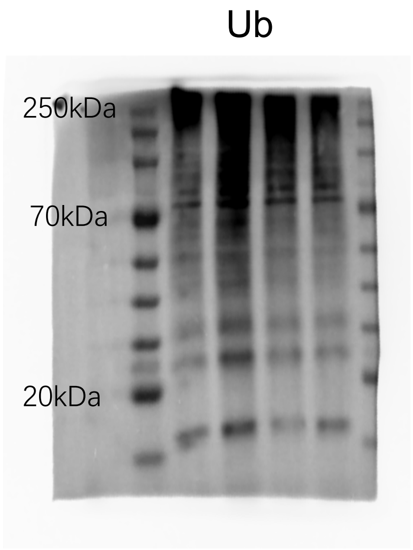
Ub
250kDa
70kDa
20kDa

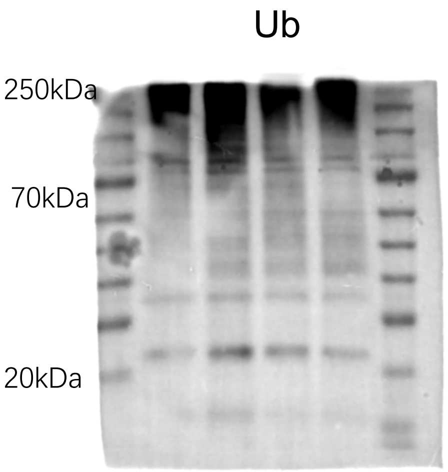
Ub
250kDa
70kDa
20kDa

Figure.5H

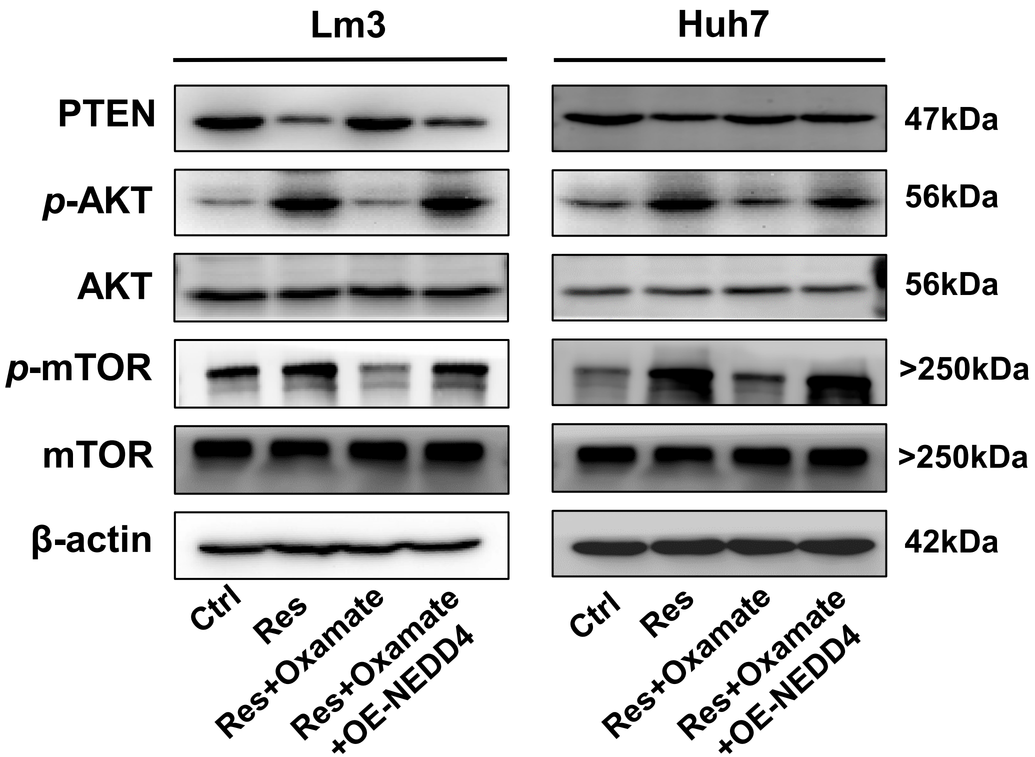
Lm3
Huh7
PTEN
p-AKT
AKT
p-mTOR
mTOR
β-actin
47kDa
56kDa
56kDa
>250kDa
>250kDa
42kDa
Ctrl
Res
Res+Oxamate
Res+Oxamate +OE-NEDD4
Ctrl
Res
Res+Oxamate
Res+Oxamate +OE-NEDD4

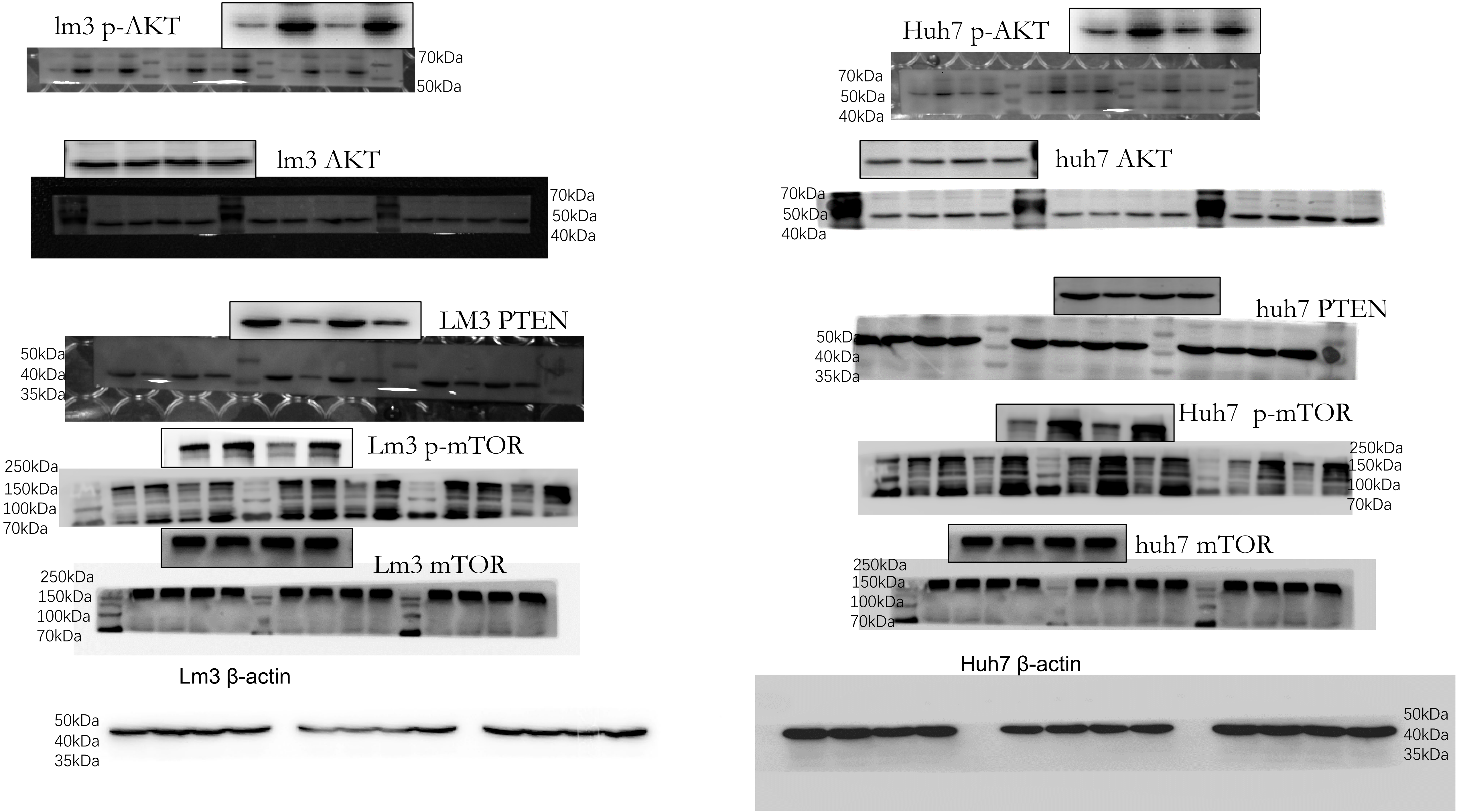

lm3 p-AKT
70kDa
50kDa
lm3 AKT
70kDa
50kDa
40kDa
LM3 PTEN
50kDa
40kDa
35kDa
Lm3 p-mTOR
250kDa
150kDa
100kDa
70kDa
Lm3 mTOR
250kDa
150kDa
100kDa
70kDa
Lm3 β-actin
50kDa
40kDa
35kDa
Huh7 p-AKT
70kDa
50kDa
40kDa
huh7 AKT
70kDa
50kDa
40kDa
huh7 PTEN
50kDa
40kDa
35kDa
Huh7 p-mTOR
250kDa
150kDa
100kDa
70kDa
huh7 mTOR
250kDa
150kDa
100kDa
70kDa
Huh7 β-actin
50kDa
40kDa
35kDa

Figure.6C

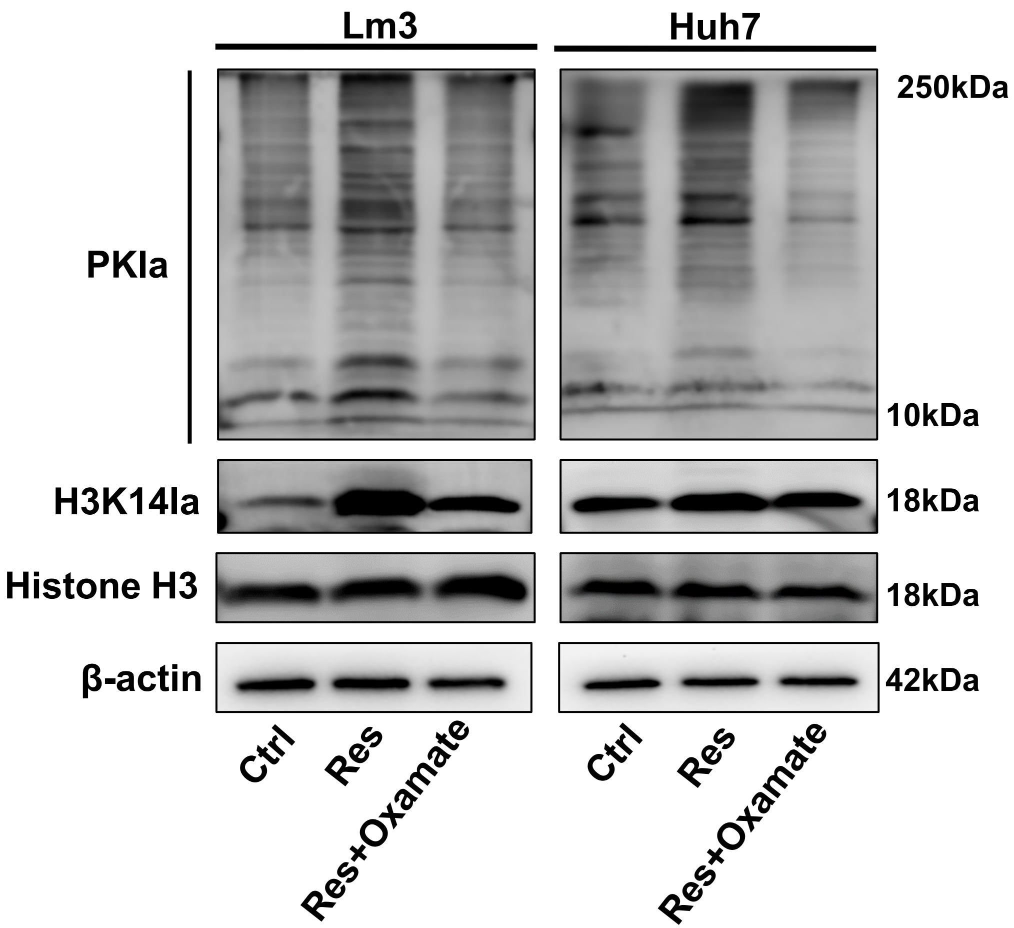
Lm3
Huh7
250kDa
PKla
10kDa
H3K14la
18kDa
Histone H3
18kDa
β-actin
42kDa
Ctrl
Res
Res+Oxamate
Ctrl
Res
Res+Oxamate

Lm3

Pkla 10-250kDa

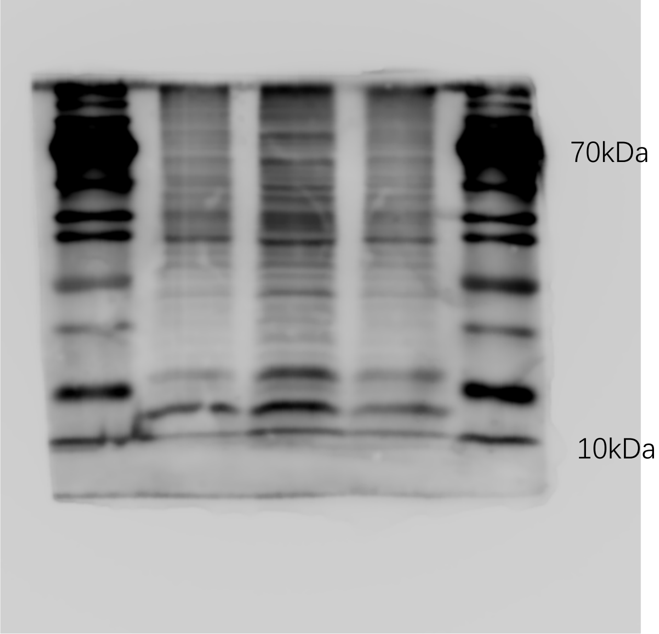

H3K14la 18kDa

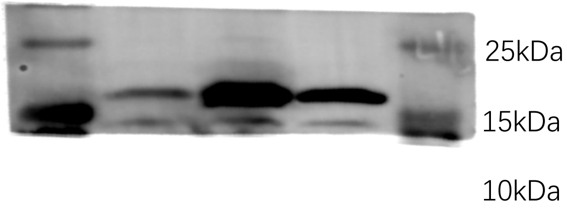

Histone H3 18kDa

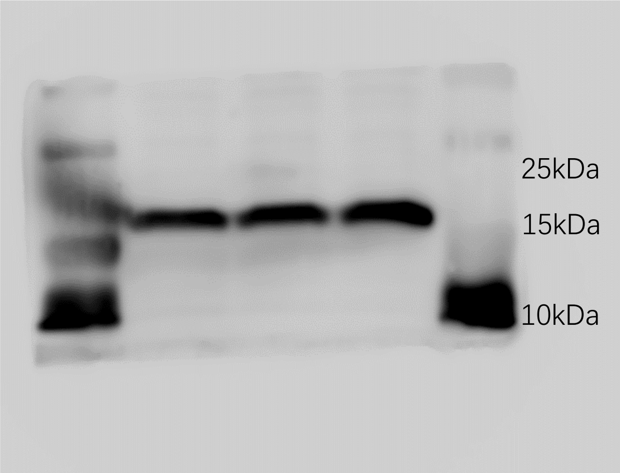

β-actin 42kDa

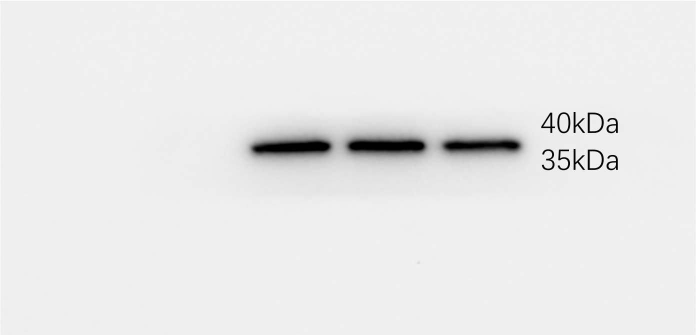

Lm3

Pkla 10-250kDa

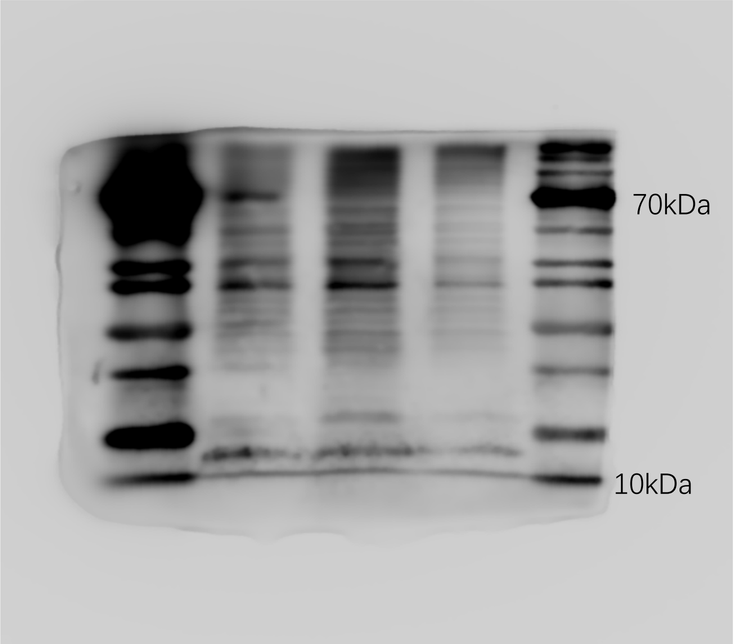

H3K14la 18kDa

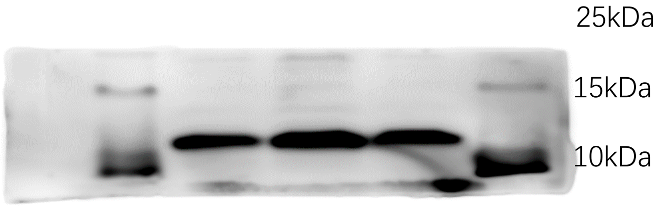

Histone H3 18kDa

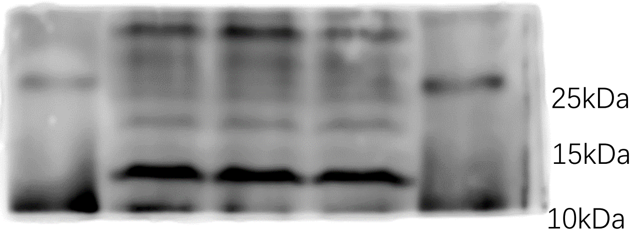

β-actin 42kDa

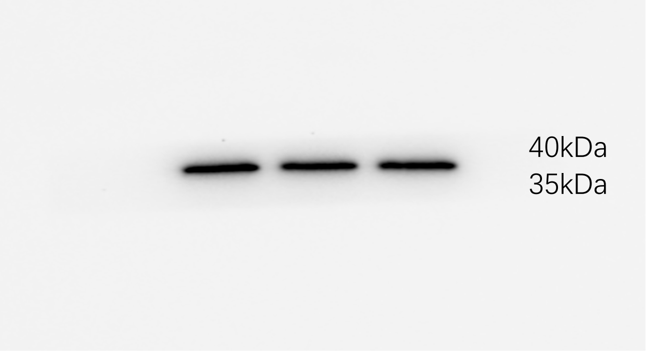

Figure.6D

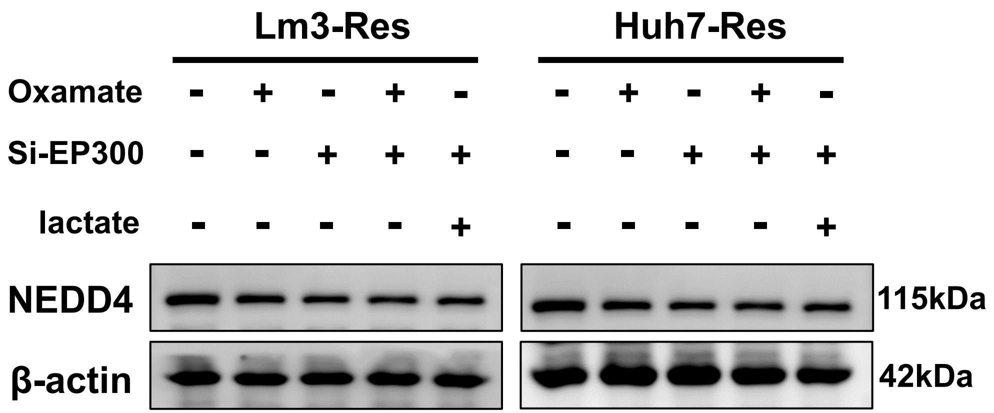
Lm3-Res
Huh7-Res
Oxamate - + - + - - + - + -
Si-EP300 - - + + + - - + + +
lactate - - - - + - - - - +
NEDD4
β-actin
115kDa
42kDa

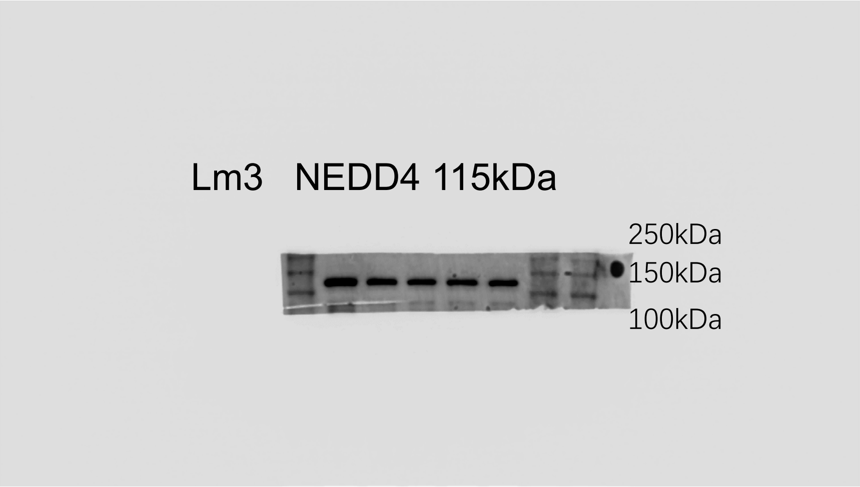

Huh7 NEDD4 115kDa

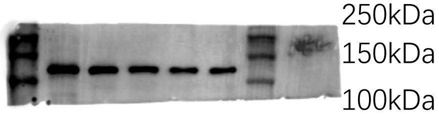

Lm3 β-actin 42kDa

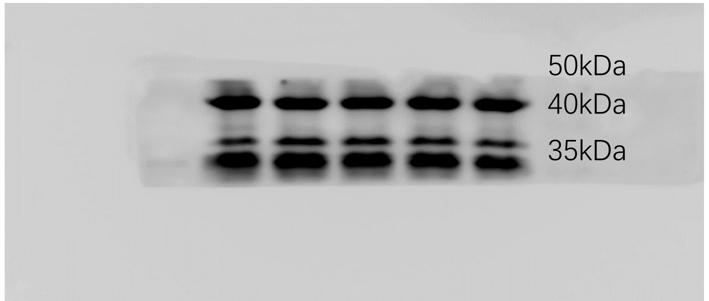

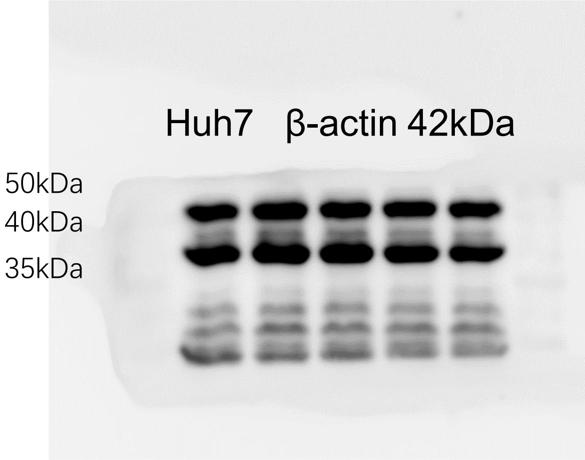

Figure.6E

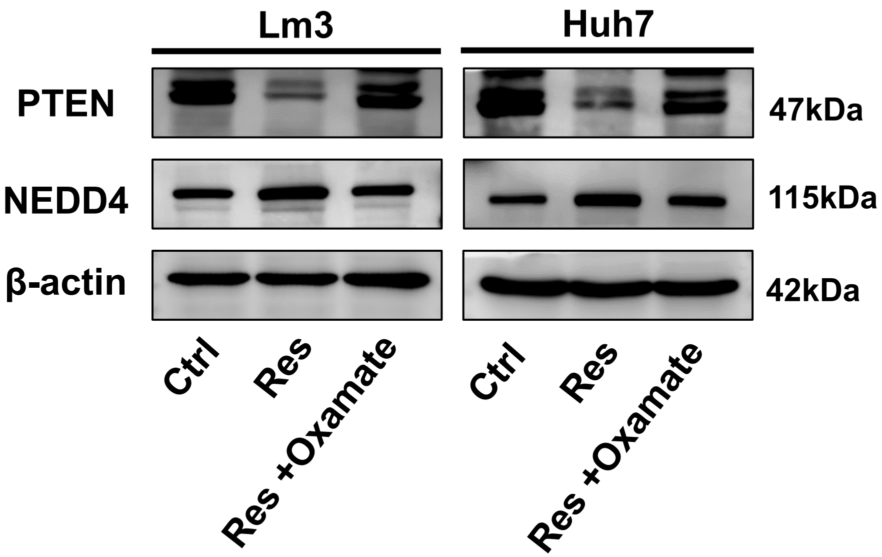

Lm3
Huh7
PTEN
47kDa
NEDD4
115kDa
β-actin
42kDa
Ctrl
Res
Res +Oxamate
Ctrl
Res
Res +Oxamate

LM3 NEDD4

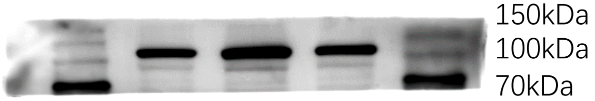

huh7 NEDD4

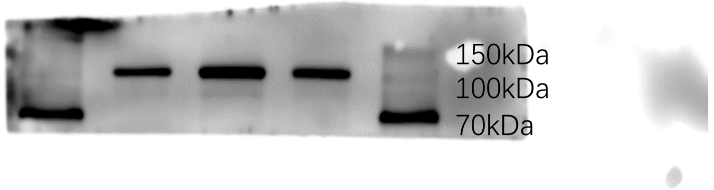

LM3 PTEN

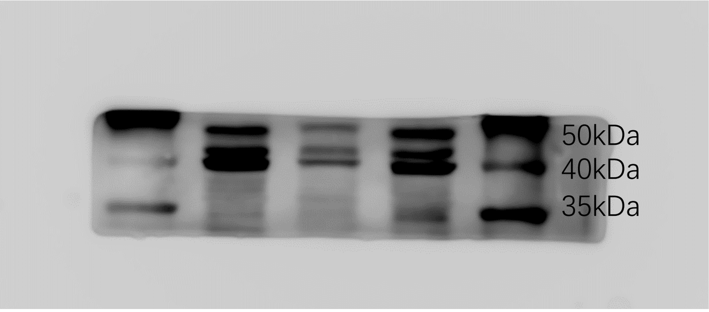

Huh7 PTEN

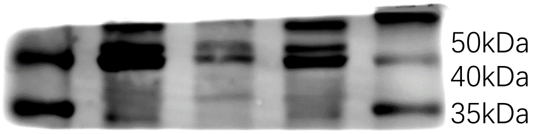

LM3 ACTB

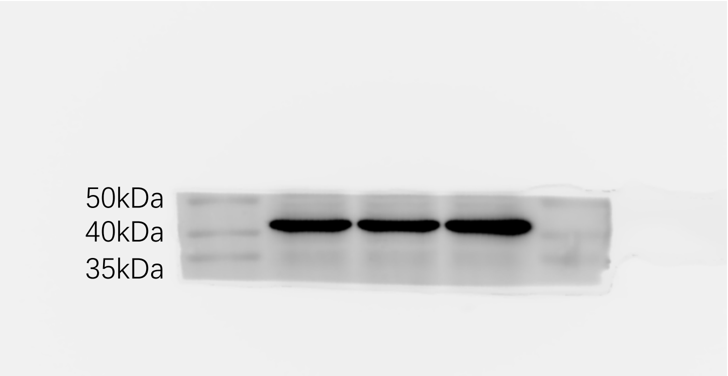

Huh7 ACTB

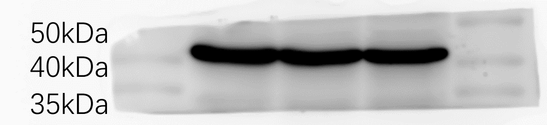

Figure.6H

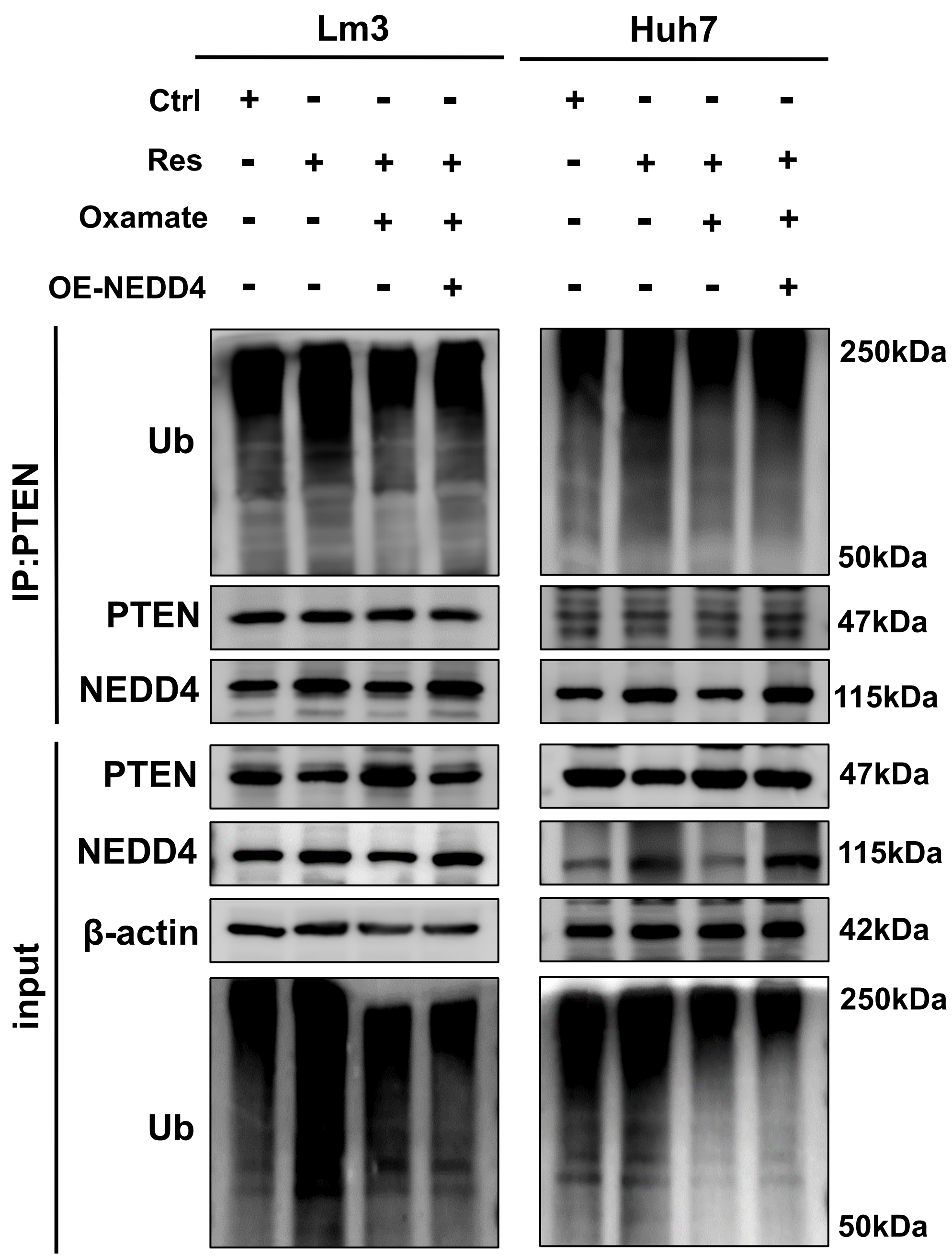
Lm3
Huh7
Ctrl + - - - + - - -
Res - + + + - + + +
Oxamate - - + + - - + +
OE-NEDD4 - - - + - - - +
IP:PTEN
Ub
PTEN
NEDD4
input
PTEN
NEDD4
β-actin
Ub
250kDa
50kDa
47kDa
115kDa
47kDa
115kDa
42kDa
250kDa
50kDa

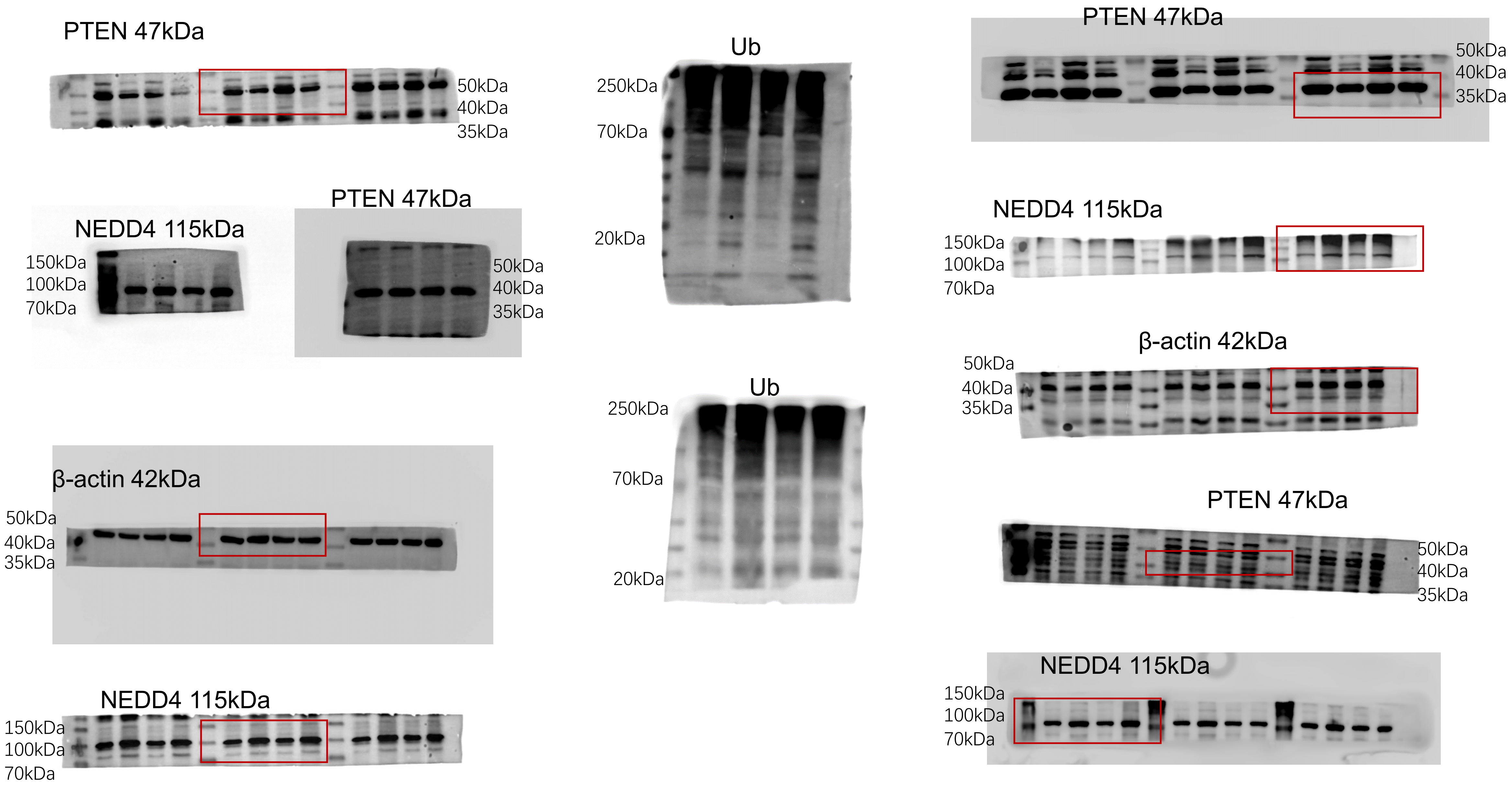
PTEN 47kDa
50kDa
40kDa
35kDa
NEDD4 115kDa
150kDa
100kDa
70kDa
PTEN 47kDa
50kDa
40kDa
35kDa
β-actin 42kDa
50kDa
40kDa
35kDa
NEDD4 115kDa
150kDa
100kDa
70kDa
Ub
250kDa
70kDa
20kDa
Ub
250kDa
70kDa
20kDa
PTEN 47kDa
50kDa
40kDa
35kDa
NEDD4 115kDa
150kDa
100kDa
70kDa
β-actin 42kDa
50kDa
40kDa
35kDa
PTEN 47kDa
50kDa
40kDa
35kDa
NEDD4 115kDa
150kDa
100kDa
70kDa

Figure.7E

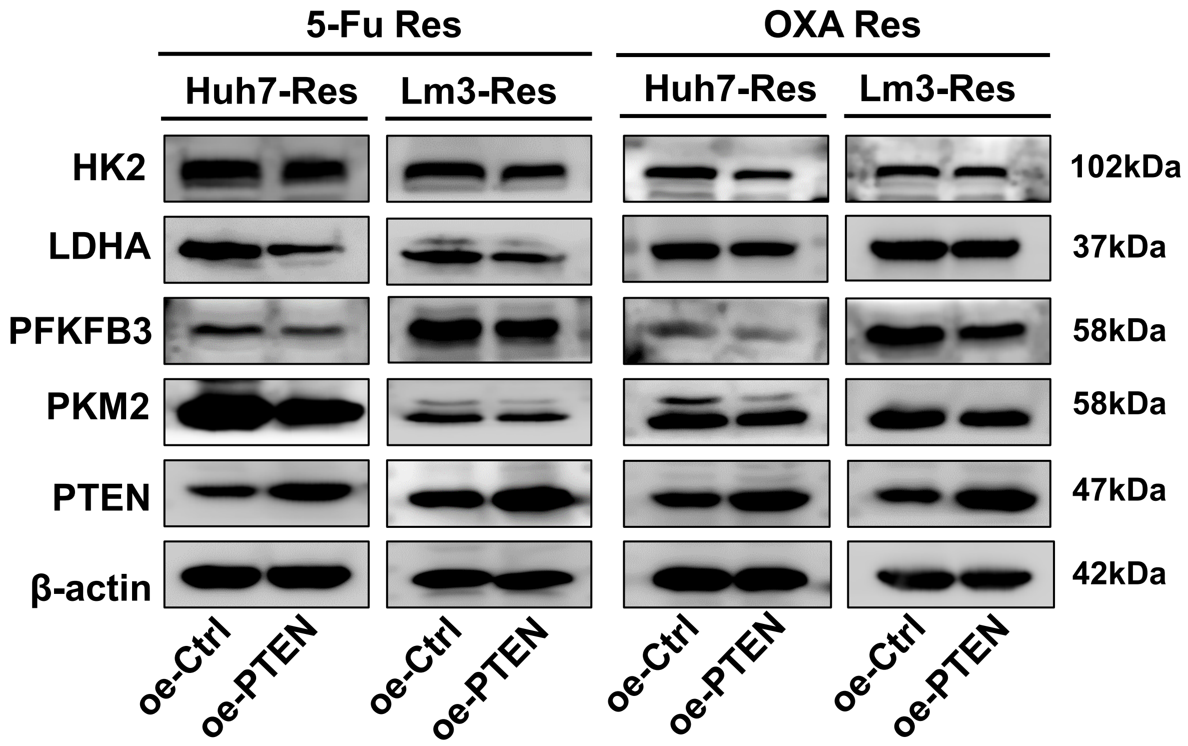
5-Fu Res
OXA Res
Huh7-Res
Lm3-Res
Huh7-Res
Lm3-Res
HK2
LDHA
PFKFB3
PKM2
PTEN
β-actin
102kDa
37kDa
58kDa
58kDa
47kDa
42kDa
oe-Ctrl
oe-PTEN
oe-Ctrl
oe-PTEN
oe-Ctrl
oe-PTEN
oe-Ctrl
oe-PTEN

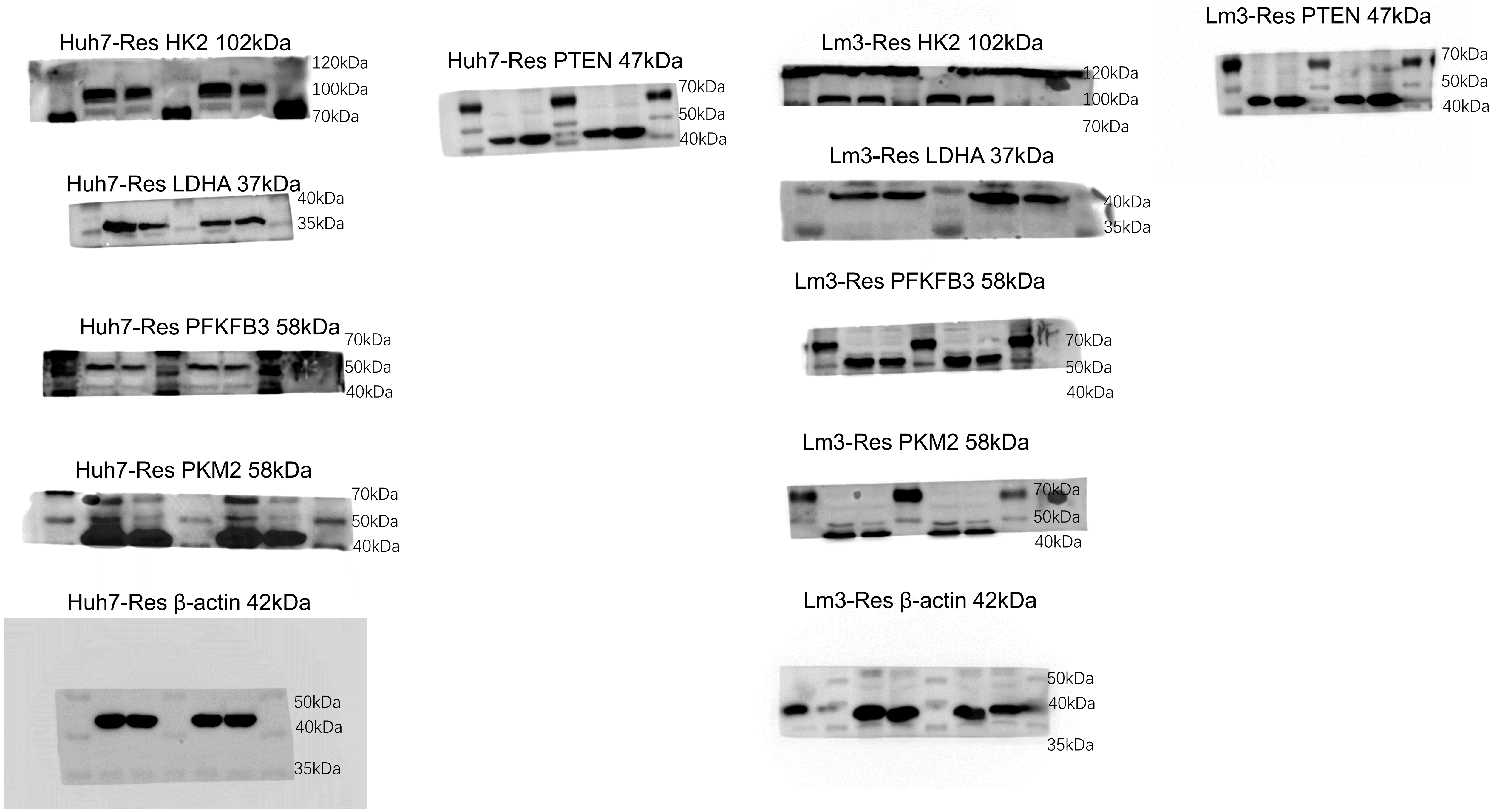
Huh7-Res HK2 102kDa
120kDa
100kDa
70kDa
Huh7-Res PTEN 47kDa
70kDa
50kDa
40kDa
Lm3-Res HK2 102kDa
120kDa
100kDa
70kDa
Lm3-Res PTEN 47kDa
70kDa
50kDa
40kDa
Huh7-Res LDHA 37kDa
40kDa
35kDa
Lm3-Res LDHA 37kDa
40kDa
35kDa
Huh7-Res PFKFB3 58kDa
70kDa
50kDa
40kDa
Lm3-Res PFKFB3 58kDa
70kDa
50kDa
40kDa
Huh7-Res PKM2 58kDa
70kDa
50kDa
40kDa
Lm3-Res PKM2 58kDa
70kDa
50kDa
40kDa
Huh7-Res β-actin 42kDa
50kDa
40kDa
35kDa
Lm3-Res β-actin 42kDa
50kDa
40kDa
35kDa

Huh7-Res HK2 102kDa

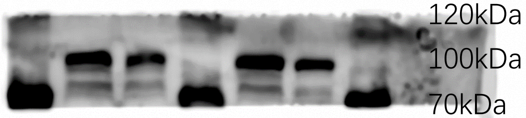

Huh7-Res β-actin 42kDa

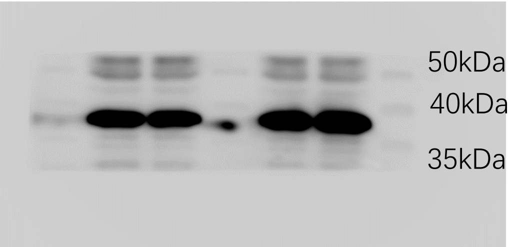

Huh7-Res LDHA 37kDa

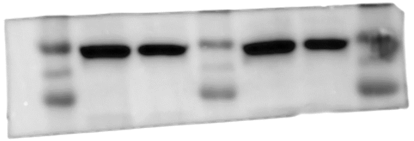

Huh7-Res PFKFB3 58kDa

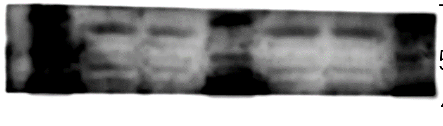

Huh7-Res PKM2 58kDa

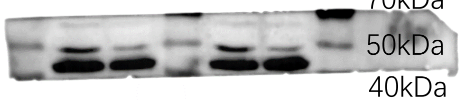

Huh7-Res PTEN 47kDa

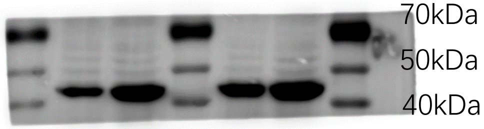

Lm3-Res HK2 102kDa

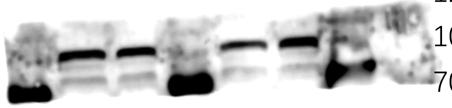

Lm3-Res β-actin 42kDa

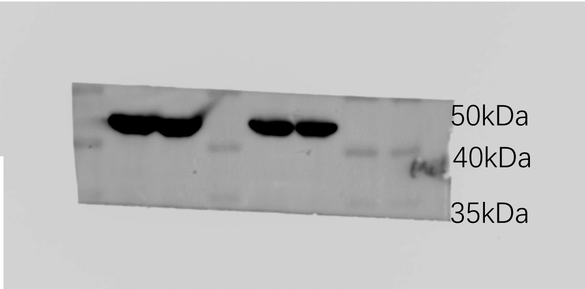

Lm3-Res LDHA 37kDa

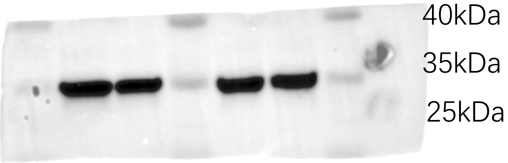

Lm3-Res PFKFB3 58kDa

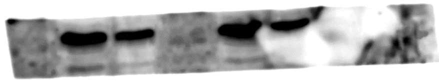

Lm3-Res PKM2 58kDa

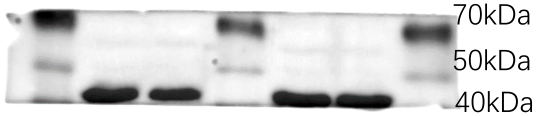

Lm3-Res PTEN 47kDa

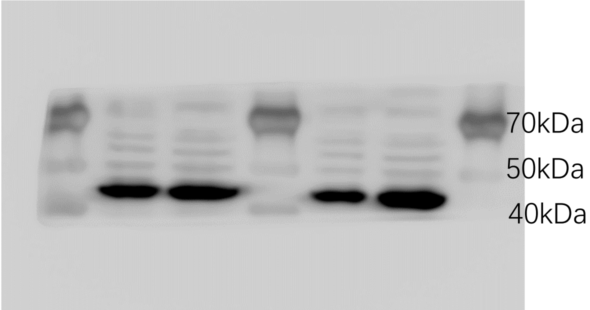

Figure.7F

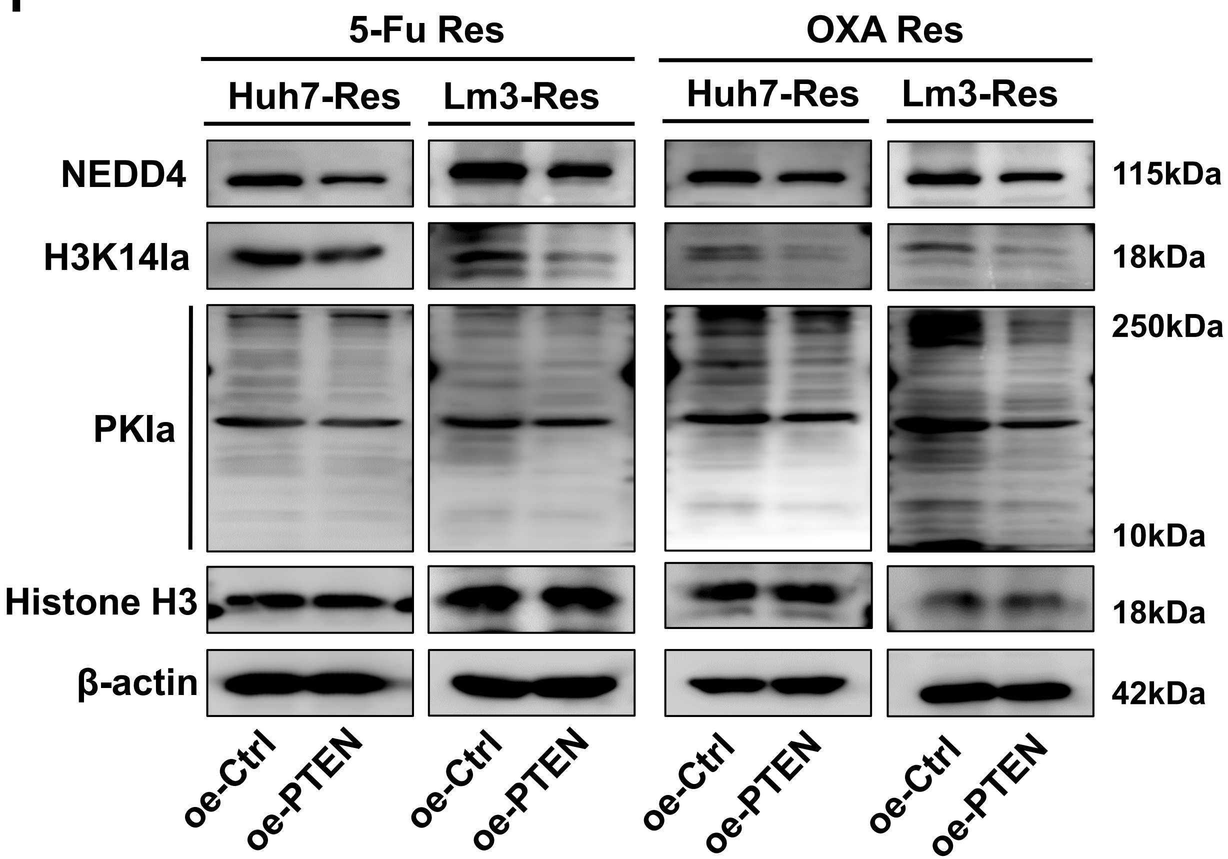
5-Fu Res
OXA Res
Huh7-Res
Lm3-Res
Huh7-Res
Lm3-Res
NEDD4
H3K14la
PKla
Histone H3
β-actin
115kDa
18kDa
250kDa
10kDa
18kDa
42kDa
oe-Ctrl
oe-PTEN
oe-Ctrl
oe-PTEN
oe-Ctrl
oe-PTEN
oe-Ctrl
oe-PTEN

Huh7-Res NEDD4 115kDa

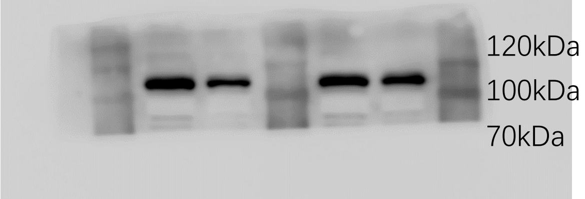

Huh7-Res β-actin 42kDa

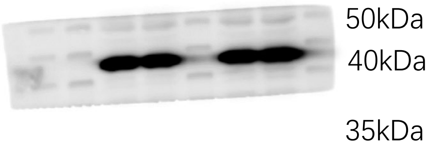

Lm3-Res NEDD4 115kDa

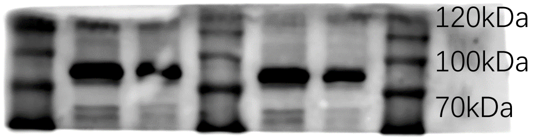

Lm3-Res β-actin 42kDa

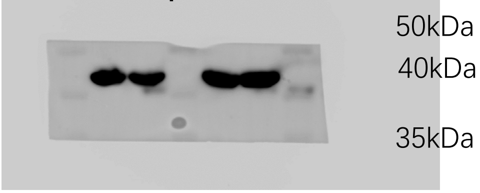

Huh7-Res H3K14la 18kDa

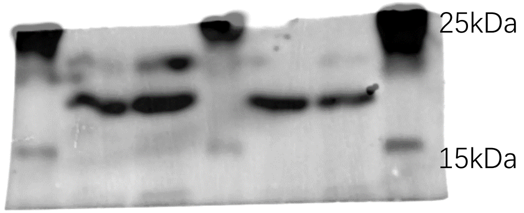

Lm3-Res H3K14la 18kDa

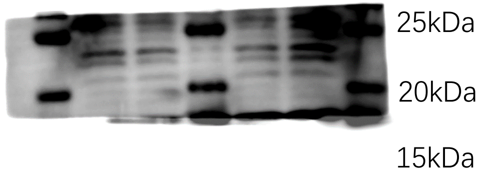

Huh7-Res Pkla

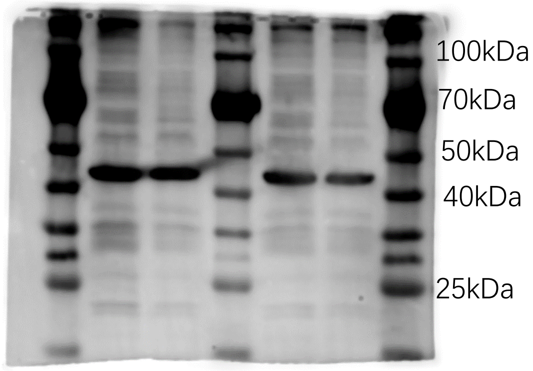

Lm3-Res Pkla

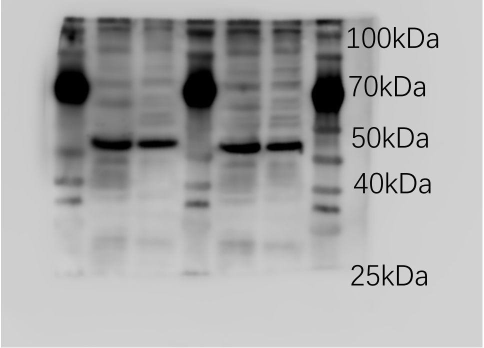

Huh7-Res Histone H3 18kDa

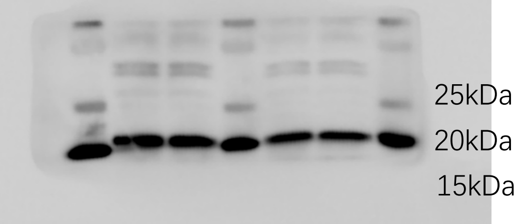

Lm3-Res Histone H3 18kDa

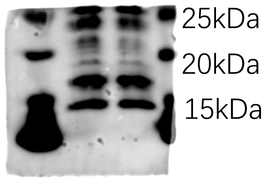

Huh7-Res NEDD4 115kDa

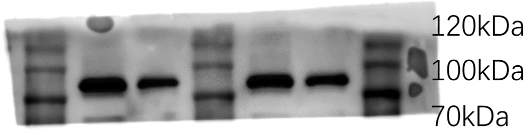

Huh7-Res β-actin 42kDa

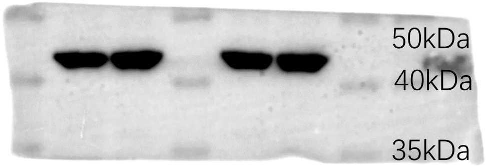

Lm3-Res NEDD4 115kDa

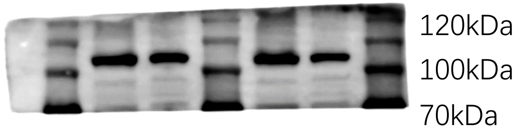

Lm3-Res β-actin 42kDa

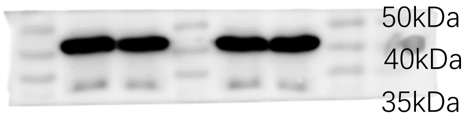

Huh7-Res H3K14la 18kDa

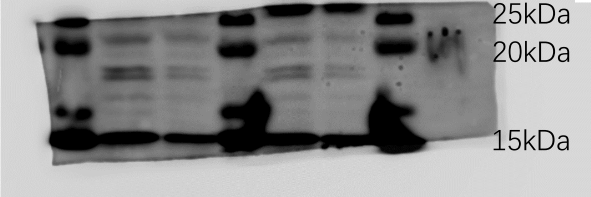

Lm3-Res H3K14la 18kDa

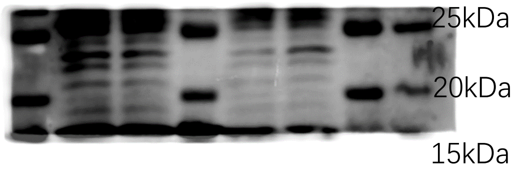

Huh7-Res Pkla

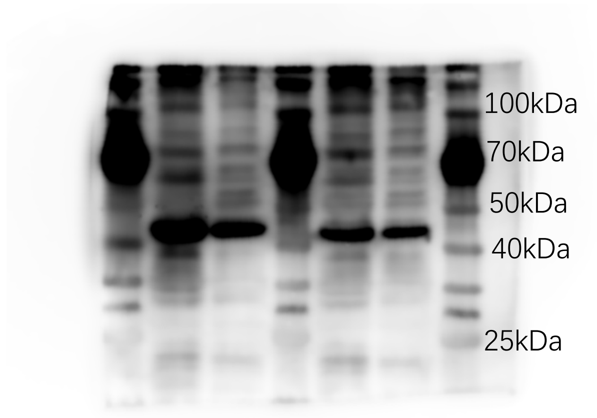

Lm3-Res Pkla

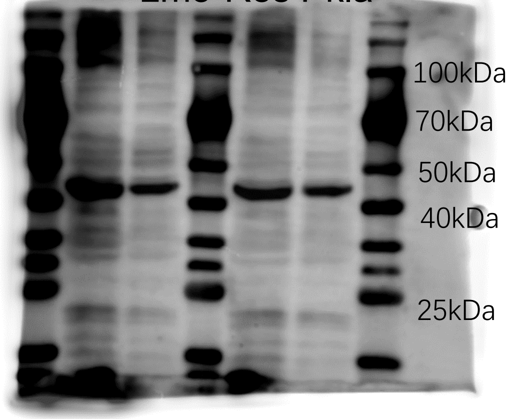

Huh7-Res Histone H3 18kDa

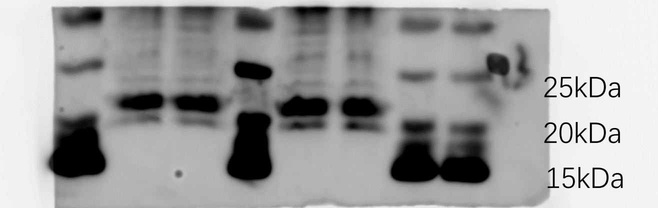

Lm3-Res Histone H3 18kDa

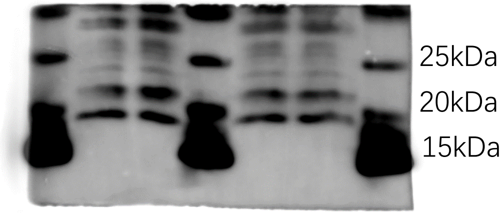

Figure.8D-G

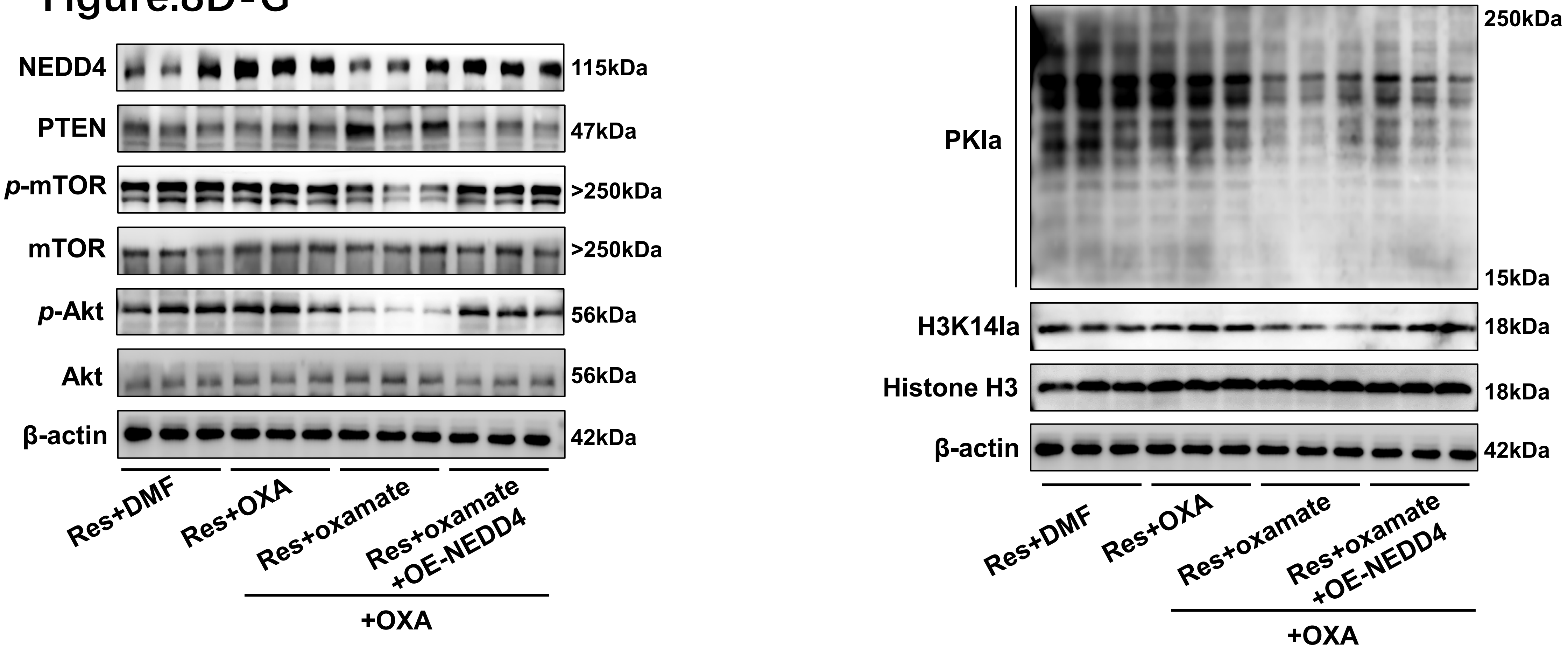
NEDD4
PTEN
p-mTOR
mTOR
p-Akt
Akt
β-actin
115kDa
47kDa
>250kDa
>250kDa
56kDa
56kDa
42kDa
Res+DMF
Res+OXA
Res+oxamate
Res+oxamate +OE-NEDD4
+OXA
PKla
H3K14la
Histone H3
β-actin
250kDa
15kDa
18kDa
18kDa
42kDa
Res+DMF
Res+OXA
Res+oxamate
Res+oxamate +OE-NEDD4
+OXA

NEDD4 115kDa

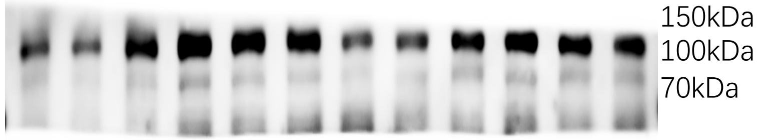

PTEN 47kDa

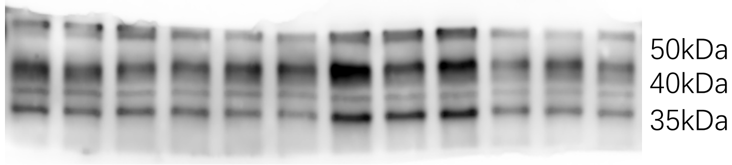

p-mTOR 250kDa

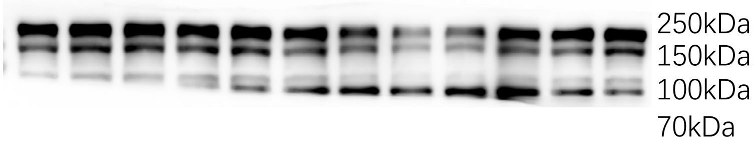

mTOR 250kDa

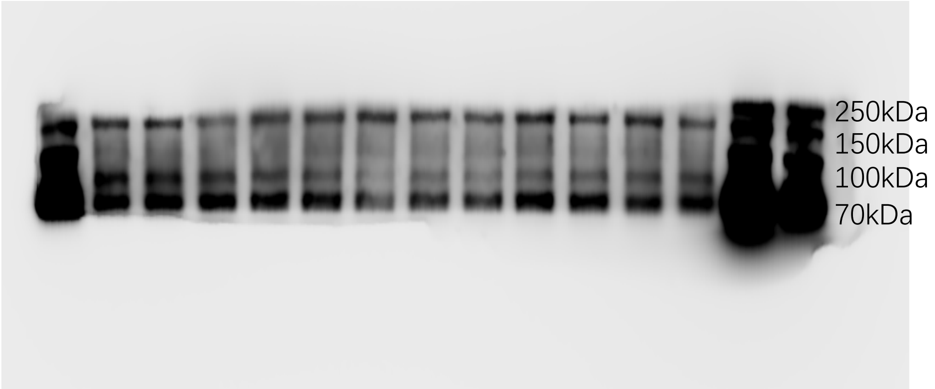

p-Akt 56kDa

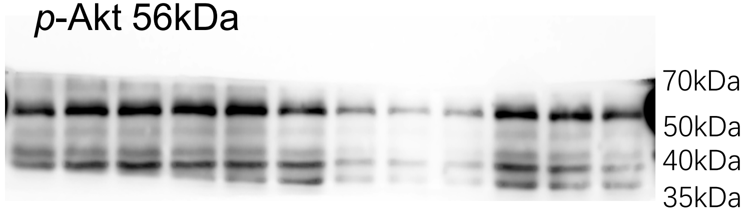

Akt 56kDa

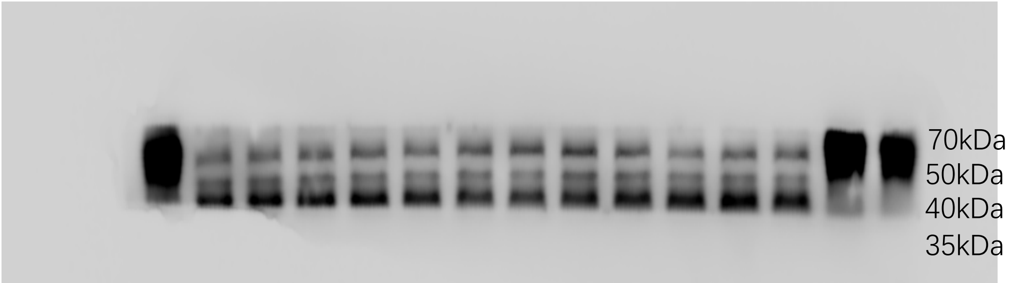

β-actin 42kDa

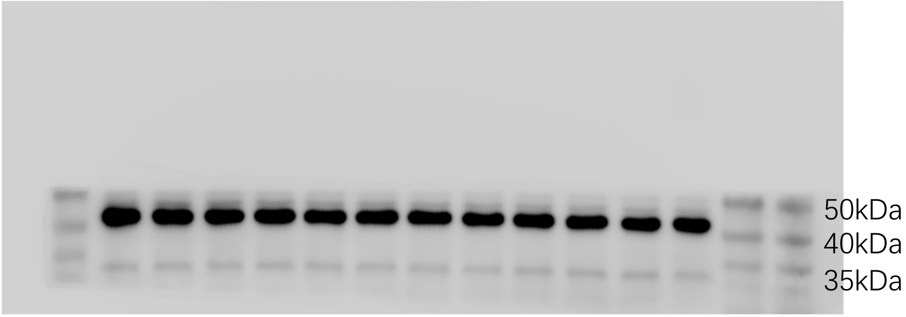

Pkla 10-250kDa

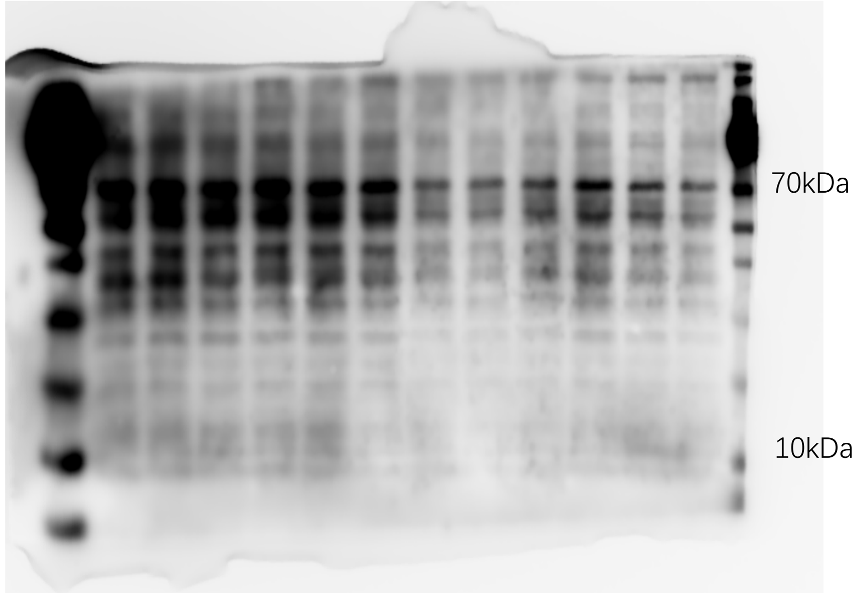

H3K14la 18kDa

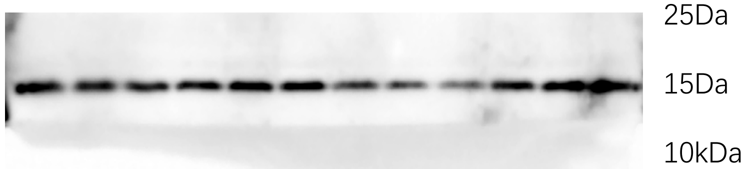

Histone H3 18kDa

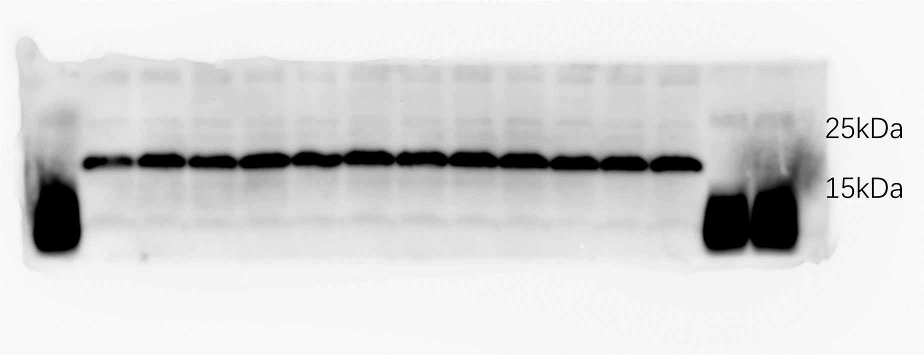

β-actin 42kDa

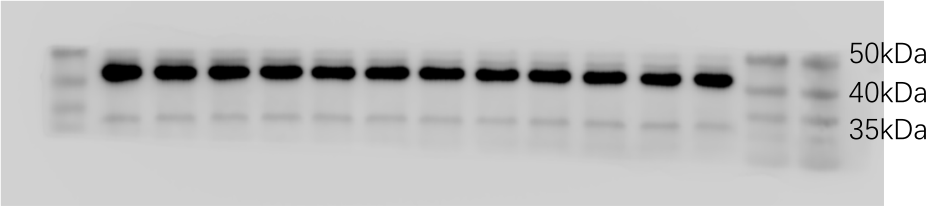

S1.D

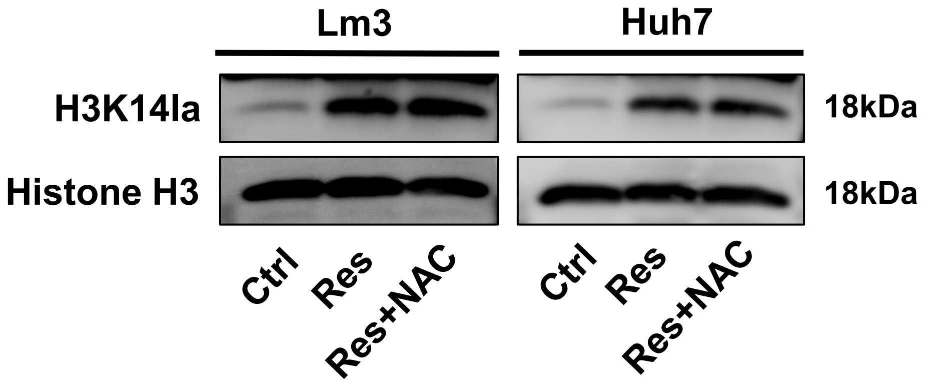
Lm3
Huh7
H3K14la
Histone H3
18kDa
18kDa
Ctrl
Res
Res+NAC
Ctrl
Res
Res+NAC

H3K14la 18kDa

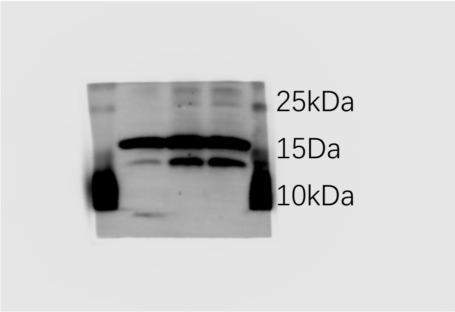

H3K14la 18kDa

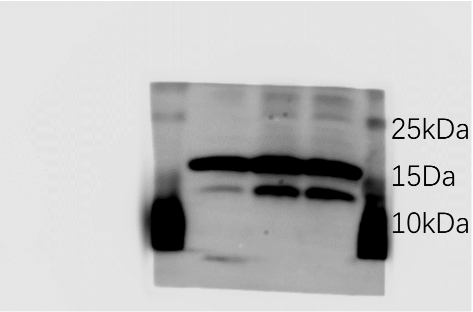

Histone H3 18kDa

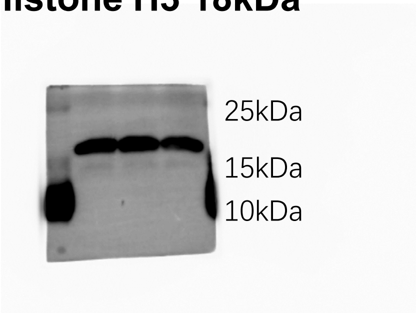

Histone H3 18kDa

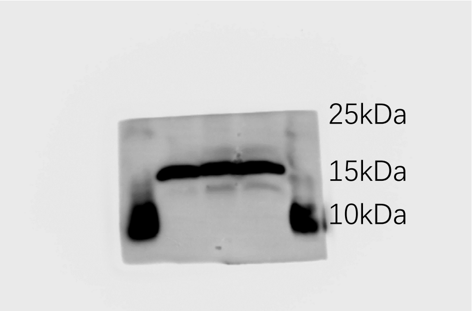

S2.C

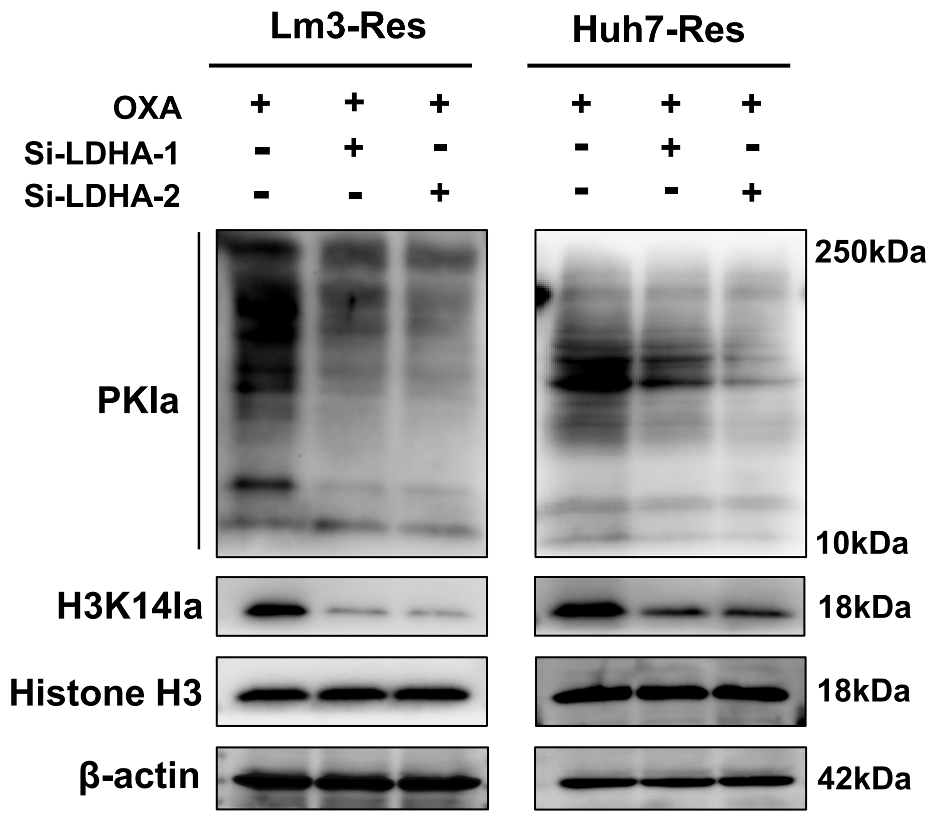
Lm3-Res
Huh7-Res
OXA
Si-LDHA-1
Si-LDHA-2
PKla
H3K14la
Histone H3
β-actin
250kDa
10kDa
18kDa
18kDa
42kDa

Lm3-Res

Pkla 10-250kDa

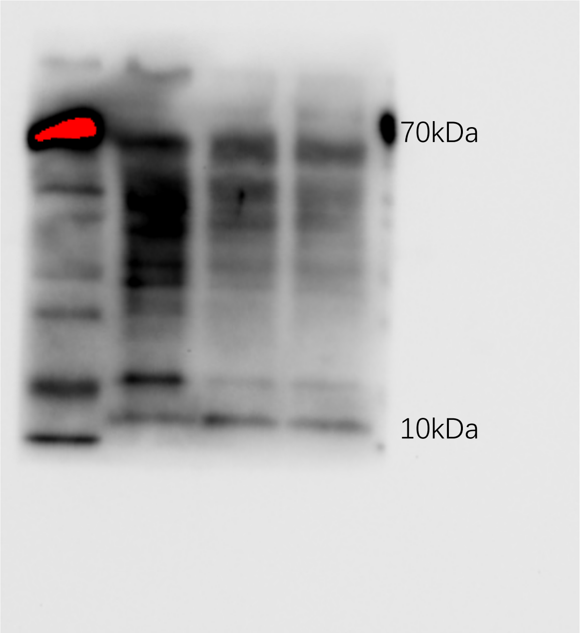

H3K14la 18kDa

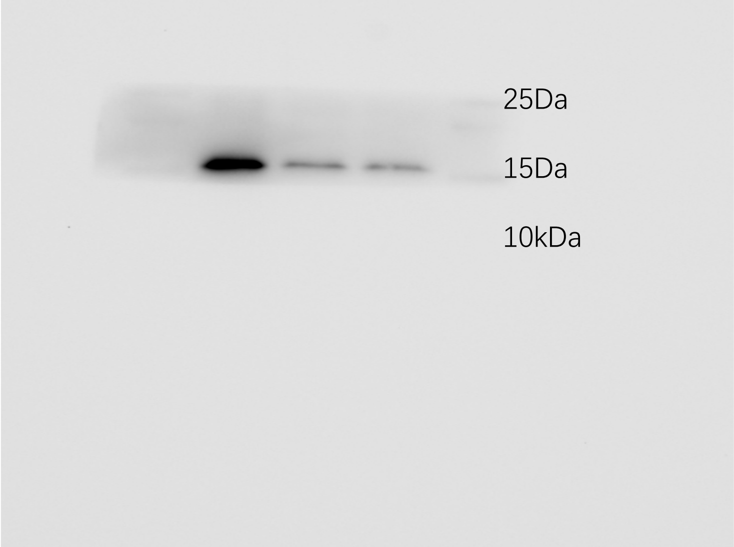

Histone H3 18kDa

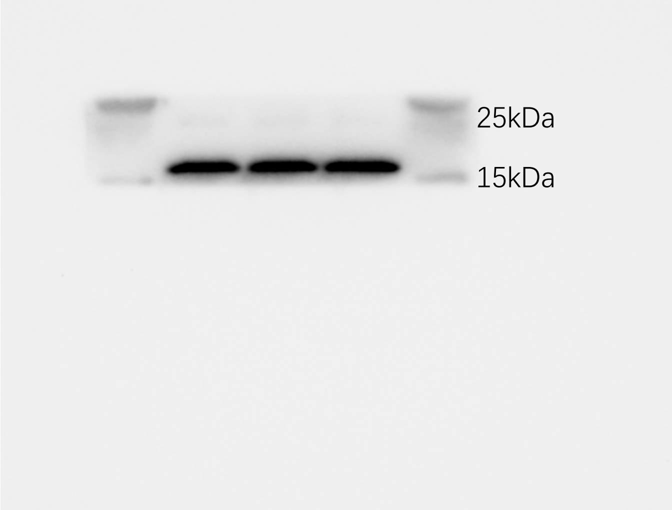

β-actin 42kDa

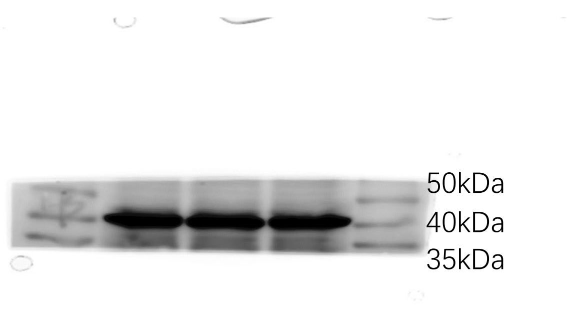

Lm3-Res

Pkla 10-250kDa

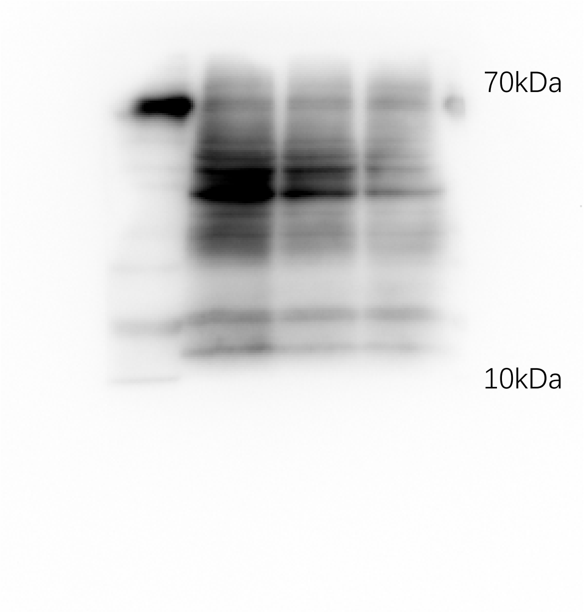

H3K14la 18kDa

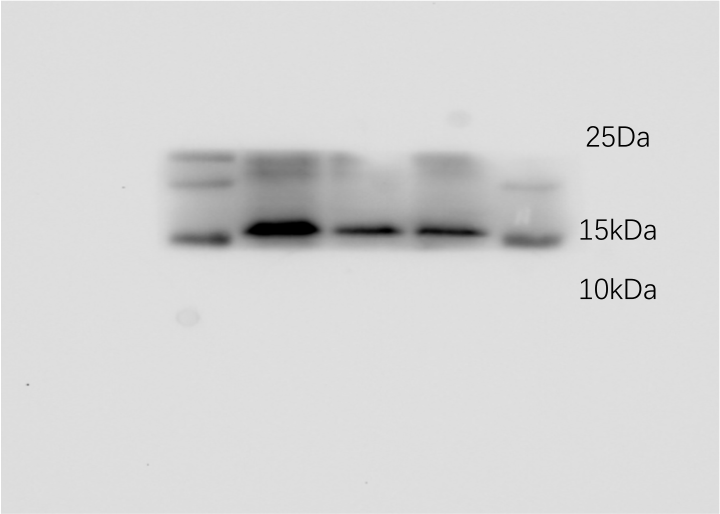

Histone H3 18kDa

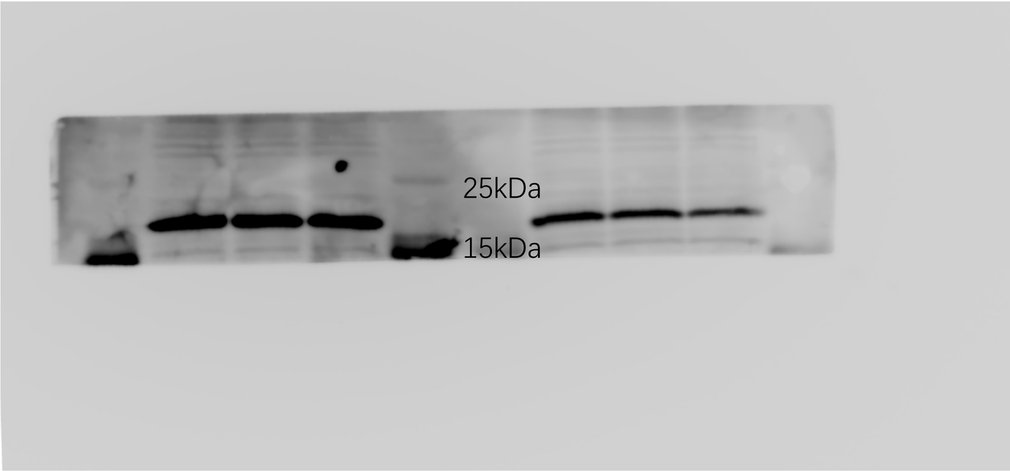

β-actin 42kDa

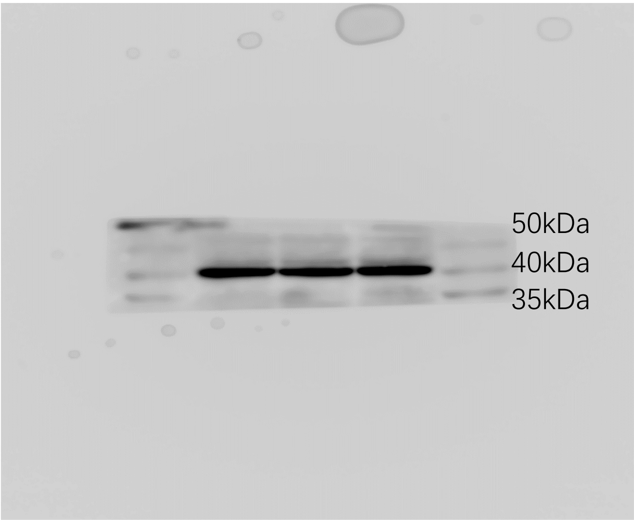

S3.C

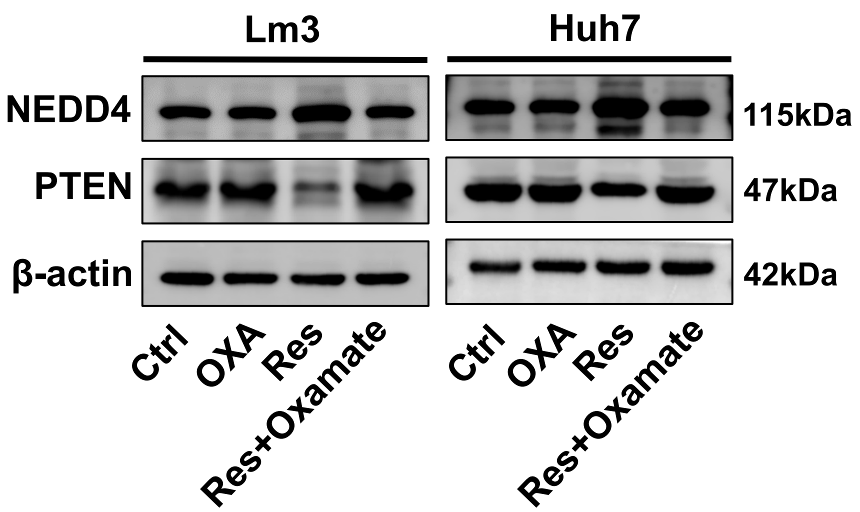
Lm3
Huh7
NEDD4
PTEN
β-actin
115kDa
47kDa
42kDa
Ctrl
OXA
Res
Res+Oxamate
Ctrl
OXA
Res
Res+Oxamate

LM3 NEDD4

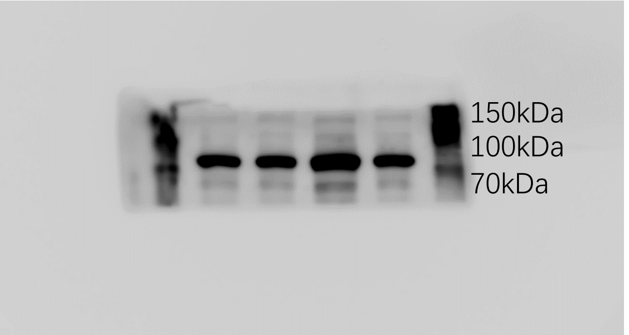

LM3 PTEN

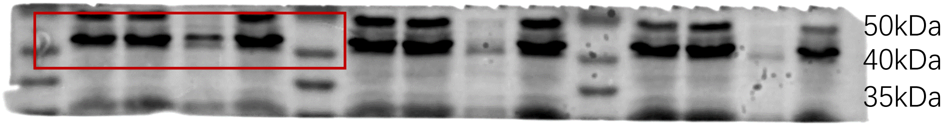

LM3 ACTB

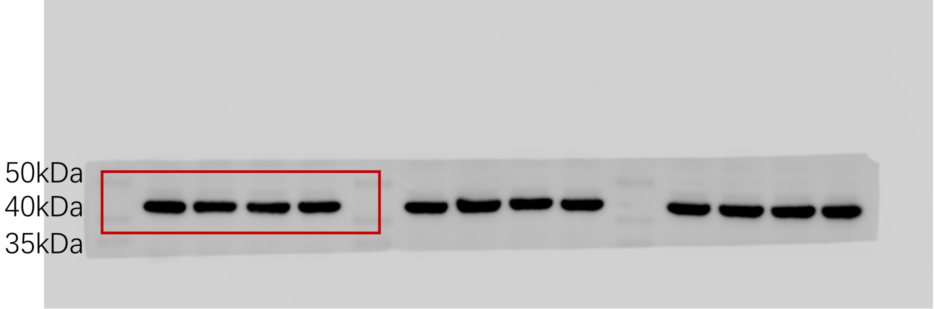

huh7 NEDD4

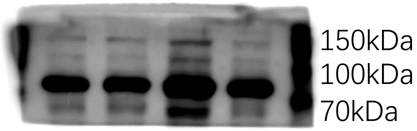

huh7 PTEN

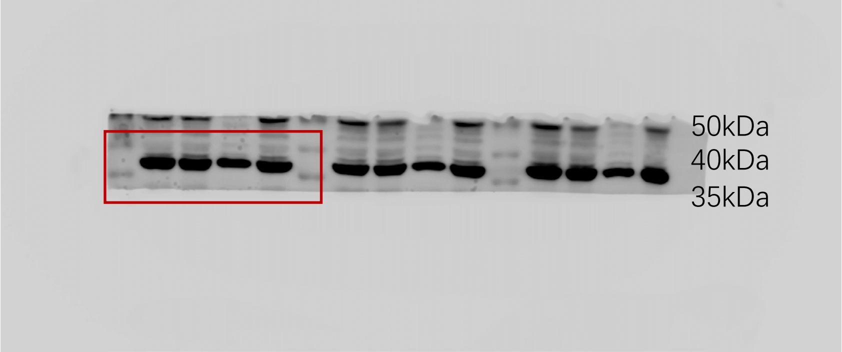

huh7 ACTB

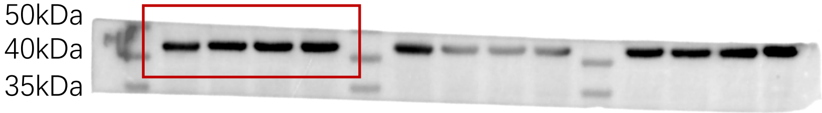

S3.D

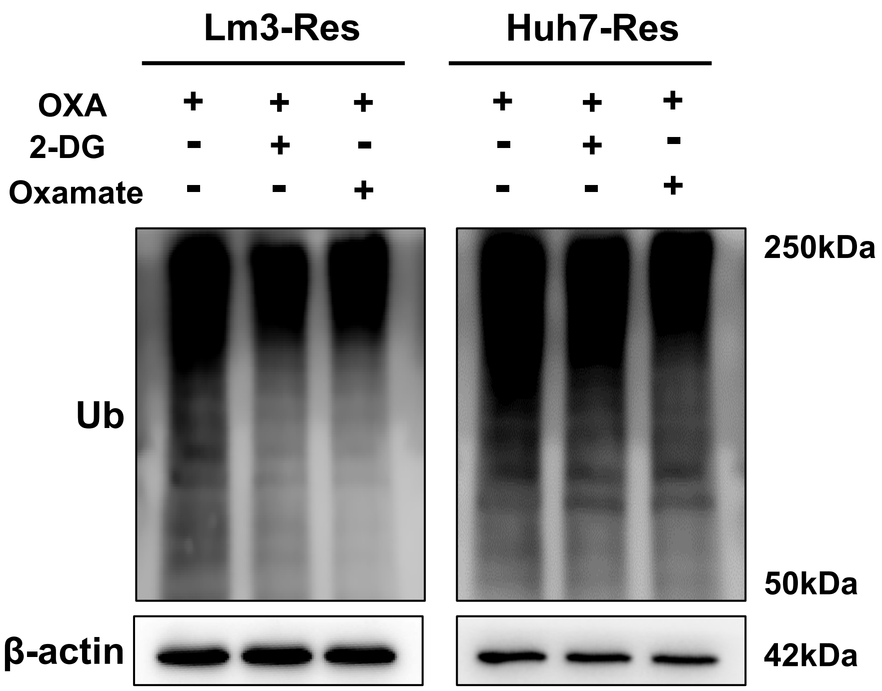
Lm3-Res
Huh7-Res
OXA + + + + + +
2-DG - + - - + -
Oxamate - - + - - +
Ub
250kDa
50kDa
β-actin
42kDa

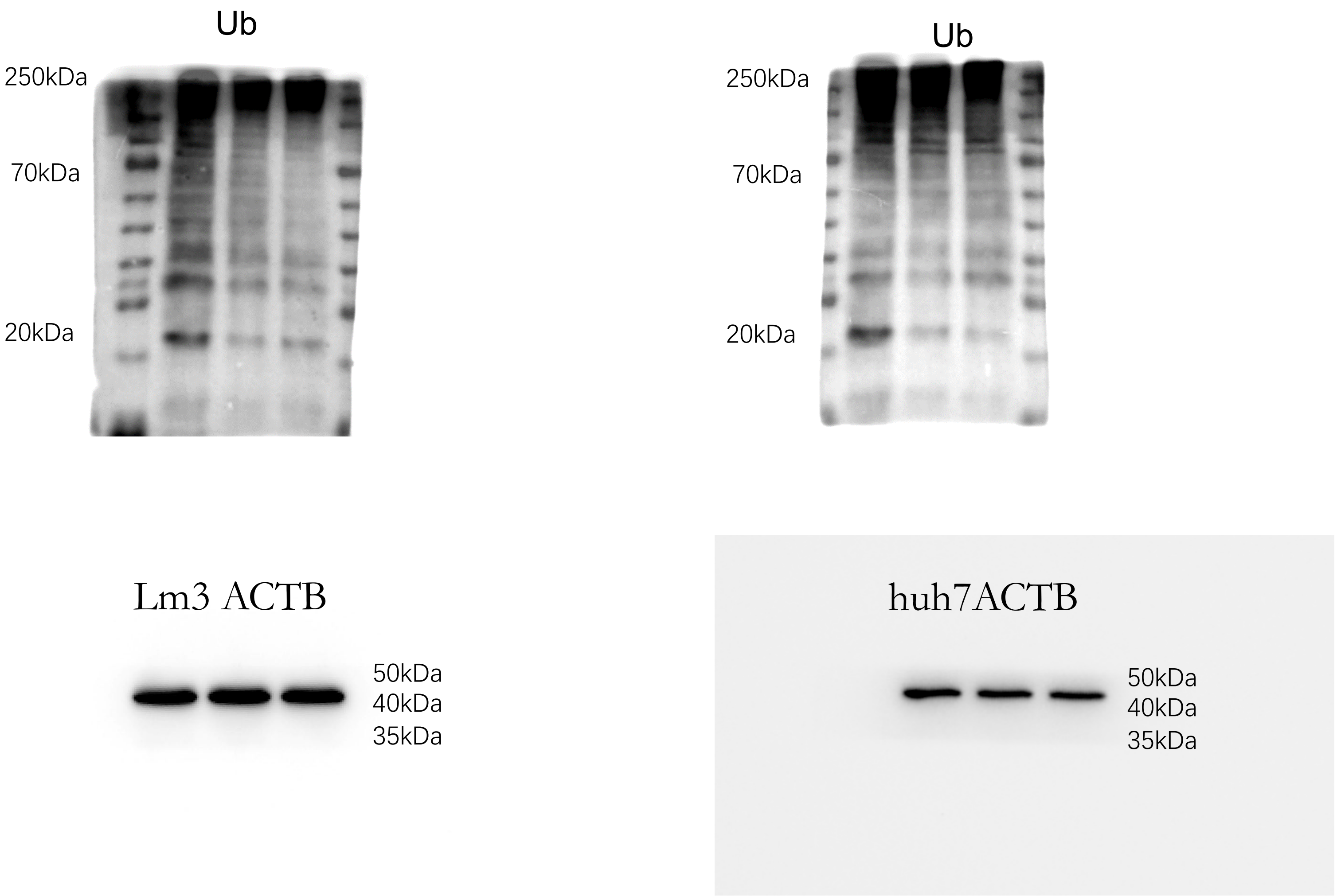
Ub
250kDa
70kDa
20kDa
Ub
250kDa
70kDa
20kDa
Lm3 ACTB
50kDa
40kDa
35kDa
huh7ACTB
50kDa
40kDa
35kDa

S3.F

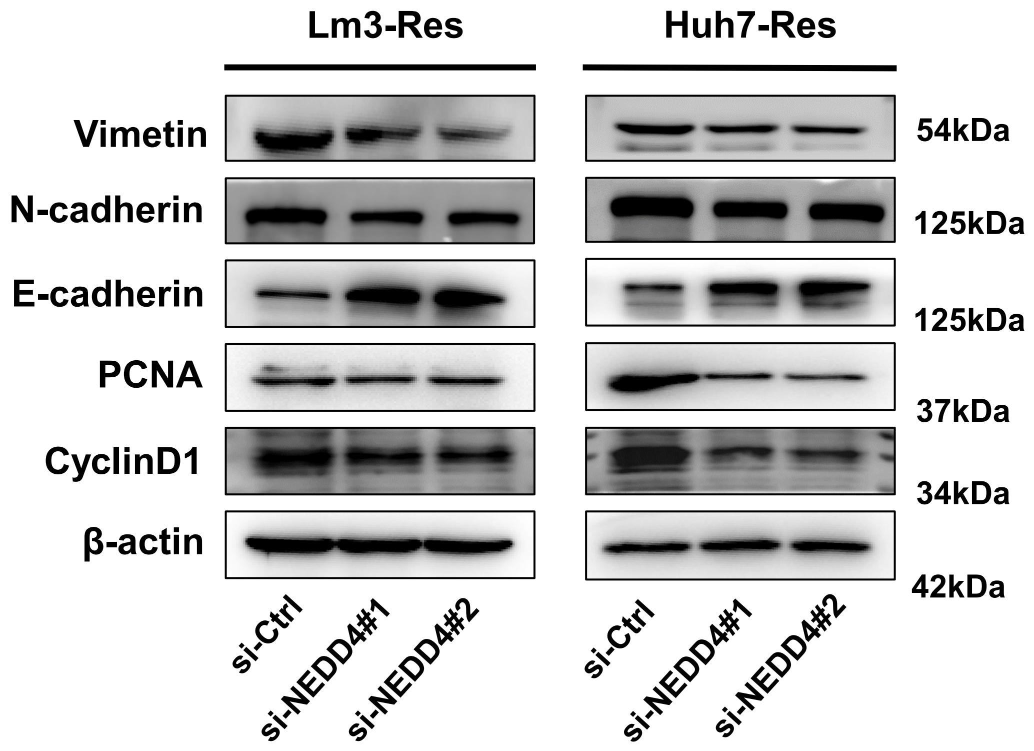
Lm3-Res
Huh7-Res
Vimetin
N-cadherin
E-cadherin
PCNA
CyclinD1
β-actin
54kDa
125kDa
125kDa
37kDa
34kDa
42kDa
si-Ctrl
si-NEDD4#1
si-NEDD4#2
si-Ctrl
si-NEDD4#1
si-NEDD4#2

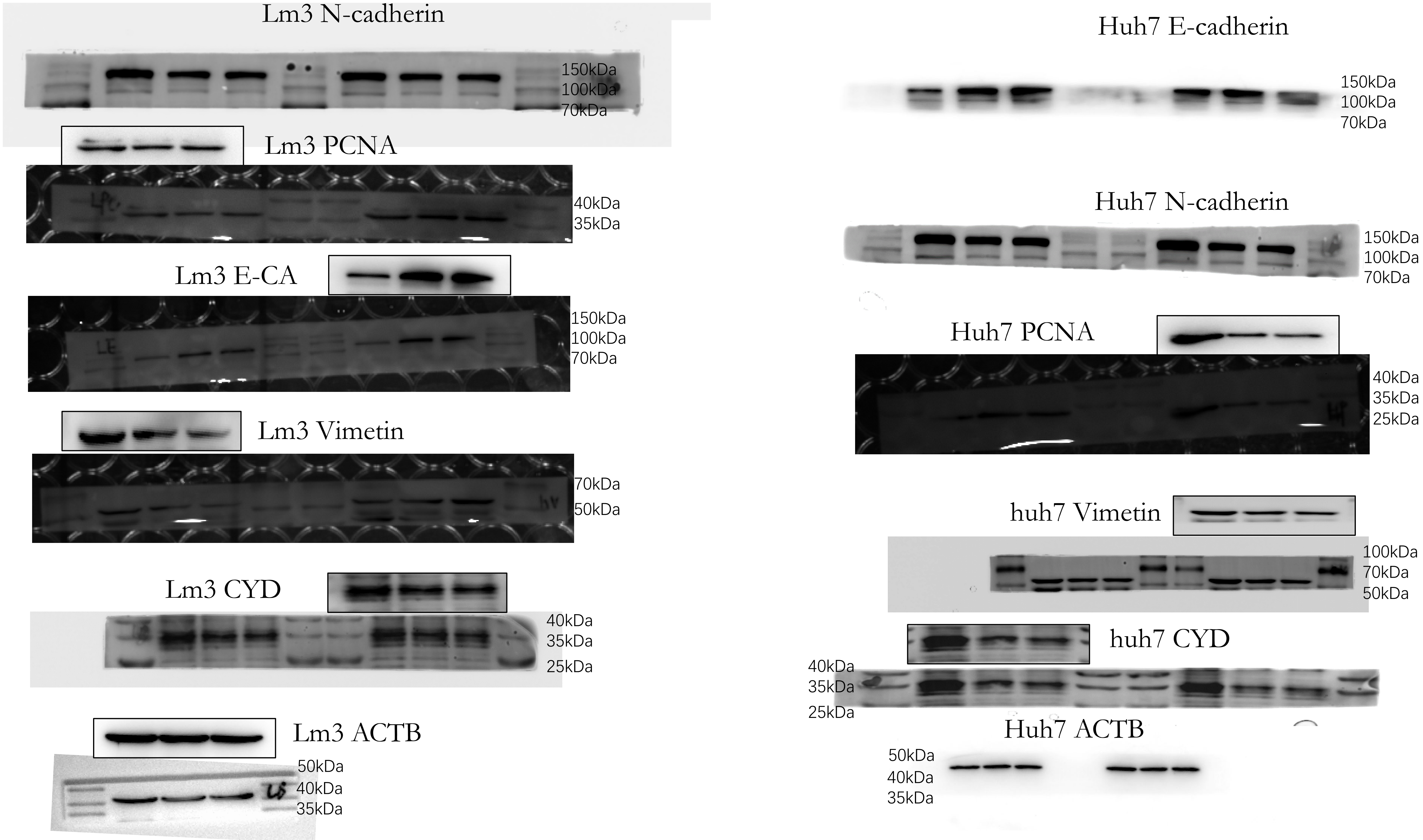
Lm3 N-cadherin
150kDa
100kDa
70kDa
Lm3 PCNA
40kDa
35kDa
Lm3 E-CA
150kDa
100kDa
70kDa
Lm3 Vimetin
70kDa
50kDa
Lm3 CYD
40kDa
35kDa
25kDa
Lm3 ACTB
50kDa
40kDa
35kDa
Huh7 E-cadherin
150kDa
100kDa
70kDa
Huh7 N-cadherin
150kDa
100kDa
70kDa
Huh7 PCNA
40kDa
35kDa
25kDa
huh7 Vimetin
100kDa
70kDa
50kDa
huh7 CYD
40kDa
35kDa
25kDa
Huh7 ACTB
50kDa
40kDa
35kDa

S5.A

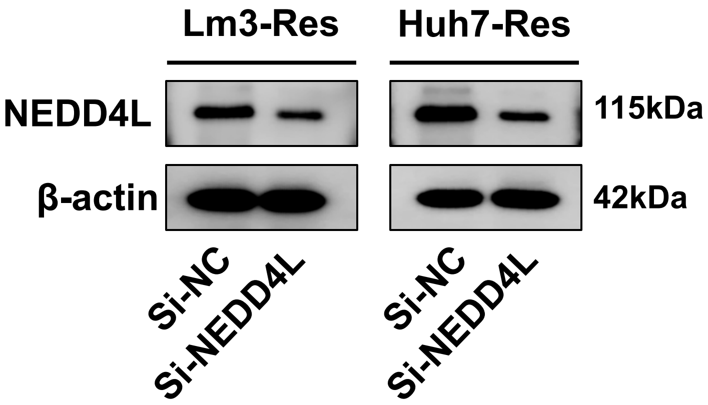

Lm3-Res
Huh7-Res
NEDD4L
β-actin
115kDa
42kDa
Si-NC
Si-NEDD4L
Si-NC
Si-NEDD4L

NEDD4L 112kDa

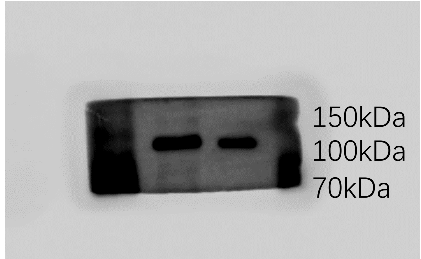

NEDD4L 112kDa

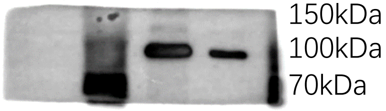

β-actin 42kDa

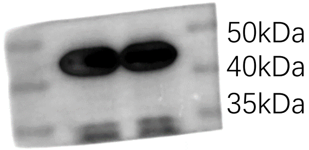

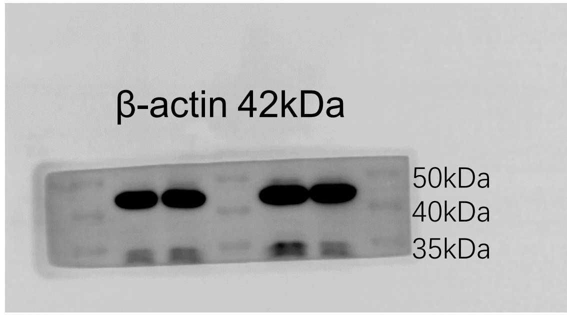

S5.B

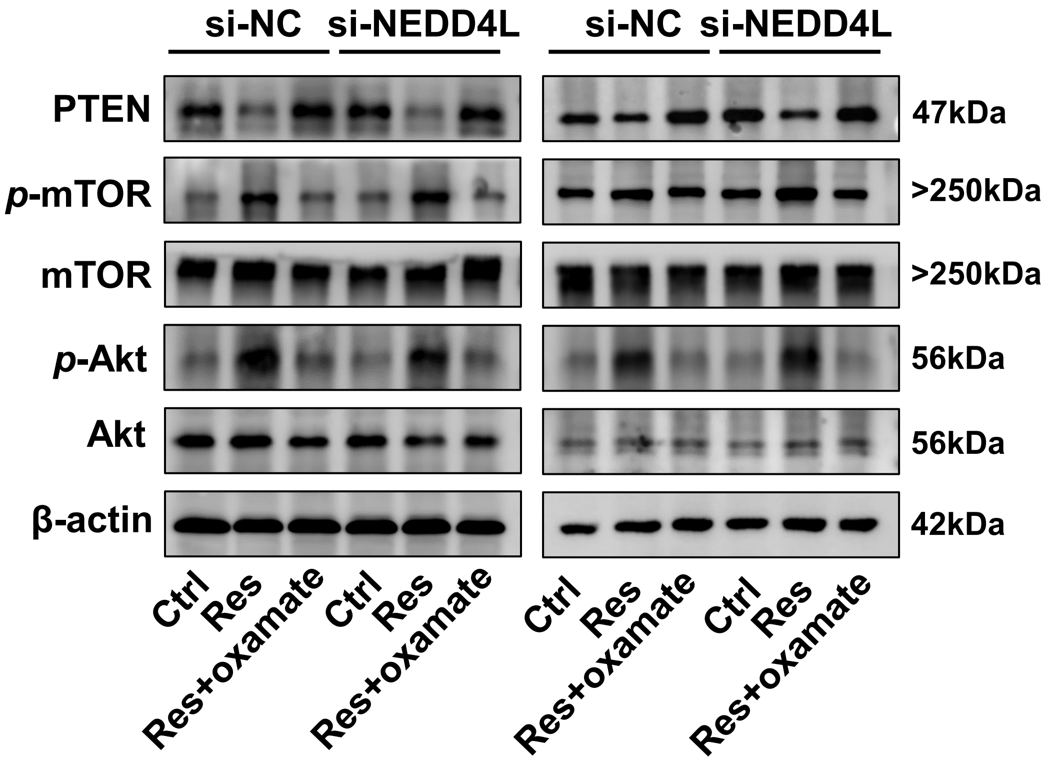
si-NC
si-NEDD4L
si-NC
si-NEDD4L
PTEN
p-mTOR
mTOR
p-Akt
Akt
β-actin
47kDa
>250kDa
>250kDa
56kDa
56kDa
42kDa
Ctrl
Res
Res+oxamate
Ctrl
Res
Res+oxamate
Ctrl
Res
Res+oxamate
Ctrl
Res
Res+oxamate

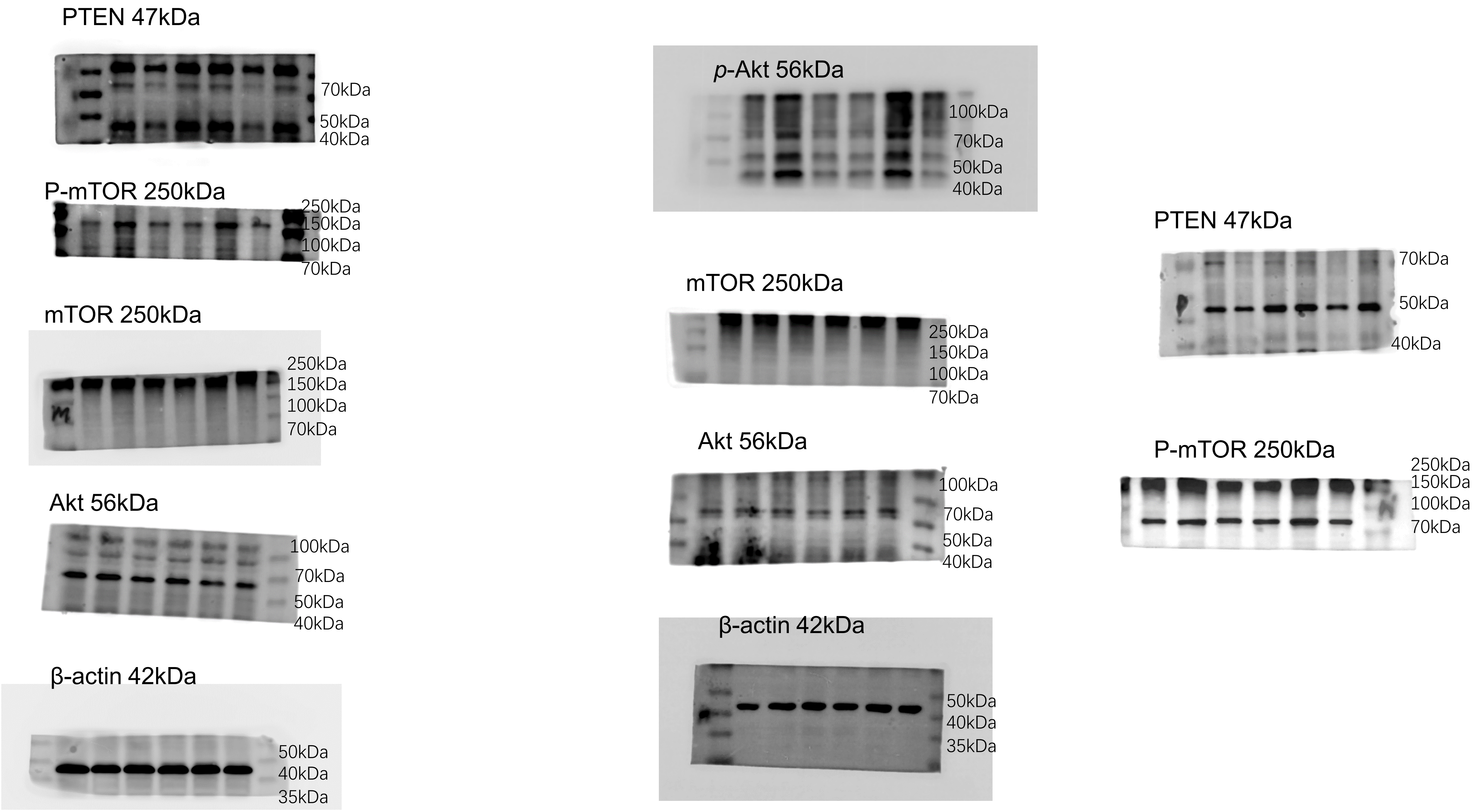
PTEN 47kDa
70kDa
50kDa
40kDa
P-mTOR 250kDa
250kDa
150kDa
100kDa
70kDa
mTOR 250kDa
250kDa
150kDa
100kDa
70kDa
Akt 56kDa
100kDa
70kDa
50kDa
40kDa
β-actin 42kDa
50kDa
40kDa
35kDa
p-Akt 56kDa
100kDa
70kDa
50kDa
40kDa
mTOR 250kDa
250kDa
150kDa
100kDa
70kDa
Akt 56kDa
100kDa
70kDa
50kDa
40kDa
β-actin 42kDa
50kDa
40kDa
35kDa
PTEN 47kDa
70kDa
50kDa
40kDa
P-mTOR 250kDa
250kDa
150kDa
100kDa
70kDa

S5.C

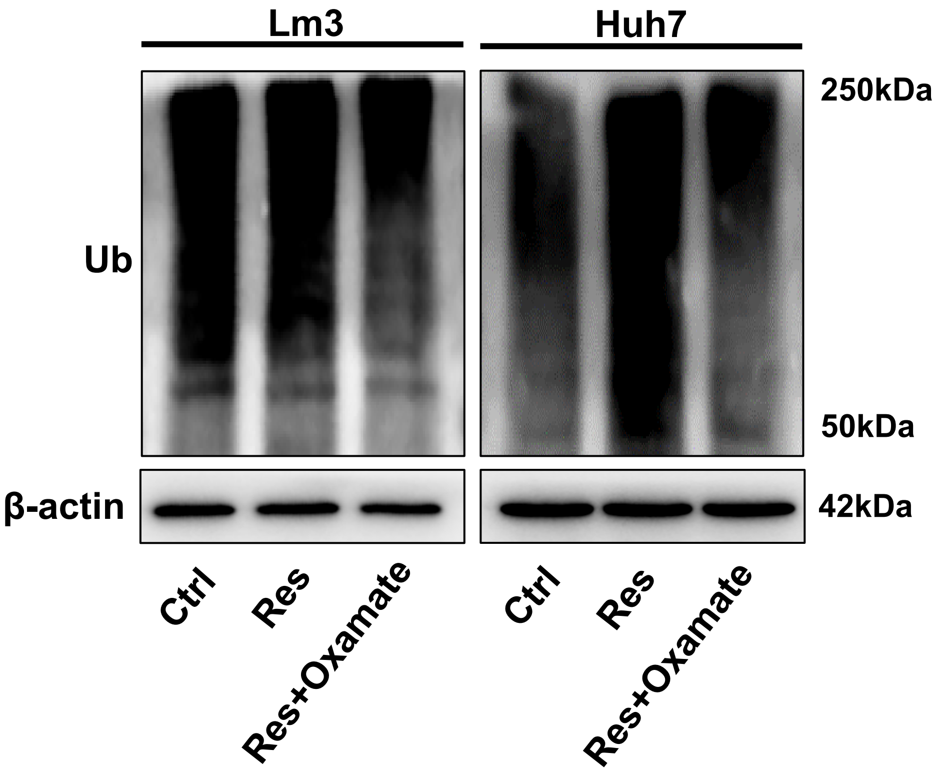
Lm3
Huh7
250kDa
Ub
50kDa
β-actin
42kDa
Ctrl
Res
Res+Oxamate
Ctrl
Res
Res+Oxamate

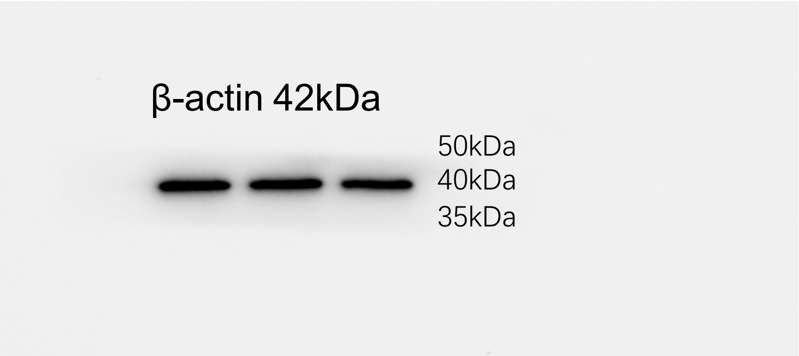
β-actin 42kDa
50kDa
40kDa
35kDa

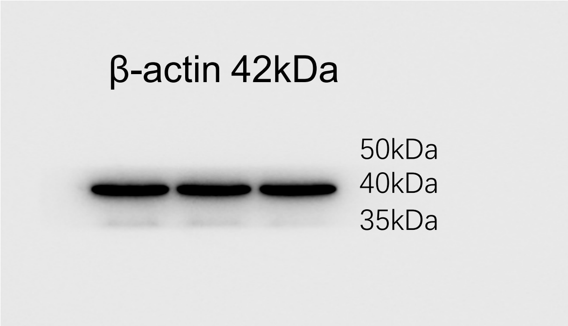
β-actin 42kDa
50kDa
40kDa
35kDa

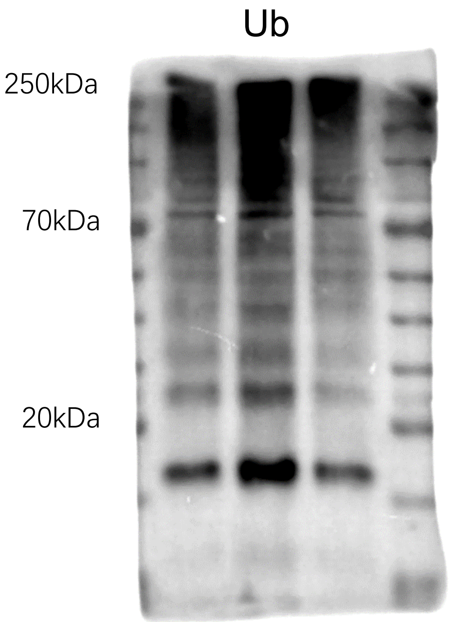
Ub
250kDa
70kDa
20kDa

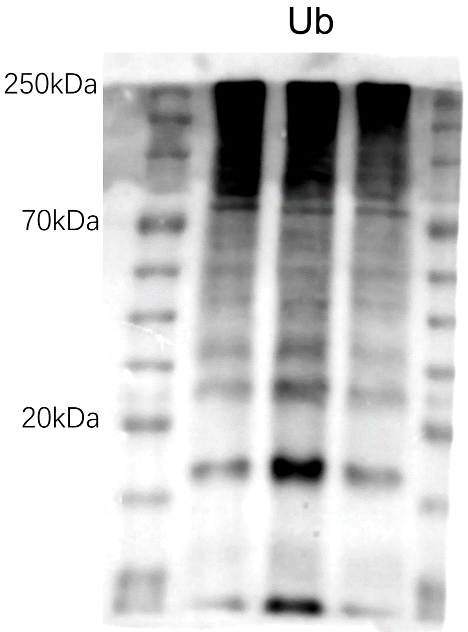
Ub
250kDa
70kDa
20kDa

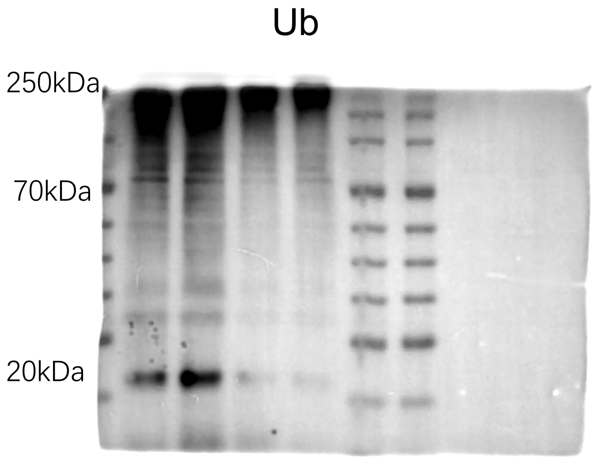
Ub
250kDa
70kDa
20kDa

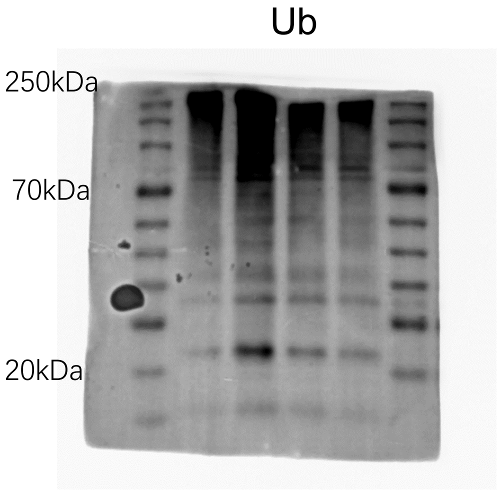
Ub
250kDa
70kDa
20kDa

S6.A

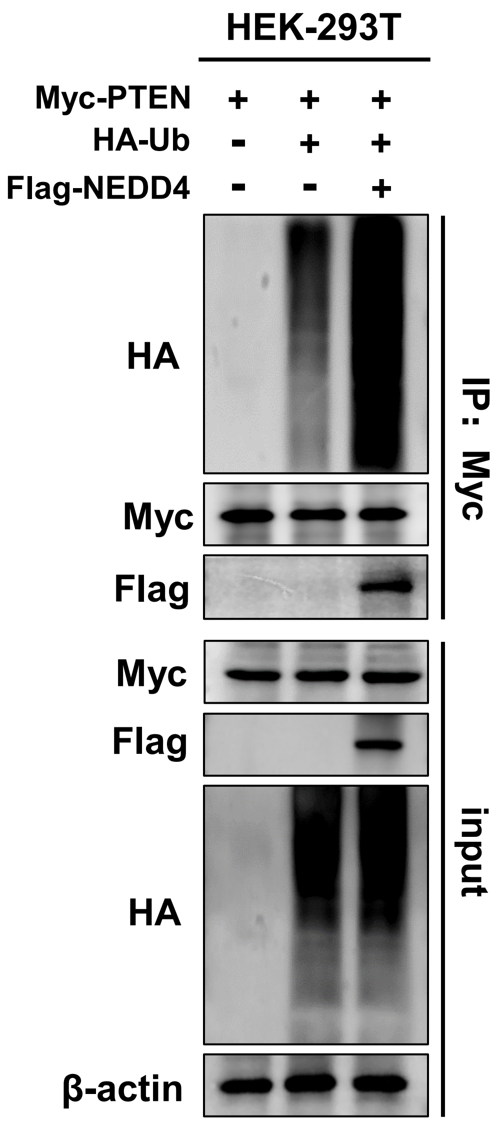
HEK-293T
Myc-PTEN + + +
HA-Ub - + +
Flag-NEDD4 - - +
HA
Myc
Flag
IP: Myc
Myc
Flag
HA
β-actin
input

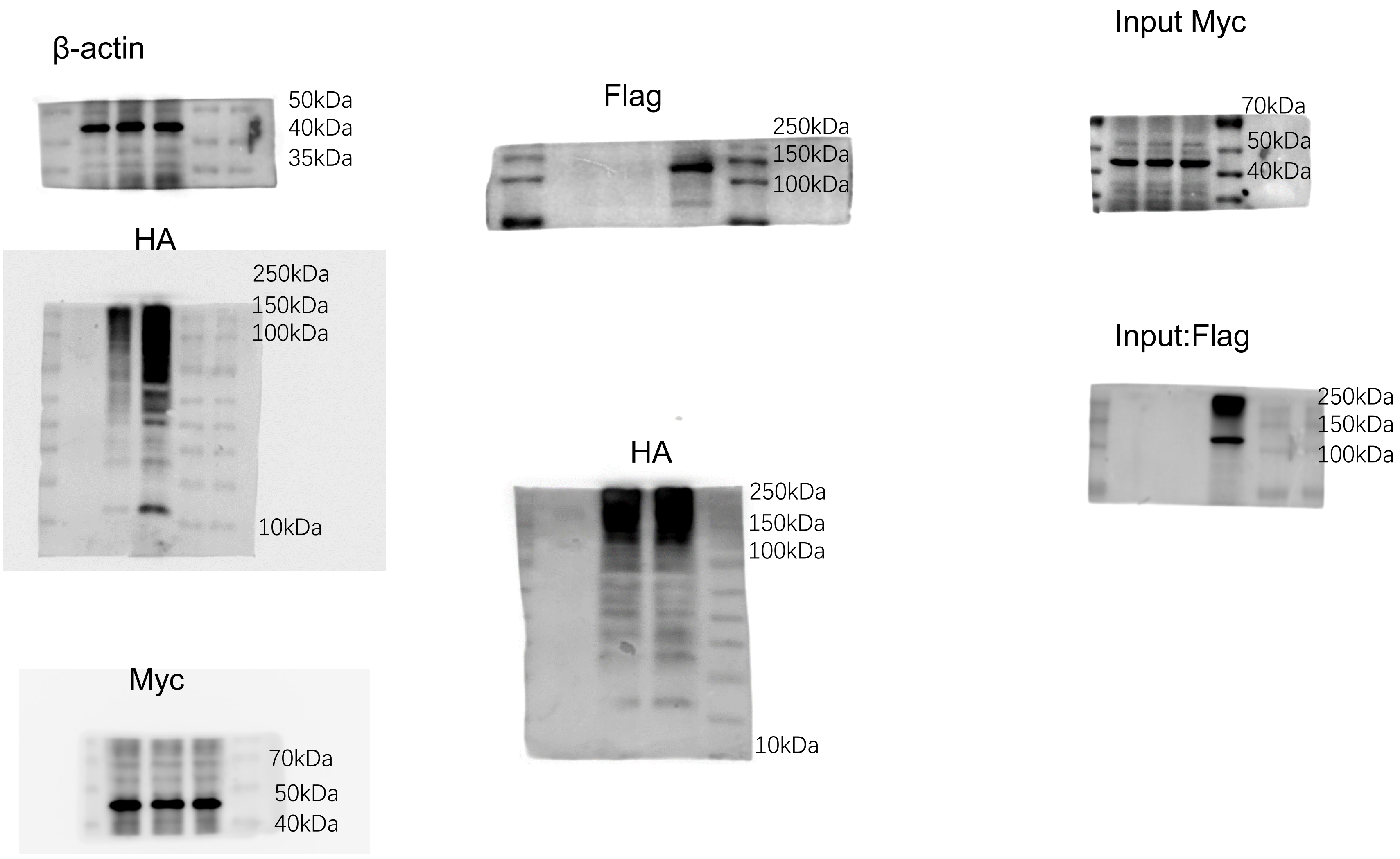
β-actin
50kDa
40kDa
35kDa
HA
250kDa
150kDa
100kDa
10kDa
Myc
70kDa
50kDa
40kDa
Flag
250kDa
150kDa
100kDa
HA
250kDa
150kDa
100kDa
10kDa
Input Myc
70kDa
50kDa
40kDa
Input:Flag
250kDa
150kDa
100kDa

S7.B

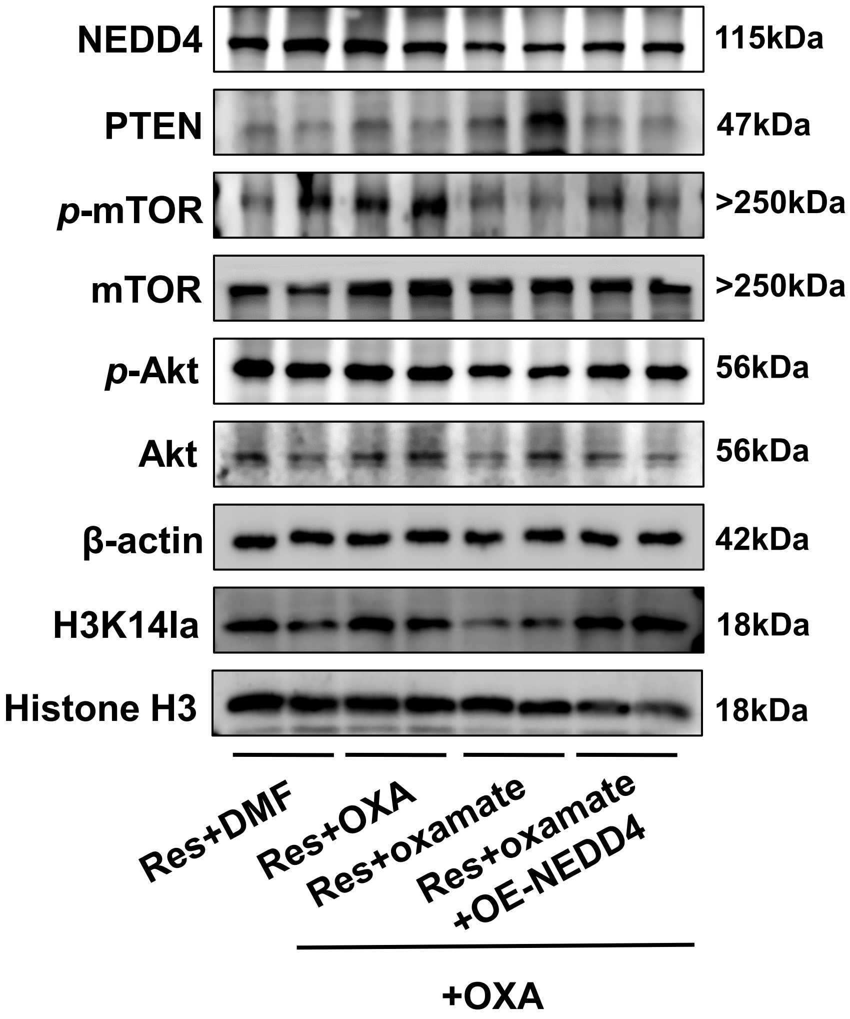
NEDD4
PTEN
p-mTOR
mTOR
p-Akt
Akt
β-actin
H3K14la
Histone H3
115kDa
47kDa
>250kDa
>250kDa
56kDa
56kDa
42kDa
18kDa
18kDa
Res+DMF
Res+OXA
Res+oxamate
Res+oxamate
+OE-NEDD4
+OXA

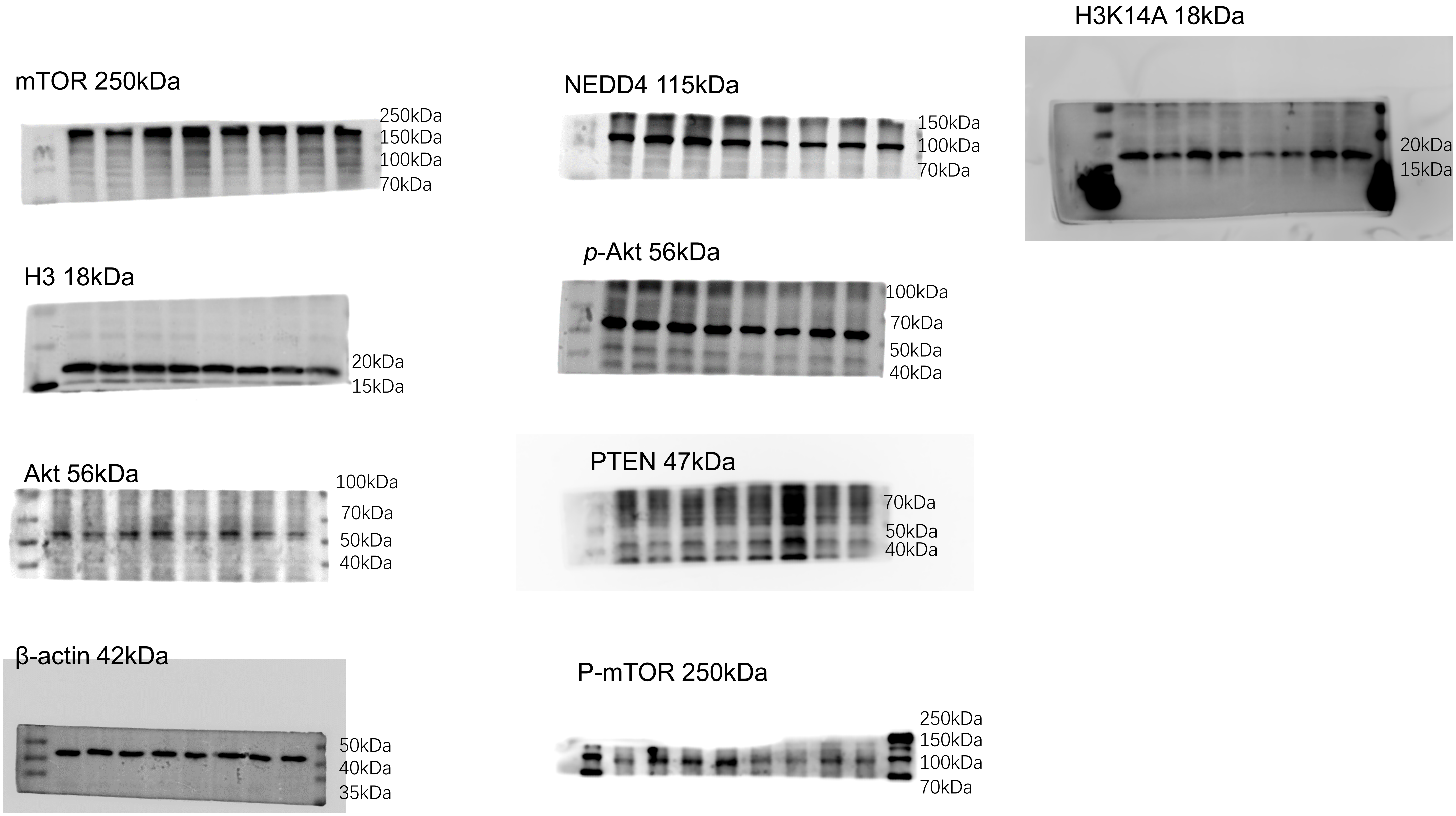
mTOR 250kDa
250kDa
150kDa
100kDa
70kDa
H3 18kDa
20kDa
15kDa
Akt 56kDa
100kDa
70kDa
50kDa
40kDa
β-actin 42kDa
50kDa
40kDa
35kDa
NEDD4 115kDa
150kDa
100kDa
70kDa
p-Akt 56kDa
100kDa
70kDa
50kDa
40kDa
PTEN 47kDa
70kDa
50kDa
40kDa
P-mTOR 250kDa
250kDa
150kDa
100kDa
70kDa
H3K14A 18kDa
20kDa
15kDa

S7.C

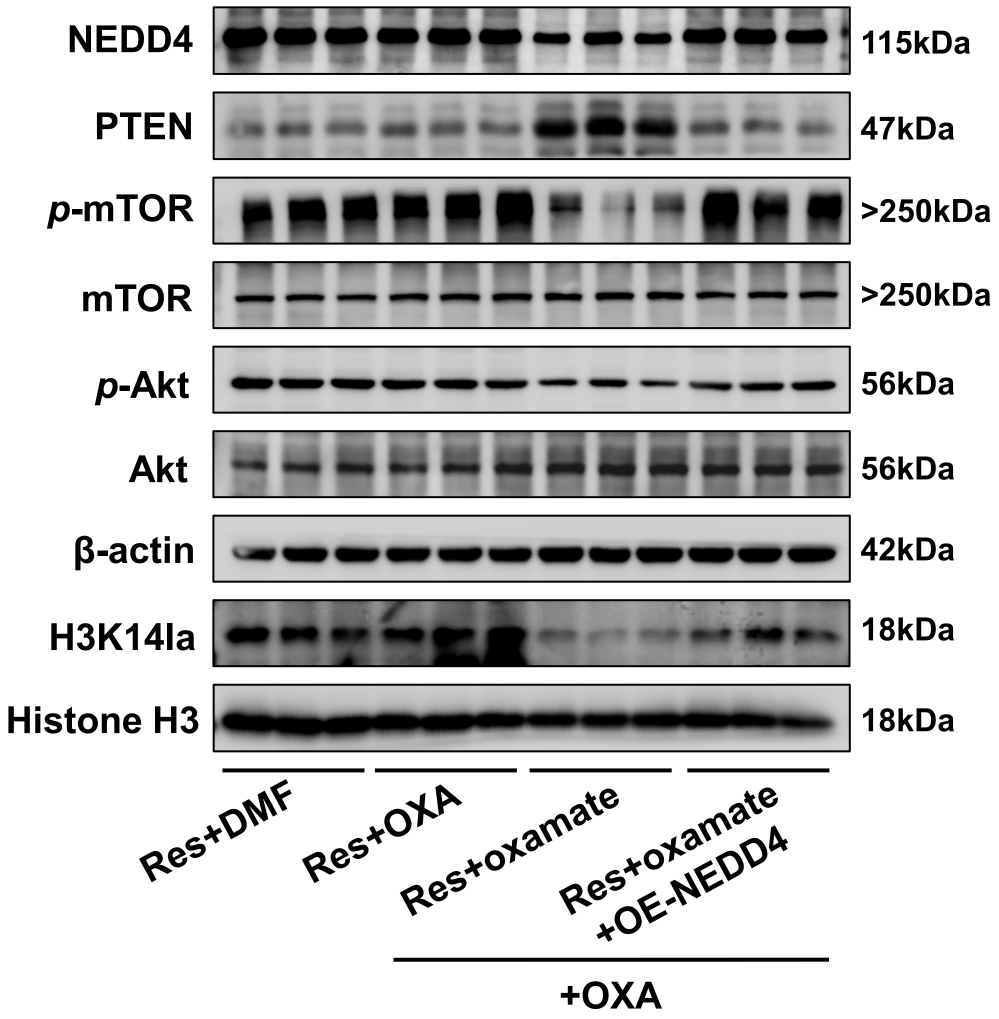
NEDD4
115kDa
PTEN
47kDa
p-mTOR
>250kDa
mTOR
>250kDa
p-Akt
56kDa
Akt
56kDa
β-actin
42kDa
H3K14la
18kDa
Histone H3
18kDa
Res+DMF
Res+OXA
Res+oxamate
Res+oxamate +OE-NEDD4
+OXA

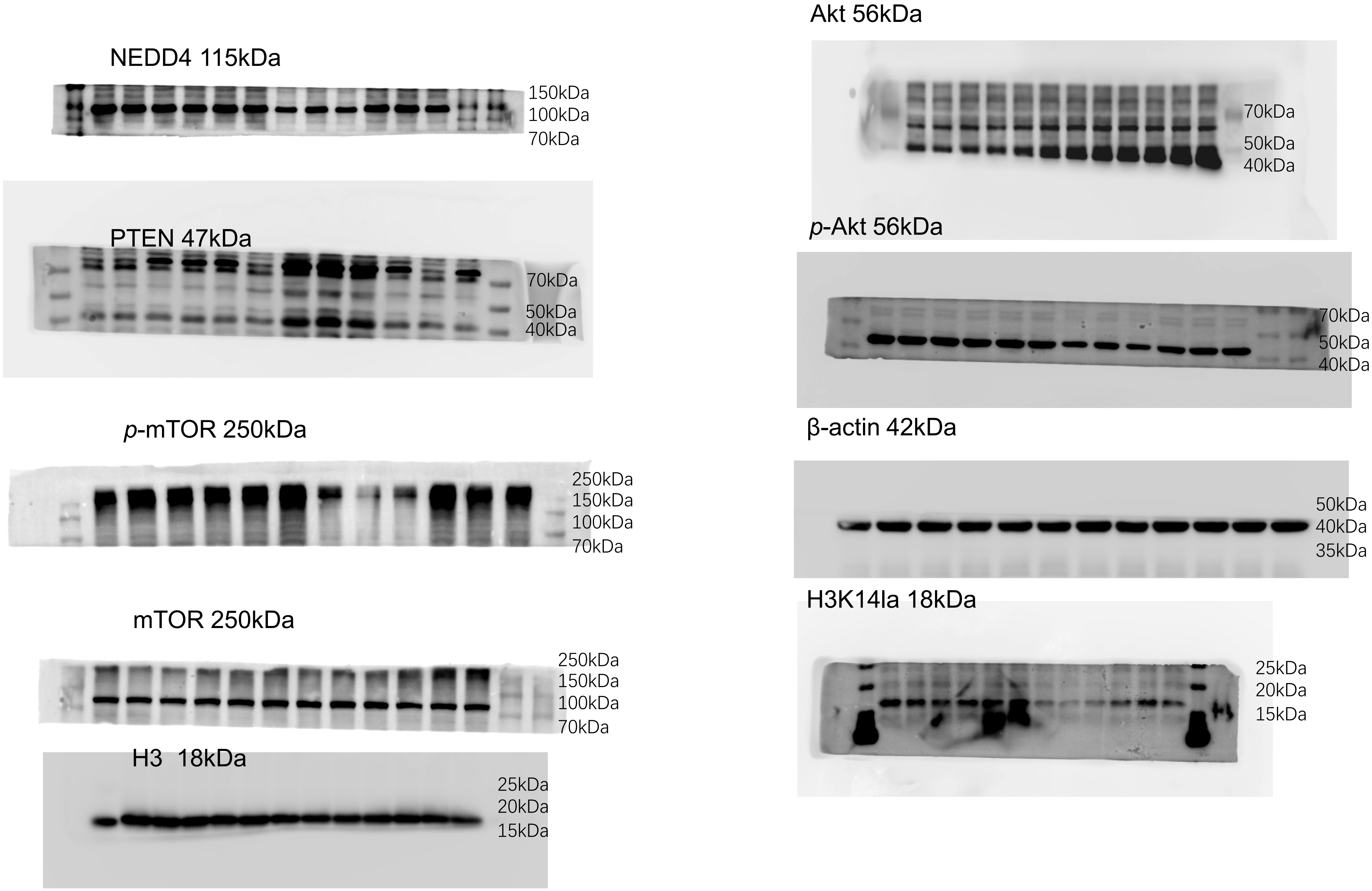
NEDD4 115kDa
150kDa
100kDa
70kDa
PTEN 47kDa
70kDa
50kDa
40kDa
p-mTOR 250kDa
250kDa
150kDa
100kDa
70kDa
mTOR 250kDa
250kDa
150kDa
100kDa
70kDa
H3 18kDa
25kDa
20kDa
15kDa
Akt 56kDa
70kDa
50kDa
40kDa
p-Akt 56kDa
70kDa
50kDa
40kDa
β-actin 42kDa
50kDa
40kDa
35kDa
H3K14la 18kDa
25kDa
20kDa
15kDa

S7.D

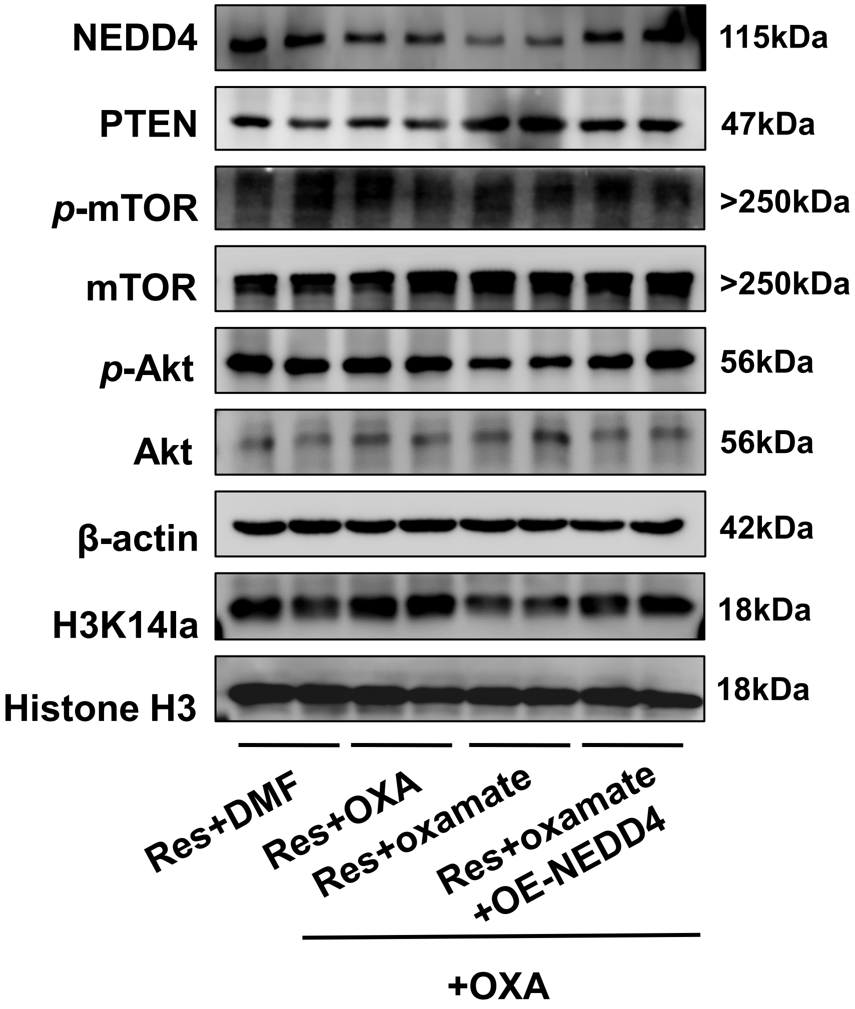
NEDD4
115kDa
PTEN
47kDa
p-mTOR
>250kDa
mTOR
>250kDa
p-Akt
56kDa
Akt
56kDa
β-actin
42kDa
H3K14la
18kDa
Histone H3
18kDa
Res+DMF
Res+OXA
Res+oxamate
Res+oxamate +OE-NEDD4
+OXA

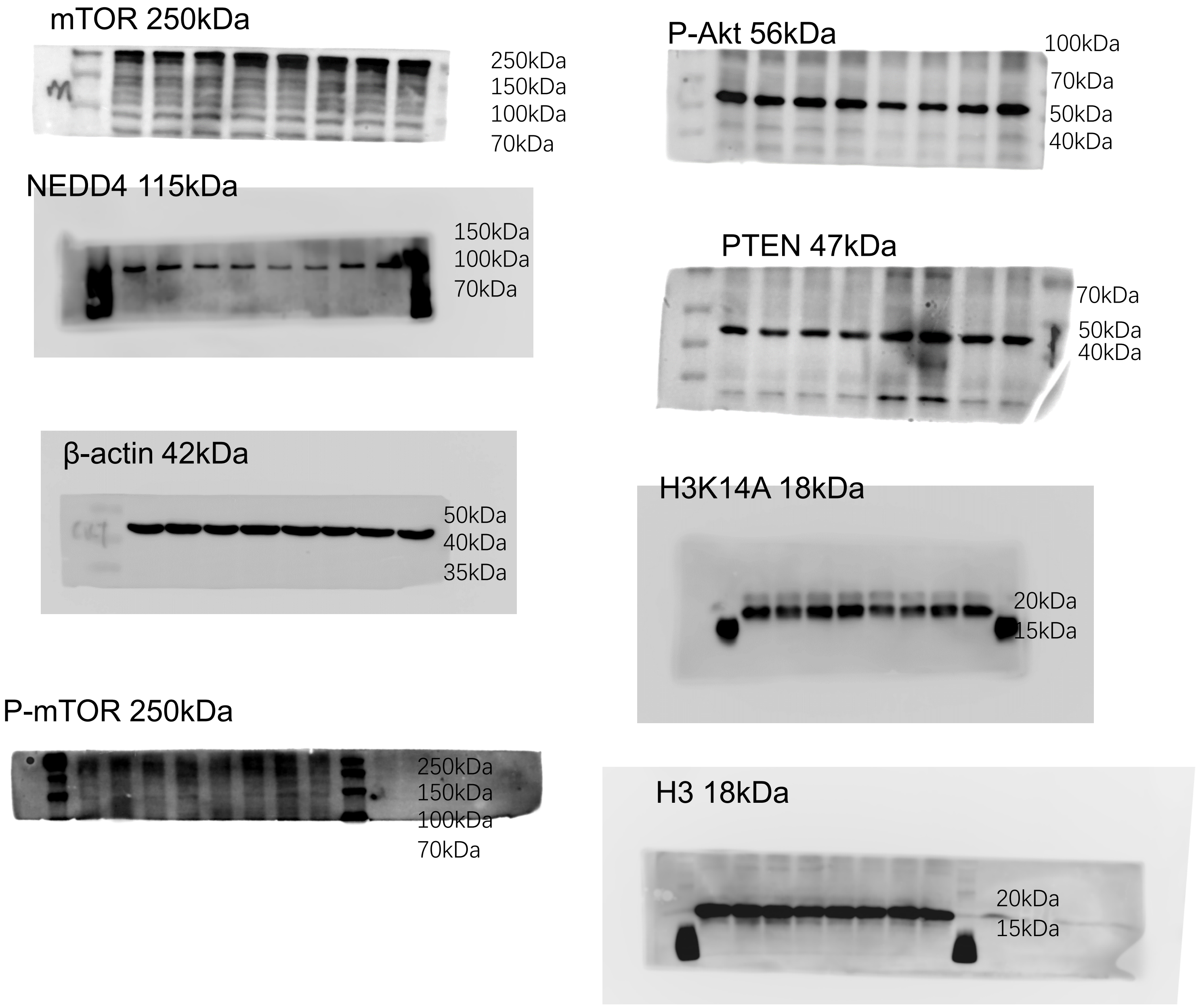
mTOR 250kDa
250kDa
150kDa
100kDa
70kDa
NEDD4 115kDa
150kDa
100kDa
70kDa
β-actin 42kDa
50kDa
40kDa
35kDa
P-mTOR 250kDa
250kDa
150kDa
100kDa
70kDa
P-Akt 56kDa
100kDa
70kDa
50kDa
40kDa
PTEN 47kDa
70kDa
50kDa
40kDa
H3K14A 18kDa
20kDa
15kDa
H3 18kDa
20kDa
15kDa
